# New synthesis of oligosaccharides modelling the M epitope of the *Brucella* O-polysaccharide

**DOI:** 10.3389/fchem.2024.1424157

**Published:** 2024-06-21

**Authors:** Yury E. Tsvetkov, Timur M. Volkov, Sergei A. Eremin, Oleg D. Sklyarov, Yuri K. Kulakov, Vadim B. Krylov, Nikolay E. Nifantiev

**Affiliations:** ^1^ Laboratory of Glycoconjugate Chemistry, N.D. Zelinsky Institute of Organic Chemistry, Russian Academy of Sciences, Moscow, Russia; ^2^ Faculty of Chemistry, M.V. Lomonosov Moscow State University, Moscow, Russia; ^3^ Russian State Centre of Quality and Standardization of Veterinary Drugs and Feeds, Moscow, Russia; ^4^ Laboratory of Brucellosis, N.F.Gamaleya National Research Center of Epidemiology and Microbiology, Moscow, Russia; ^5^ Laboratory of Synthetic Glycovaccines, N.D. Zelinsky Institute of Organic Chemistry, Russian Academy of Sciences, Moscow, Russia

**Keywords:** *Brucella*, lipopolysaccharide, O-polysaccharide, M epitope, 4-formamido, 4,6-dideoxy-D-mannose, oligosaccharides

## Abstract

Brucellosis is a dangerous zoonotic disease caused by bacteria of the genus *Brucella*. Diagnosis of brucellosis is based on the detection in animal and human sera of antibodies to the O-polysaccharide of *Brucella* lipopolysaccharide. The currently employed serodiagnosis of brucellosis relies on the use of the *Brucella* O-polysaccharide as a diagnostic antigen. However, the existence of bacterial species, which also express O-polysaccharides structurally similar to that of *Brucella*, may decrease the specificity of the brucellosis detection due to false-positive test results. It has been shown that the efficiency of the test can be significantly improved by using synthetic oligosaccharides that correspond to the so-called M epitope of the *Brucella* O-antigen. This epitope is characterized by an α-(1→3)-linkage between d-perosamine units and is unique to *Brucella*. Here we report on an efficient approach to the synthesis of oligosaccharides that model the M epitope of the *Brucella* O-polysaccharide. The approach is based on the use of the α-(1→3)-linked disaccharide thioglycoside as the key donor block. Its application allowed the straightforward assembly of a set of four protected oligosaccharides, which includes a disaccharide, two trisaccharides, and a tetrasaccharide, in five glycosylation steps. The synthesized oligosaccharides are planned to be used in the development of diagnostic tools for identifying brucellosis in humans and domestic animals, as well as a potential vaccine against it.

## Introduction

Brucellosis is a bacterial infection of domestic animals, including cattle, sheep, goats, pigs and others. It is caused by Gram-negative bacteria of the genus *Brucella*. The disease can lead to abortions, infertility, weight loss, and reduced productivity in infected animals. When humans come into contact with infected animals or their products, such as milk, contaminated meat or organs, they can become infected with brucellosis. This can lead to serious acute and chronic illness with non-specific symptoms similar to malaria or influenza, and is therefore a significant public health concern (Franco et al., 2007).

**FIGURE 1:**
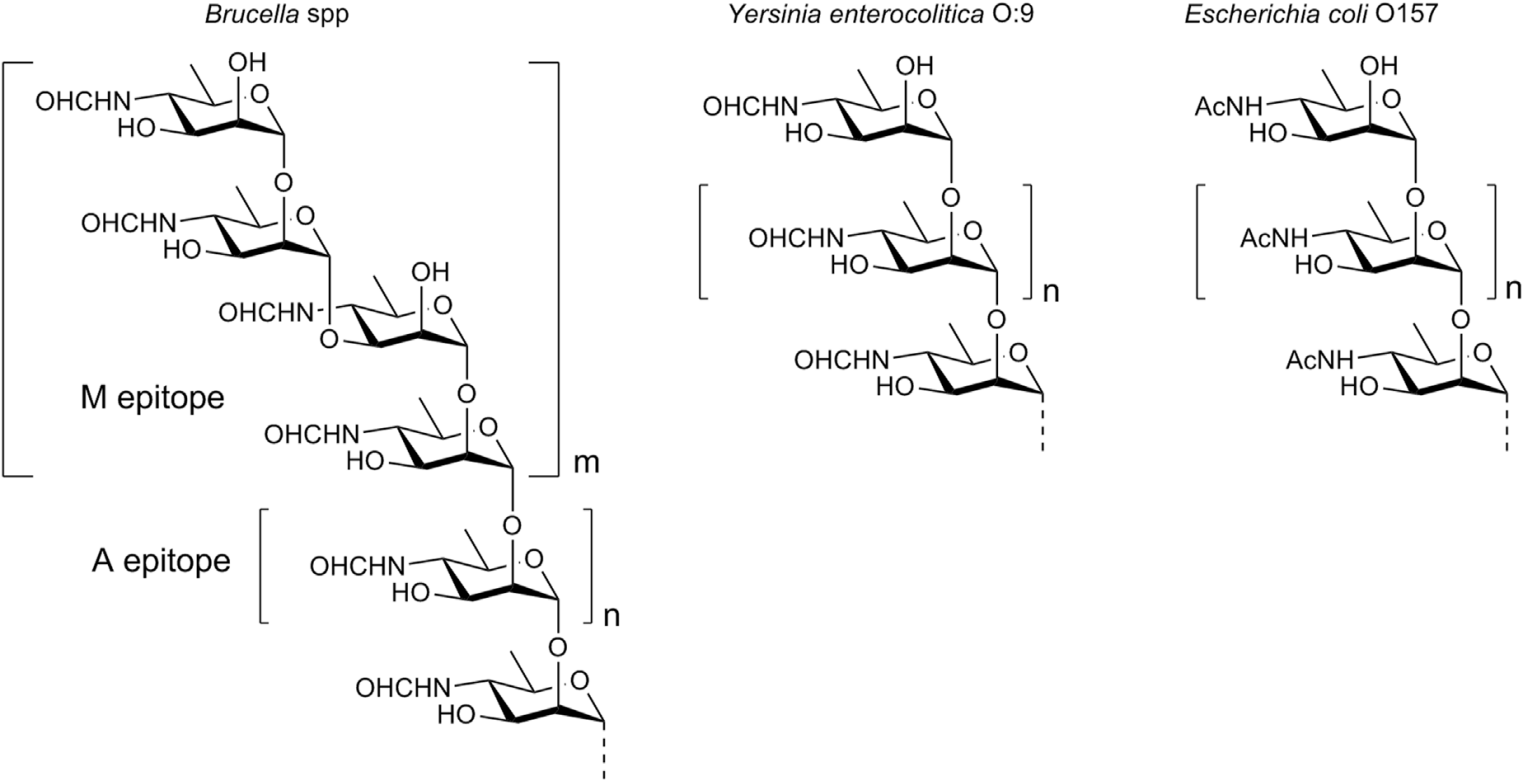
Structure of the O-polysaccharide of *Brucella* and the O-polysaccharides of other bacteria comprising N-acyl-d-perosamine.

One of the main virulence factors of *Brucella* is a lipopolysaccharide (LPS), which is located on the surface of the bacterial cell. The lipid part of LPS is embedded in the cell membrane, while the polysaccharide component (O-polysaccharide) extends outwards into the external environment, and thus determines the immunologic properties of the bacterium. O-Polysaccharide is a homopolymer of N-formylated 4-amino-4,6-dideoxy-d-mannose, also known as d-perosamine. N-Formyl-d-perosamine forms an α-(1→2)-linked linear chain terminated with one or more tetrasaccharide units that contain two α-(1→2)- and one central α-(1→3)-glycosidic bonds (Figure 1) (Kubler-Kielb and Vinogradov, 2013). The O-polysaccharide chain has a relatively low molecular weight, with a maximum of 25–30 monosaccharide units (Kubler-Kielb and Vinogradov, 2013). The antigenic determinants, represented by the α-(1→2)-linked chain and the terminal tetrasaccharide with the α-(1→3)-bond, are referred to as the A epitope (prevails in most strains of B. **a**bortus) and the M epitope (is characteristic of B. **m**elitensis), respectively.

The detection of antibodies specific to the O-polysaccharide in animal and human sera is the basis for the diagnosis of brucellosis (Nielsen, 2002; Godfroid et al., 2010). However, this biopolymer is not readily available through bacterial cultivation. Although the chemical synthesis of polysaccharides is in principal possible (Kochetkov et al., 1987; Tsvetkov et al., 1989), this task is often too complex. It is important that the specificity of the serodiagnosis using *Brucella* O-polysaccharide as a diagnostic antigen may not be high enough, as there are some bacteria that have O-polysaccharides made up of N-acylated d-perosamine. A prominent example of these bacteria is *Yersinia enterocolitica* O:9. Its O-polysaccharide consists of α-(1→2)-linked N-formyl-d-perosamine units only (Caroff et al., 1984), and is structurally identical to the A epitope of *Brucella*. Another example is *Escherichia coli* O157, whose O-polysaccharide contains α-(1→2)-linked N-acetyl-d-perosamine (Perry and Bundle, 1990). Sera obtained from animals or humans infected with such bacteria may contain antibodies that cross-react with the *Brucella* O-polysaccharide, leading to false-positive test results.

An increase in the specificity of the serodiagnosis of brucellosis can be achieved through the use of antigens related to the epitope M, which is unique to *Brucella* and is absent in the O-polysaccharides of other bacteria. It is evident that obtaining such antigens from the natural O-polysaccharide is challenging, if not impossible, and the most promising way to produce them is through chemical synthesis. Nowadays, synthetic oligosaccharides of virtually any structure are accessible and find application in immunological studies, including the development of diagnostic tools and vaccines, as a good alternative for antigenic polysaccharides (for recent examples see Argunov et al., 2015; Gening et al., 2015; Kazakova et al., 2020; Komarova et al., 2018; Krylov et al., 2019; Laverde et al., 2020; Solovev et al., 2023; Wong et al., 2020).

The use of synthetic oligosaccharides that mimic the M epitope of the *Brucella* O-polysaccharide for serodiagnosis has recently been investigated by Bundle et al. A large group of oligosaccharides with the characteristic for the M epitope (1→3)-glycoside bond between N-formyl-d-perosamine residues, was synthesized (Guiard et al., 2013; Ganesh et al., 2014; Bundle and McGiven, 2017). BSA conjugates of these synthetic oligosaccharides, particularly the (1→3)-linked disaccharide and the tetrasaccharide with the central (1→3)-glycoside bond, have shown high diagnostic sensitivity and specificity, as well as the ability to reliably differentiate between the sera of infected animals and those that give false-positive results when tested using conventional methods (McGiven et al., 2015; Bundle and McGiven, 2017).

However, the use of BSA as a carrier protein may carry risks of obtaining overestimated results. This is due to the presence in human sera of antibodies capable of recognizing BSA (Mogues et al., 2005; Sjöwall et al., 2011) and false-positive signals caused by multimeric presentation of glycoligands in the BSA conjugates (Grant et al., 2014; Temme et al., 2019). In addition, there may be a risk of non-specific interactions with hydrophobic spacers, for example, the 5-carboxypentyl spacer used in the works by Bundle et al. (Guiard et al., 2013; Ganesh et al., 2014; Mandal et al., 2017a; Mandal et al., 2017b; Bundle and McGiven, 2017).

**FIGURE 2:**
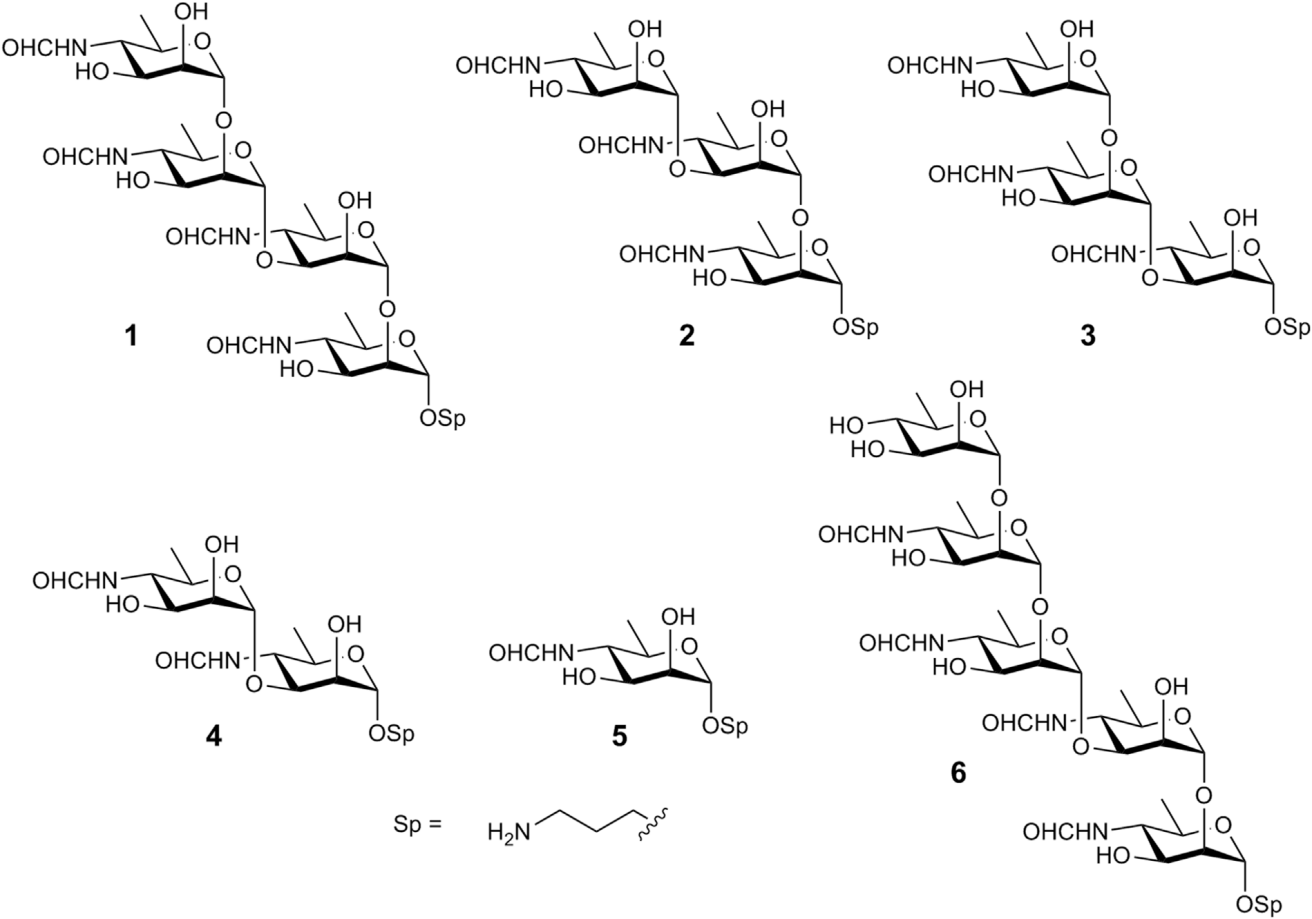
Oligosaccharides synthesized in this work.

α-(1→2)-Linked oligomers of N-(3-deoxy-L-*glycero*-tetronoyl)-D-perosamine related to the O-polysaccharide of *Vibrio cholerae* O1 have been synthesized by the Kováć’s group (Hou and Kovač, 2010; Mukherjee et al., 2019). Recently, we reported the synthesis of the (1→3)-linked disaccharide **4** (Figure 2) (Tsvetkov and Nifantiev, 2023), which corresponds to the unique structural element of the M epitope of the *Brucella* O-polysaccharide. Here, we report on the synthesis of larger oligosaccharides **1**-**3** that comprise the (1→3)-linked disaccharide as a fragment. They were prepared with the aim of developing fluorescence polarization–based and other types of diagnostic assays to identify brucellosis in animals and humans and as potential ligands for the design of corresponding vaccines. Additionally, pentasaccharide **6** has been synthesized, representing tetrasaccharide **1** devoid of terminal unsubstituted d-perosamine through its capping with a d-rhamnose residue. We have also prepared monosaccharide **5**, as it has been reported that sera from mice immunized with conjugate immunogens based on (1→2)-linked *Brucella* oligosaccharides and sera from naturally Brucella-infected cattle contained high titers of antibodies specific to the terminal d-perosamine unit (Mandal et al., 2017a; Duncombe et al., 2022). The synthesized compounds were equipped with a short 3-aminipropyl spacer group, which was thought to be less hydrophobic than the 5-carboxypentyl group used by Bundle et al. We hypothesized that the use of the 3-aminopropyl spacer would minimize hydrophobic interactions, which could interfere with antigen-antibody recognition processes.

## Results and discussion

**SCHEME 1:**
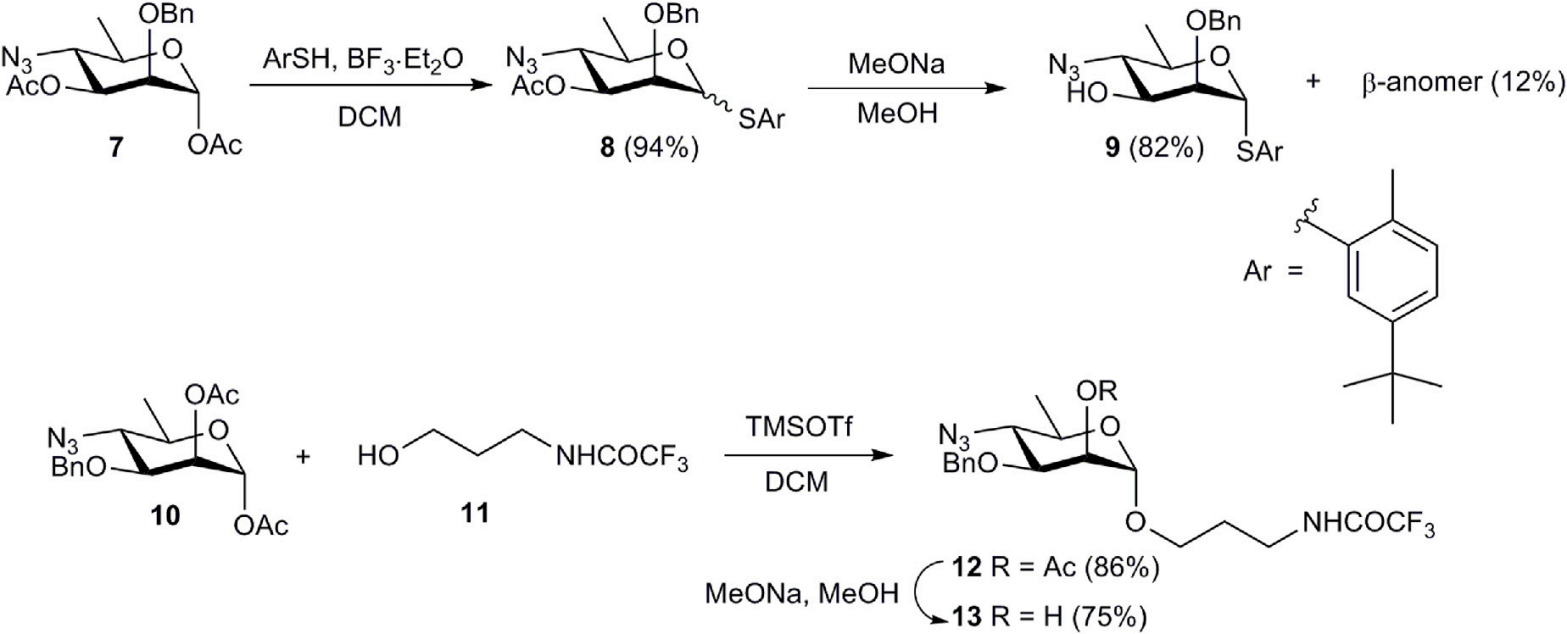
Preparation of monosaccharide acceptor blocks **9** and **13**.

Oligosaccharides **1**-**4** were synthesized using a disaccharide (1→3)-linked glycosyl donor **15** as the key synthetic block. This choice of the donor allowed the assembly of all four oligosaccharides in a straightforward manner. The synthesis of monosaccharide derivatives, which were used as glycosyl acceptors in the preparation of the target structures, is shown in Scheme 1.

Treatment of known 1,3-diacetate **7** (Mandal et al., 2017b) with 5-(*tert-*butyl)-2-methylthiophenol in the presence of BF_3_·Et_2_O produced an inseparable mixture of α- and β-thioglycosides **8**. After conventional deacetylation of **8**, the anomers were separated and pure α-anomer **9** was isolated in 82% yield. Similarly, TMSOTf-promoted glycosylation of alcohol **11** with 1,2-diacetate **10** (Bundle et al., 1988) and subsequent deacetylation of glycoside **12** afforded glycosyl acceptor **13**.

**SCHEME 2:**
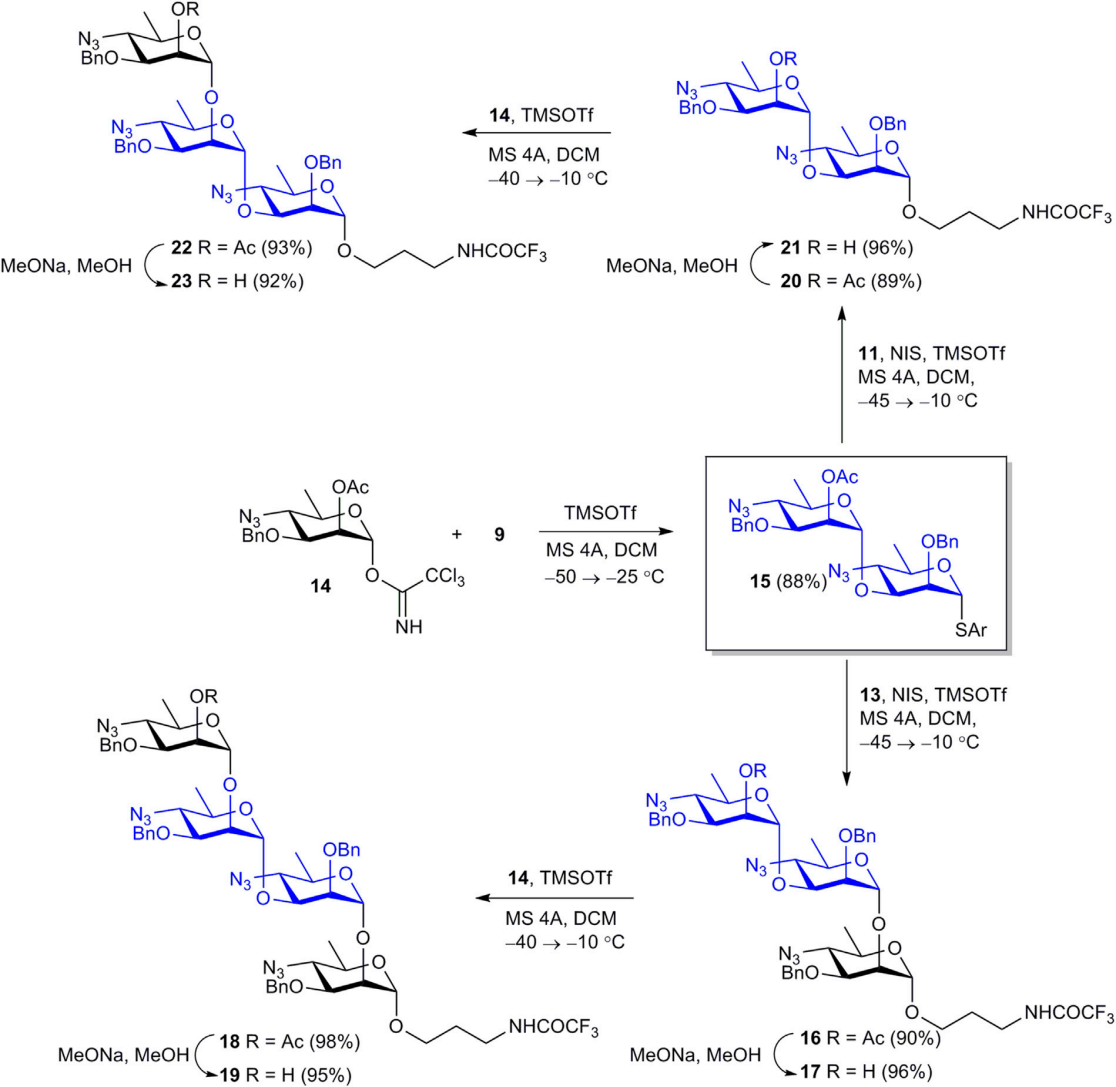
Assembly of protected oligosaccharides **16**, **18**, **20**, **22**.

Using acceptors **9** and **13**, and the known trichloroacetimidate **14** (Ariosa-Alvarez et al., 1998) as a glycosyl donor, all oligosaccharides were assembled in five glycosylation steps (Scheme 2).

Orthogonal glycosylation of thioglycoside acceptor **9** with imidate **14** smoothly gave key intermediate **15** in 88% yield. Further NIS–TMSOTf-promoted glycosylation of acceptors **11** and **13** with thioglycoside **15** efficiently produced disaccharide **20** and trisaccharide **16**. The ^1^ *J*_C-1,H-1_ coupling constant values (170–172 Hz) for the 3-substituted monosaccharide residues in products **16** and **20** confirmed the α-configuration of the newly formed glycoside bonds (Bock and Pedersen, 1974). Despite the presence of a non-participating benzyl group at O-2, donor **15** ensured high α-stereoselectivity of glycosylation, in agreement with published data (Hou and Kovač, 2010). Removal of sole acetyl groups from **16** and **20** provided glycosyl acceptors **17** and **21** almost quantitatively. Final glycosylation of these acceptors with imidate **14** afforded tetrasaccharide **18** and trisaccharide **22** in excellent yields. Deacetylation of **18** and **22** gave alcohols **19** and **23**. The structure of protected oligosaccharides **15**-**23** was confirmed by data of fully assigned ^1^H and ^13^C NMR spectra (see Supplementary Material) and high resolution MS.

**SCHEME 3:**
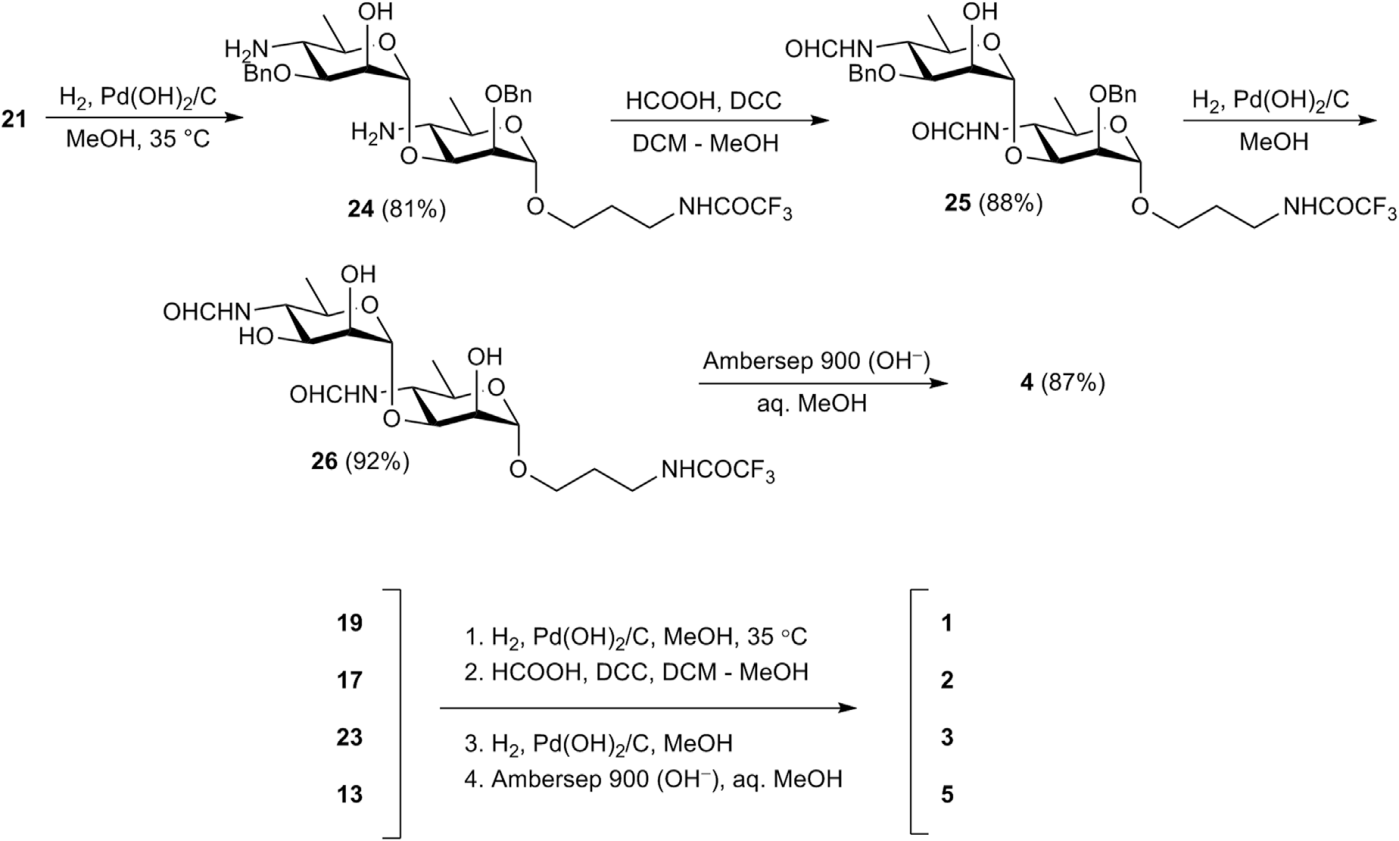
Synthesis of free mono-(**5**) and oligosaccharides (**1**–**4**).

Then oligosaccharides **17**, **19**, **21**, **23**, and monosaccharide **13** were transformed to final products **1**-**5**. The reaction sequence employed for this transformation included reduction of azide groups, N-formylation, hydrogenolytic debenzylation and removal of the N-trifluoroacetyl group. This sequence is shown in Scheme 3 in relation to disaccharide **21**. Intermediate products **24**-**26** on the way from **21** to unprotected **4** were characterized by high resolution mass-spectrometry. Each of the four protected compounds **13**, **17**, **19**, and **23** was processed similarly (for details see Supplementary Material).

As hydrogen sulfide applied by Bundle et al. for the reduction of azide moieties (Guiard et al., 2013; Ganesh et al., 2014; Mandal et al., 2017b; Bundle and McGiven, 2017) is toxic and has an unpleasant odor, we used catalytic hydrogenation over Pd(OH)_2_/C at this step instead. Thus, catalytic reduction of diazide **21** proceeded with only minor affecting benzyl protections (Ariosa-Alvarez et al., 1998) and provided, after chromatographic purification, diamine **24** in 81% yield. N-Formylation of the latter with formic anhydride generated *in situ* from formic acid and DCC produced bis(formamide) **25** (88%), which was subjected to catalytic debenzylation with the formation of triol **26** (92%). Final basic removal of the N-trifluoroacetyl group and purification by size-exclusion chromatography gave target 3-aminopropyl glycoside **4** in 87% yield.

*E/Z*-Isomerism of the formamido groups results in the existence of compounds **1**-**5** as mixtures of 2^n^ isomers, where n is the number of monosaccharides in the molecule (Peters et al., 1990). This strongly complicated the full assignment of NMR spectra, making it virtually impossible for oligosaccharides larger than the disaccharide. Nevertheless, the NMR data of **1**-**5** provided confirmation of the presence of the main functionalities such as formyl groups, anomeric centers, the spacer moiety, etc. (see Supplementary Material). Particularly, signals for *Z*- (δ_H_ 8.24–8.18 ppm; δ_C_ 166.1, 165.8 ppm) and *E*-isomers (δ_H_ 8.06–7.99 ppm; δ_C_ 169.0, 168.9 ppm) of the formyl groups, four anomeric centers of the prevailing isomer (δ_H_ 5.09, 5.02, 4.98, 4.92 ppm; δ_C_ 103.4, 102.9, 101.8; 99.6 ppm), and C-4 of *E*- (δ_C_ 58.2, 58.0, 57.9, 56.7 ppm) and *Z*-isomers (53.3, 53.1, 52.9, 52.1 ppm) were observed, *inter alia*, in the ^1^H and ^13^C NMR spectra of tetrasaccharide **1**.

**SCHEME 4:**
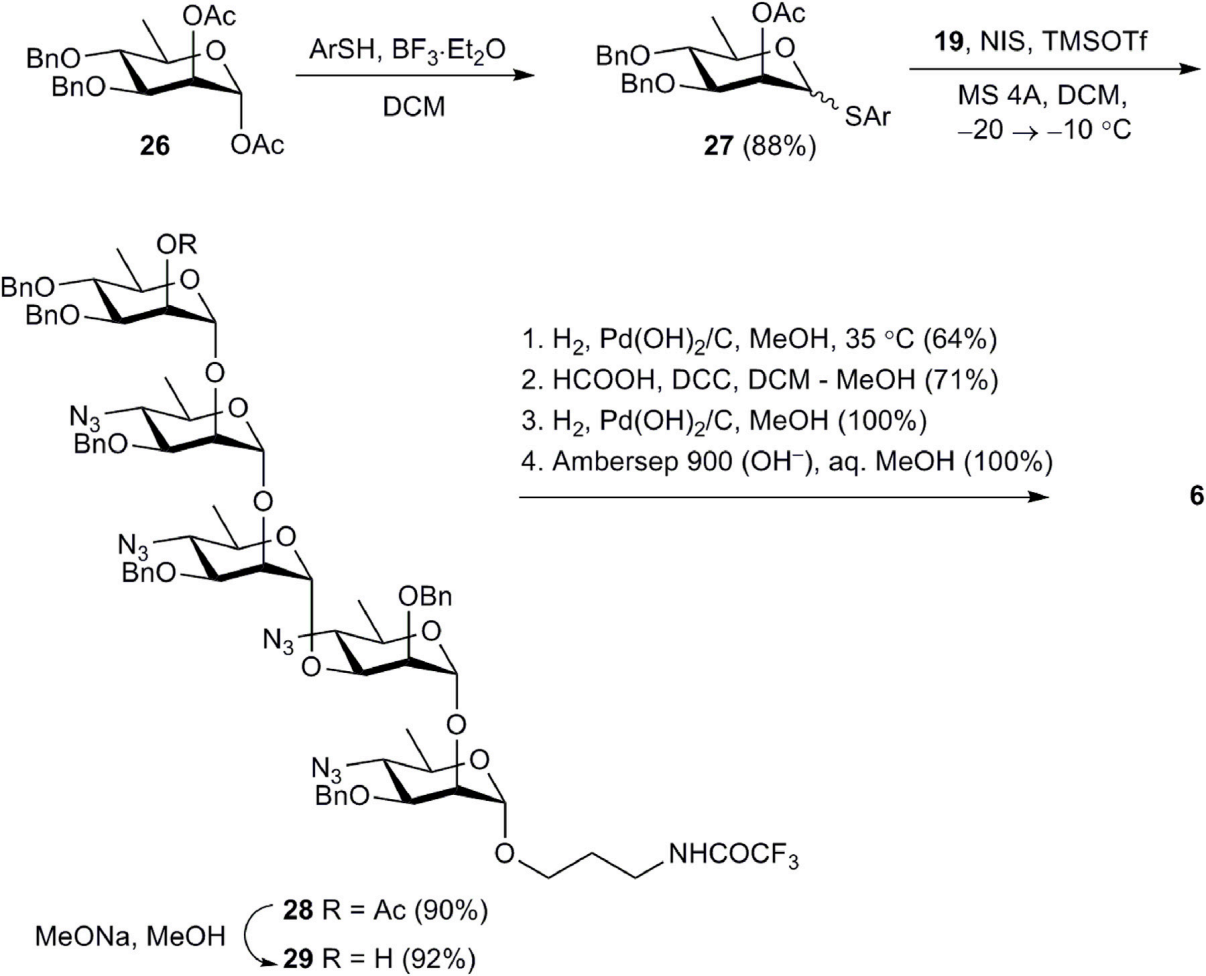
Synthesis of pentasaccharide **6**.

Pentasaccharide **6**, in which the d-perosamine chain is terminated by a d-rhamnose unit, was prepared from tetrasaccharide acceptor **19** (Scheme 4). This pentasaccharide was thought to lose the ability to be recognized by antibodies specific to the terminal unsubstituted d-perosamine residue (Mandal et al., 2017a). Thioglycoside **27**, which was used as the glycosyl donor, was synthesized by the reaction of known 1,2-diacetate **26** (Zhang et al., 2015) with 5-(*tert-*butyl)-2-methylthiophenol in the presence of BF_3_·Et_2_O. Following NIS–TMSOTf-promoted glycosylation of **19** with **27** produced pentasaccharide **28** in excellent yield. After deacetylation of **28**, product **29** was subjected to deprotection with the use of the common reaction sequence (Scheme 4) resulting in the formation of target pentasaccharide **6**.

## Conclusion

In conclusion, an efficient approach to the synthesis of oligosaccharides related to the M epitope of the *Brucella* O-polysaccharide has been developed. The approach is based on the use of the (1→3)-linked disaccharide thioglycoside as the key donor block. This disaccharide donor mimics the unique structural fragment of the M epitope with the (1→3)-linkage between two N-formyl-d-perosamine residues. Its application allowed the straightforward assembly of a set of four protected oligosaccharides, which comprised the disaccharide, two trisaccharides, and the tetrasaccharide, in five glycosylation steps. Following deprotection included reduction of azido groups, N-formylation, O-debenzylation, and de-N-trifluoroacetylation and produced free oligosaccharides as 3-aminopropyl glycosides. Using the partially protected tetrasaccharide as the glycosyl acceptor, the pentasaccharide devoid of terminal d-perosamine through its capping with a d-rhamnose residue has been also prepared. The synthesized oligosaccharides will be used for development of a diagnostic assay to identify brucellosis in animals and humans as well as the potential ligands to design vaccine candidates.

## Experiment

### General

NMR spectra were recorded on a Bruker Avance 600 NMR spectrometer. Protected oligosaccharides were measured in chloroform-d (CDCl_3_), and ^1^H NMR chemical shifts were referenced to the solvent residual signal (δ_H_ 7.27). ^13^C chemical shifts were referenced to the central resonance of CDCl_3_ (δ_C_ 77.0). Free oligosaccharides were measured in deuterium oxide (D_2_O) with suppression of the HOD signal. Acetone (δ_H_ 2.225, δ_C_ 31.45) was used as an internal standard. Signal assignment was made using COSY, TOCSY, HSQC, and ROESY experiments. In the presentation of NMR data, monosaccharide residues in oligosaccharides are denoted by the capital letters (A, B, C, etc.) starting from the reducing end. NMR spectra of synthesized compounds are presented in the Supplementary Material. High-resolution mass spectrometry (HRMS) with electrospray ionization (ESI) was performed on a MicrOTOF II (Bruker Daltonics) instrument. Optical rotations were measured using a JASCO P-2000 polarimeter at 20°C–25°C in chloroform (protected oligosaccharides) or water (free oligosaccharides). TLC was performed on Silica Gel 60 F_254_ plates (Merck) and visualization was accomplished using UV light or by charring at ∼150°C with orcinol–phosphoric acid (180 mg of orcinol in a mixture of 85% H_3_PO_4_ (10 mL), ethanol (5 mL), and water (85 mL)). Column chromatography was carried out using Silica Gel 60 (40–63μm; Merck Millipore). Gel-permeation chromatography of free oligosaccharides was performed on a Toyopearl TSK HW-40(S) column (2.8 × 80 cm) in 0.1 M acetic acid. A K-2401 refractive index detector (Knauer) was used to monitor gel-permeation chromatography. All moisture-sensitive reactions were carried out using dry solvents under dry argon. Powdered molecular sieve 4 Å was activated at 300°C under vacuum (∼1 mbar) for 30 min directly prior to the reaction. Solutions were concentrated under reduced pressure using a rotatory evaporator at 40°C (bath temperature).

**5-(*tert*-Butyl)-2-methylphenyl 4-azido-2-*O*-benzyl-4,6-dideoxy-1-thio-α-** **d** **-mannopyranoside (9)**. BF_3_·Et_2_O (84 μL, 0.67 mmol) was added to a solution of 1,3-diacetate **7** (203 mg, 0.56 mmol) and 5-(*tert*-butyl)-2-methylthiophenol (153 μL, 0.84 mmol) in DCM (4 mL) upon chilling in an ice bath. The mixture was stirred for 10 min. with chilling and then at room temperature for 1.5 h. The resulting mixture was diluted with chloroform (50 mL), washed with water (30 mL) and saturated aqueous NaHCO_3_ (30 mL). The solvent was evaporated and the residue was purified by column chromatography (petroleum ether–EtOAc, 0→5%) to give thioglycoside **8** (255 mg, 94%) as a colorless syrup; α,β ratio ∼6.3:1. ^1^H NMR (600 MHz,CDCl_3_): δ 7.62–7.13 (m, 9.3 H, Ar), 5.44 (s, 1 H, H-1_α_), 5.15 (dd, 1 H, *J*_2,3_ = 3.2 Hz, *J*_3,4_ = 10.4 Hz, H-3_α_), 4.91 (d, 0.16 H, *J* = 11.8 Hz, PhC*Ha*Hb_β_) 4.84 (dd, 0.16 H, *J*_2,3_ = 3.2 Hz, *J*_3,4_ = 10.2 Hz, H-3_β_), 4.78 (d, 0.16 H, *J* = 11.8 Hz, PhCHa*Hb*_β_), 4.73 (s, 1 H, H-1_β_), 4.70 (d, 1 H, *J* = 12.2 Hz, PhC*Ha*Hb_α_), 4.52 (d, 1 H, *J* = 12.2 Hz, PhCHa*Hb*_α_), 4.25 (d, 0.16 H, *J*_2,3_ = 3.2 Hz, H-2_β_), 4.16–4.11 (m, 2 H, H-2_α_, H-5_α_), 3.76–3.71 (m, 1.16 H, H-4_α_, H-4_β_), 3.30 (dq, 0.16 H, *J*_5,6_ = 6.3 Hz, *J*_4,5_ = 9.8 Hz, H-5_β_), 2.38 (s, 0.5 H, Ar-C*H*_*3*β_), 2.36 (s, 3 H, Ar-C*H*_*3*α_), 2.10 (s, 3 H, CH_3_CO_α_), 2.05 (s, 0.5 H, CH_3_CO_β_), 1.46 (d, 0.5 H, *J*_6,5_ = 6.3 Hz, H-6_β_), 1.40 (d, 3 H, *J*_6,5_ = 6.1 Hz, H-6_α_), 1.32 (s, 10.4 H, Ar-C(C*H*_*3*_)_αβ_). ^13^C NMR (150 MHz, CDCl_3_): δ 169.9 (CH_3_ *C*O), 149.8, 137.2, 136.4, 132.7, 130.0, 129.8, 129.4, 129.0, 128.5, 128.1, 128.0, 124.8 (Ar), 87.1 (C-1_α_), 84.8 (C-1_β_), 77.6 (C-2_β_), 76.4 (C-2_α_), 76.0 (Ph*C*H_2β_), 75.9 (C-3_β_), 75.3 (C-5_β_), 72.7 (C-3_α_), 72.5 (Ph*C*H_2α_), 68.3 (C-5_α_), 62.9 (C-4_α_), 62.3 (C-4_β_), 31.2 (C(*C*H_3_)_3_), 20.9 (*C*H_3_CO), 20.2 (*C*H_3_-Ar), 18.7 (C-6_β_), 18.4 (C-6_α_).

1 M Sodium methoxide in MeOH (50 μL) was added to a solution of 3-acetate **8** (255 mg, 0.53 mmol) in MeOH (5 mL), and the mixture was stirred for 2 h at room temperature. The mixture was neutralized with Amberlite IR-120 (H^+^), the resin was filtered off and washed with MeOH (4 × 4 mL), and the filtrate was concentrated. The residue was subjected to column chromatography (petroleum ether–EtOAc, 2→8%) to produce α-anomer **9** (192 mg, 82%) and β-anomer (29 mg, 12%).

α-Anomer **9**, colorless syrup, [α]_D_ +95 (c 1, СHCl_3_). ^1^H NMR (600 MHz, CDCl_3_): δ 7.56–7.16 (m, 8 H, Ar), 5.53 (s, 1 H, H-1), 4.77 (d, 1 H, *J* = 11.8 Hz, PhC*Ha*Hb), 4.55 (d, 1 H, *J* = 11.8 Hz, PhCHa*Hb*), 4.07 (dq, 1 H, *J*_4,5_ = 9.9 Hz, *J*_5,6_ = 6.3 Hz, H-5), 4.02 (d, 1 H, *J*_2,3_ = 3.7 Hz, H-2), 3.94 (dd, 1 H, *J*_2,3_ = 3.7 Hz, *J*_3,4_ = 9.8 Hz, H-3), 3.40 (t, 1 H, *J* = 9.8 Hz, H-4), 2.37 (s, 3 H, Ar-C*H*_*3*_), 1.37 (d, 3 H, *J*_6,5_ = 6.3 Hz, H-6), 1.33 (s, 9 H, Ar-C(C*H*_*3*_)). ^13^C NMR (150 MHz, CDCl_3_): δ 149.8, 136.9, 136.4, 132.6, 130.0, 129.6, 128.7, 128.3, 128.1, 125.0 (Ar), 84.3 (C-1), 78.9 (C-2), 72.3 (Ph*C*H_2_), 70.9 (C-3), 67.9 (C-5), 66.8 (C-4), 31.3 (C(*C*H_3_)_3_), 20.1 (*C*H_3_-Ar), 18.3 (C-6). HRMS (ESI): calcd. for C_24_H_31_N_3_O_3_S [M + Na]+ *m/z* 464.1978; found *m/z* 464.1968.

β-Anomer, colorless syrup, [α]_D_ –96 (c 0.5, СHCl_3_). ^1^H NMR (600 MHz, CDCl_3_): δ 7.63–7.14 (m, 8 H, Ar), 5.16 (d, 1 H, *J* = 11.5 Hz, PhC*Ha*Hb), 4.80 (d, 1 H, *J* = 11.5 Hz, PhCHa*Hb*), 4.72 (s, 1 H, H-1), 4.10 (d, 1 H, *J*_2,3_ = 3.5 Hz, H-2), 3.61 (dd, 1 H, *J*_2,3_ = 3.6 Hz, *J*_3,4_ = 9.7 Hz, H-3), 3.35 (t, 1 H, *J* = 9.8 Hz, H-4), 3.23 (dq, 1 H, *J*_4,5_ = 9.8 Hz, *J*_5,6_ = 6.3 Hz, H-5), 2.40 (s, 3 H, Ar-C*H*_*3*_), 1.44 (d, 3 H, *J*_6,5_ = 6.3 Hz, H-6), 1.33 (s, 9 H, Ar-C(C*H*_*3*_)). ^13^C NMR (150 MHz, CDCl_3_): δ 149.6, 137.5, 135.7, 133.6, 129.8, 128.7, 128.5, 128.3, 128.1, 124.6 (Ar), 87.1 (C-1), 80.1 (C-2), 76.5 (Ph*C*H_2_), 75.2 (C-5), 74.3 (C-3), 66.2 (C-4), 31.3 (C(*C*H_3_)_3_), 20.2 (*C*H_3_-Ar), 18.7 (C-6). HRMS (ESI): calcd. for C_24_H_31_N_3_O_3_S [M + Na]^+^ *m/z* 464.1978; found *m/z* 464.1981.

**3-Trifluoroacetamidopropyl 2-*O*-acetyl-4-azido-3-*O*-benzyl-4,6-dideoxy-α-** **d** **-mannopyranoside (12).** TMSOTf (0.59 mL, 3.24 mmol) was added to a chilled (ice bath) solution of 1,2-diacetate **10** (906 mg, 2.49 mmol) and alcohol **11** (554 mg, 3.24 mmol) in DCM (15 mL). The mixture was stirred for 20 min. upon chilling and then 2.5 h at room temperature. After dilution with DCM (100 mL), the mixture was washed with saturated aqueous NaHCO_3_ (50 mL) and water (2 × 50 mL), and concentrated. The residue was purified by column chromatography (petroleum ether–EtOAc, 15→30%) to yield glycoside **12** (1.02 g, 86%) as a colorless syrup, , [α]_D_ +88 (c 1, СHCl_3_). ^1^H NMR (600 MHz, CDCl_3_): δ 7.39–7.29 (m, 5 H, Ar), 6.77 (br. s, 1 H, CF_3_CONH), 5.32 (br. s, 1 H, H-2), 4.76 (s, 1 H, H-1), 4.68 (d, 1 H, *J* = 11.0 Hz, PhC*Ha*Hb), 4.54 (d, 1 H, *J* = 11.0 Hz, PhCHa*Hb*), 3.82–3.78 (m, 1 H, OC*Ha*HbCH_2_CH_2_N), 3.77 (dd, 1 H, *J*_2,3_ = 3.3 Hz, *J*_3,4_ = 9.8 Hz, H-3), 3.54–3.45 (m, 4 H, OCHa*Hb*CH_2_C*H*_*2*_N, H-5), 3.44 (t, 1 H, *J* = 9.8 Hz, H-4), 2.14 (s, 3 H, CH_3_CO), 1.96–1.84 (m, OCH_2_C*H*_*2*_CH_2_N), 1.34 (d, 3 H, *J*_6,5_ = 6.1 Hz, H-6). ^13^C NMR (150 MHz, CDCl_3_): δ 170.2 (CH_3_ *C*O), 137.1, 128.4, 128.2, 128.0 (Ar), 97.9 (C-1), 76.2 (C-3), 71.7 (Ph*C*H_2_), 67.3 (C-2), 76.2 (C-5), 66.1 (O*C*H_2_CH_2_CH_2_N), 63.8 (C-4), 38.1 (OCH_2_CH_2_ *C*H_2_N), 28.3 (OCH_2_ *C*H_2_CH_2_N), 20.9 (*C*H_3_CO), 18.4 (C-6). HRMS (ESI): calcd. for C_20_H_25_F_3_N_4_O_6_ [M + Na]^+^ *m/z* 497.1618; found *m/z* 497.1609.

**3-Trifluoroacetamidopropyl 4-azido-3-*O*-benzyl-4,6-dideoxy-α-** **d** **-mannopyranoside (13).** 1 M Sodium methoxide in MeOH (100 μL) was added to a solution of 2-acetate **12** (1.02 g, 2.15 mmol) in MeOH (15 mL) and the mixture was stirred for 5 h at room temperature. Amberlite IR-120 (H^+^) was added until neutrality, the resin was filtered off and washed with MeOH (5 × 4 mL), and the filtrate was concentrated. Column chromatography of the residue (toluene–EtOAc, 15→30%) provided compound **13** (700 mg, 75%) as a colorless syrup, [α]_D_ +104 (c 1, СHCl_3_). ^1^H NMR (600 MHz, CDCl_3_): δ 7.43–7.33 (m, 5 H, Ar), 6.82 (br. s, 1 H, CF_3_CONH), 4.83 (s, 1 H, H-1), 4.71 (d, 1 H, *J* = 11.0 Hz, PhC*Ha*Hb), 4.67 (d, 1 H, *J* = 11.0 Hz, PhCHa*Hb*), 3.99 (br. s, 1 H, H-2), 3.85–3.80 (m, 1 H, OC*Ha*HbCH_2_CH_2_N), 3.68 (dd, 1 H, *J*_2,3_ = 3.3 Hz, *J*_3,4_ = 9.3 Hz, H-3), 3.56–3.42 (m, 5 H, OCHa*Hb*CH_2_C*H*_*2*_N, H-4, H-5), 2.58 (br. s, 1 H, OH), 1.94–1.83 (m, OCH_2_C*H*_*2*_CH_2_N), 1.34 (d, 3 H, *J*_6,5_ = 5.9 Hz, H-6). ^13^C NMR (150 Hz, CDCl_3_): δ 137.1, 128.6, 128.2, 128.0 (Ar), 99.2 (C-1), 78.5 (C-3), 72.1 (Ph*C*H_2_), 67.1 (C-2), 67.0 (C-5), 66.3 (O*C*H_2_CH_2_CH_2_N), 63.8 (C-4), 38.4 (OCH_2_CH_2_ *C*H_2_N), 28.2 (OCH_2_ *C*H_2_CH_2_N), 18.4 (C-6). HRMS (ESI): calcd. for C_18_H_23_F_3_N_4_O_5_ [M + Na]^+^ *m/z* 455.1513; found *m/z* 455.1506.

**4-(*tert*-Butyl)-2-methylphenyl 2-*O*-acetyl-4-azido-3-*O*-benzyl-4,6-dideoxy-α-d-mannopyranosyl-(1→3)-4-azido-2-*O*-benzyl-4,6-dideoxy-1-thio-α-d-mannopyranoside (15)**. A mixture of acceptor **9** (168 mg, 0.381 mmol), donor **14** (204 mg, 0.438 mmol) and mol. sieve 4 Å (400 mg) in DCM (6 mL) was stirred for 1.5 h at room temperature and cooled to −55°C. TMSOTf (16 μL, 0.088 mmol) was added and the mixture was stirred for 30 min. at −55 ÷ −50°C. Then, over the next 30 min., the temperature was gradually increased to −25°C and the mixture was stirred at this temperature for another 30 min. The reaction was quenched by adding Et_3_N (50 μL), the mixture was diluted with chloroform (50 mL), the solids were filtered off through a Celite layer and washed with chloroform. The filtrate was washed with water (50 mL), and the solvent was evaporated. The residue was subjected to gel-permeation chromatography on a Bio-Beads S-X3 column (3.5 × 70 cm) in toluene; fractions containing disaccharide **15** were pooled and concentrated. The product obtained was additionally purified by silica gel column chromatography (petroleum ether–EtOAc, 5→8%) to produce disaccharide **15** (251 mg, 88%) as a colorless syrup, [α]_D_ +129 (c 1, СHCl_3_). ^1^H NMR (600 MHz, CDCl_3_): δ 7.52–7.14 (m, 13 H, Ar), 5.53 (br. s, 1 H, H-2^B^), 5.48 (s, 1 H, H-1^A^), 5.12 (s, 1 H, H-1^B^), 4.72 (d, 1 H, *J* = 11.0 Hz, PhC*Ha*Hb), 4.68 (d, 1 H, *J* = 12.0 Hz, PhC*Ha*Hb**′**), 4.54 (d, 1 H, *J* = 11.0 Hz, PhCHa*Hb*), 4.53 (d, 1 H, *J* = 12.0 Hz, PhCHa*Hb*′), 4.04 (dq, 1 H, *J*_4,5_ = 9.8 Hz, *J*_5,6_ = 6.1 Hz, H-5^A^), 4.01 (br. s, 1 H, H-2^A^), 3,96 (dd, 1 H, *J*_2,3_ = 3.0 Hz, *J*_3,4_ = 10.0 Hz, H-3^A^), 3.88 (dd, 1 H, *J*_2,3_ = 3.2 Hz, *J*_3,4_ = 9.8 Hz, H-3^B^), 3.72 (t, 1 H, *J* = 9.8 Hz, H-4^A^), 3.67 (dq, 1 H, *J*_4,5_ = 10.2 Hz, *J*_5,6_ = 6.3 Hz, H-5^B^), 3.46 (t, 1 H, *J* = 10.0 Hz, H-4^B^), 2.36 (s, 3 H, Ar-C*H*_*3*_), 2.17 (s, 3 H, CH_3_CO), 1.39 (d, 3 H, *J*_6,5_ = 6.1 Hz, H-6^A^), 1.32 (s, 9 H, Ar-C(C*H*_*3*_)), 1.28 (d, 3 H, *J*_6,5_ = 6.3 Hz, H-6^B^). ^13^C NMR (150 MHz, CDCl_3_): δ 170.0 (CH_3_ *C*O), 149.9, 137.2, 136.4, 132.6, 130.0, 129.4, 128.6, 128.4, 128.3, 128.0, 127.9, 127.5, 125.0 (Ar), 99.8 (C-1^B^), 84.3 (C-1^A^), 79.6 (C-3^A^), 78.5 (C-2^A^), 75.8 (C-3^B^), 71.7, 71.5 (2 Ph*C*H_2_), 68.5 (C-5^A^), 67.9 (C-5^B^), 67.3 (C-2^B^), 64.4 (C-4^A^), 63.8 (C-4^B^), 31.3 (C(*C*H_3_)_3_), 20.9 (CH_3_CO), 20.2 (*C*H_3_-Ar), 18.6 (C-6^B^), 18.4 (C-6^A^). HRMS (ESI): calcd. for C_39_H_48_N_6_O_7_S [M + NH_4_]^+^ *m/z* 762.3643; found *m/z* 762.3636.

**3-Trifluoroacetamidopropyl 2-*O*-acetyl-4-azido-3-*O*-benzyl-4,6-dideoxy-α-d-mannopyranosyl-(1→3)-4-azido-2-*O*-benzyl-4,6-dideoxy-α-d-mannopyranosyl-(1→2)-4-azido-3-*O*-benzyl-4,6-dideoxy-α-d-mannopyranoside (16).** A mixture of donor **15** (251 mg, 0.337 mmol), acceptor **13** (132 mg, 0.306 mmol) and mol. sieve 4 Å (500 mg) in DCM (6 mL) was stirred for 1 h at room temperature, and then was cooled to −45°C. NIS (152 mg, 0.674 mmol) and TMSOTf (12 μL) were added and the mixture was stirred for 1 h, gradually increasing the temperature to −10°C. The reaction was quenched by adding pyridine (50 μL), the mixture was diluted with chloroform (10 mL), the mol. sieve was filtered off and washed with chloroform (4 × 5 mL). The filtrate was washed with 0.5 M Na_2_S_2_O_3_ solution (30 mL) and water (50 mL), and concentrated. Two consecutive column chromatographies (first in toluene–EtOAc (10→15%) and then in petroleum ether–EtOAc (15→25%)) resulted in trisaccharide **16** (273 mg, 90%) as a colorless foam, [α]_D_ +75 (c 1, СHCl_3_). ^1^H NMR (600 MHz, CDCl_3_): δ 7.39–7.12 (m, 15 H, Ar), 6.68 (poorly resolved t, 1 H, NHCOCF_3_), 5.49 (br. s, H-2^C^), 5.08 (s, 1 H, H-1^B^), 5.06 (br. s, 1 H, H-1^C^), 4.72–4.68 (m, 3 H, H-1^A^, PhC*Ha*Hb, PhC*Ha*Hb′), 4.57 (d, 1 H, *J* = 10.8 Hz, PhCHa*Hb*), 4.50 (d, 1 H, *J* = 11.0 Hz, PhCHa*Hb*′), 4.24 (d, 1 H, *J* = 11.8 Hz, PhC*Ha*Hb′′), 4.15 (d, 1 H, *J* = 11.8 Hz, PhCHa*Hb*′′), 3.99 (br. s, 1 H, H-2^A^), 3.90 (dd, 1 H, *J*_2,3_ = 3.0 Hz, *J*_3,4_ = 9.8 Hz, H-3^B^), 3.82–3.78 (m, 2 H, H-3^C^, OC*Ha*HbCH_2_CH_2_N), 3.72–3.68 (m, 2 H, C-2^B^, C-3^A^), 3.61 (t, 1 H, *J* = 10.0 Hz, H-4^B^), 3.58–3.43 (m, 6 H, H-5^A^, H-5^B^, H-5^C^, OCHa*Hb*CH_2_C*H*_*2*_N), 3,41 (t, 1 H, *J* = 9.8 Hz, H-4^C^), 3.38 (t, 1 H, *J* = 10.0, H-4^A^), 2.14 (s, 3 H, CH_3_CO), 1.93–1.85 (m, 2 H, OCH_2_C*H*_*2*_CH_2_N), 1.35 (d, 3 H, *J*_6,5_ = 6.2 Hz, H-6^B^), 1.34 (d, 3 H, *J*_6,5_ = 6.3 Hz, H-6^A^), 1.29 (d, 3 H, *J*_6,5_ = 6.1 Hz, H-6^C^). ^13^C NMR (150 MHz, CDCl_3_): δ 170.0 (CH_3_ *C*O), 137.3, 137.2, 137.1, 128.5, 128.4, 128.3, 128.2, 128.0, 127.9, 127.8, 127.4, 99.6 (C-1^C^), 99.1 (C-1^A^), 98.2 (C-1^B^), 78.4 (C-3^A^), 78.3 (C-3^B^), 76.2 (C-2^B^), 75.8 (C-3^C^), 72.9 (C-2^A^), 72.5, 71.7, 71.5 (3 Ph*C*H_2_), 68.0 (C-5^B^), 67.7 (C-5^C^), 67.5 (C-5^A^), 67.3 (C-2^C^), 65.9 (O*C*H_2_CH_2_CH_2_N), 64.4 (C-4^A^), 64.1 (C-4^B^), 63.7 (C-4^C^), 38.1 (OCH_2_CH_2_ *C*H_2_N), 28.3 (OCH_2_ *C*H_2_CH_2_N), 20.9 (*C*H_3_CO), 18.6, 18.5 (×2) (C-6^A^, C-6^B^, C-6^C^). HRMS (ESI): calcd. for C_46_H_55_F_3_N_10_O_12_ [M + NH_4_]^+^ *m/z* 1014.4291; found *m/z* 1014.4291.

**3-Trifluoroacetamidopropyl 4-azido-3-*O*-benzyl-4,6-dideoxy-α-d-mannopyranosyl-(1→3)-4-azido-2-*O*-benzyl-4,6-dideoxy-α-d-mannopyranosyl-(1→2)-4-azido-3-*O*-benzyl-4,6-dideoxy-α-d-mannopyranoside (17)**. 1 M sodium methoxide in MeOH (60 μL) was added to a solution of monoacetate **16** (273 mg, 0.337 mmol) in MeOH (6 mL), and the mixture was stirred for 4 h at room temperature. The solution was made neutral by adding Amberlite IR-120 (H^+^), the resin was filtered off and washed with MeOH (4 × 4 mL). The filtrate was concentrated, and the residue was purified by column chromatography (toluene–EtOAc, 15→25%) to produce deacetylated trisaccharide **17** (250 mg, 96%) as a colorless foam, [α]_D_ +57 (c 1, СHCl_3_). ^1^H NMR (600 MHz, CDCl_3_): δ 7.42–7.13 (m, 15 H, Ar), 6.70 (poorly resolved t, 1 H, NHCOCF_3_), 5.11 (s, 1 H, H-1^C^), 5.10 (s, 1 H, H-1^B^), 4.74–4.65 (m, 4 H, H-1^A^, PhC*H*_*2*_, PhC*Ha*Hb′), 4.58 (d, 1 H, *J* = 10.8 Hz, PhCHa*Hb*′), 4.23 (d, 1 H, *J* = 11.8 Hz, PhC*Ha*Hb′′), 4.17 (br. s, 1 H, H-2^C^), 4.13 (d, 1 H, *J* = 11.8 Hz, PhCHa*Hb*′′), 4.00 (br. s, 1 H, H-2^A^), 3.92 (dd, 1 H, *J*_2,3_ = 2.8 Hz, *J*_3,4_ = 9.6 Hz, H-3^B^), 3.82–3.78 (m, 1 H, OC*Ha*HbCH_2_CH_2_N), 3.72-3.69 (m, 3 H, H-2^B^, H-3^A^, H-3^C^), 3.60 (t, 1 H, *J* = 9.8 Hz, H-4^B^), 3.58–3.44 (m, 6 H, H-5^A^, H-5^B^, H-5^C^, OCHa*Hb*CH_2_C*H*_*2*_N), 3.43 (t, 1 H, *J* = 10.0 Hz, H-4^C^), 3.39 (t, 1 H, *J* = 9.8 Hz, H-4^A^), 2.50 (s, 1 H, OH), 1.94-1.85 (m, 2 H, OCH_2_C*H*_*2*_CH_2_N), 1.36–1.33 (m, 6 H, H-6^A^, H-6^B^), 1.20 (d, 3 H, *J*_6,5_ = 6.1 Hz, H-6^C^). ^13^C NMR (150 MHz, CDCl_3_): δ 137.4, 137.2, 128.6, 128.5, 128.3, 128.2, 128.1, 128.0, 127.8, 127.4 (Ar), 101.2 (C-1^C^), 99.1 (C-1^A^), 98.2 (C-1^B^), 78.5, 78.1 (×2) (C-3^A^, C-3^B^, C-3^C^), 76.4 (C-2^A^), 72.6, 71.9, 71.7 (3 Ph*C*H_2_), 68.0 (C-5^B^), 67.5 (C-5^A^), 67.4 (C-5^C^), 67.2 (C-2^C^), 65.9 (O*C*H_2_CH_2_CH_2_N), 64.5 (C-4^A^), 64.2 (C-4^B^), 63.6 (C-4^C^), 38.1 (OCH_2_CH_2_ *C*H_2_N), 28.3 (OCH_2_ *C*H_2_CH_2_N), 18.6, 18.5 (C-6^A^, C-6^B^), 18.4 (C-6^C^). HRMS (ESI): calcd. for C_44_H_53_F_3_N_10_O_11_ [M + NH_4_]^+^ *m/z* 972.4186; found *m/z* 972.4195.

**3-Trifluoroacetamidopropyl 2-*O*-acetyl-4-azido-3-*O*-benzyl-4,6-dideoxy-α-d-mannopyranosyl-(1→2)-4-azido-3-*O*-benzyl-4,6-dideoxy-α-d-mannopyranosyl-(1→3)-4-azido-2-*O*-benzyl-4,6-dideoxy-α-d-mannopyranosyl-(1→2)-4-azido-3-*O*-benzyl-4,6-dideoxy-α-d-mannopyranoside (18)**. A mixture of imidate **14** (53 mg, 0.113 mmol), acceptor **17** (90 mg, 0.094 mmol) and mol. sieve 4 Å (200 mg) in DCM (2 mL) was stirred for 1 h at room temperature and cooled to −40°C. TMSOTf (4.2 μL, 23 μmol) was added, and the mixture was stirred for 1.5 h, while the temperature was gradually increased to −10°C. After adding Et_3_N (20 μL), the mixture was diluted with chloroform (10 mL), the solids were filtered off and washed with chloroform (4 × 4 mL). The filtrate was washed with 1 M HCl (10 mL), water (30 mL), and the solvent was evaporated. The residue was purified by column chromatography (petroleum ether–EtOAc, 20→25%) to give tetrasaccharide **18** (116 mg, 98%) as a colorless foam, [α]_D_ +59 (c 1, СHCl_3_). ^1^H NMR (600 MHz, CDCl_3_): δ 7.40–7.25 (m, 20 H, Ar), 6.64 (poorly resolved t, 1 H, NHCOCF_3_), 5.43 (br. s, 1 H, H-2^D^), 5.09 (s, 1 H, H-1^B^), 5.00 (s, 1 H, H-1^C^), 4.90 (s, 1 H, H-1^D^), 4.75-4.69 (m, 3 H, H-1^A^, PhC*Ha*Hb, PhC*Ha*Hb′), 4.65 (d, 1 H, *J* = 11.6 Hz, PhC*Ha*Hb′′), 4.62 (d, 1 H, *J* = 11.6 Hz, PhCHa*Hb*′′), 4.57 (d, 1 H, *J* = 11.2 Hz, PhCHa*Hb*), 4.55 (d, 1 H, *J* = 11.8 Hz, PhCHa*Hb*′), 4.25 (d, 1 H, *J* = 12.0 Hz, PhC*Ha*Hb′′′), 4.15(d, 1 H, *J* = 12.0 Hz, PhCHa*Hb*′′′), 4.05 (br. s, 1 H, H-2^C^), 4.00 (br. s, 1 H, H-2^A^), 3.85 (dd, 1 H, *J*_2,3_ = 3.0 Hz, *J*_3,4_ = 9.4 Hz, H-3^B^), 3.82–3.78 (m, 2 H, H-3^D^, OC*Ha*HbCH_2_CH_2_N), 3.71 (dd, 1 H, *J*_2,3_ = 3.0 Hz, *J*_3,4_ = 9.6 Hz, H-3^A^), 3.69 (dd, 1 H, *J*_2,3_ = 3.0 Hz, *J*_3,4_ = 9.6 Hz, H-3^C^), 3.64–3.59 (m, 2 H, H-2^B^, H-5^D^), 3.59–3.52 (m, 2 H, H-4^B^), H-5^B^), 3.50-3.38 (m, 6 H, H-4^D^, H-5^A^, H-5^C^, OCHa*Hb*CH_2_C*H*_*2*_N), 3.36 (t, 1 H, *J* = 10.0 Hz, H-4^A^), 3.35 (t, 1 H, *J* = 10.0 Hz, H-4^C^), 2.09 (s, 3 H, CH_3_CO), 1.93-1.85 (m, 2 H, OCH_2_C*H*_*2*_CH_2_N), 1.35–1.32 (m, 9 H, H-6^A^, H-6^B^, H-6^D^), 1.18 (d, 3 H, *J*_6,5_ = 6.1 Hz, H-6^C^). ^13^C NMR (150 MHz, CDCl_3_): δ 169.7 (CH_3_ *C*O), 137.4, 137.3, 137.1, 128.6, 128.5, 128.4, 128.2, 128.0, 127.8, 127.5 (Ar), 100.7 (C-1^C^), 99.4 (C-1^D^), 99.1 (C-1^A^), 98.3 (C-1^B^), 78.4 (C-3^A^), 77.5 (C-3^C^), 77.3 (C-3^B^), 76.3 (C-2^B^), 75.5 (C-3^D^), 73.3 (C-2^C^), 72.8 (C-2^A^), 72.5, 71.9, 71.8, 71.6 (4 Ph*C*H_2_), 68.0, (C-5^B^), 67.8 (C-5^C^), 67.7 (C-5^D^), 67.5 (C-5^A^), 67.1 (C-2^D^), 65.9 (O*C*H_2_CH_2_CH_2_N), 64.5 (C-4^B^), 64.4 (C-4^A^), 63.8 (C-4^D^), 63.7 (C-4^C^), 38.1 (OCH_2_CH_2_ *C*H_2_N), 28.4 (OCH_2_ *C*H_2_CH_2_N), 20.9 (*C*H_3_CO), 18.5 (×2), 18.4 (×2) (C-6^A^, C-6^B^, C-6^C^, C-6^D^). HRMS (ESI): calcd. for C_59_H_70_F_3_N_13_O_15_ [M + NH_4_]^+^ *m/z* 1275.5405; found *m/z* 1275.5399.

**3-Trifluoroacetamidopropyl 4-azido-3-*O*-benzyl-4,6-dideoxy-α-d-mannopyranosyl-(1→2)-4-azido-3-*O*-benzyl-4,6-dideoxy-α-d-mannopyranosyl-(1→3)-4-azido-2-*O*-benzyl-4,6-dideoxy-α-d-mannopyranosyl-(1→2)-4-azido-3-*O*-benzyl-4,6-dideoxy-α-d-mannopyranoside (19)**. 1 M sodium methoxide (50 μL) was added to a solution of compound **18** (298 mg, 0.237 mmol) in MeOH (5 mL), and the mixture was stirred for 4.5 h at room temperature. The solution was made neutral by adding Amberlite IR-120 (H^+^), the resin was filtered off and washed with MeOH (4 × 4 mL). The filtrate was evaporated, and the residue was subjected to column chromatography (toluene–EtOAc, 10→25%) to produce deacetylated tetrasaccharide **19** (274 mg, 95%) as a colorless foam, [α]_D_ +73 (c 1, СHCl_3_). ^1^H NMR (600 MHz, CDCl_3_): δ 7.43–7.26 (m, 20 H, Ar), 6.65 (br. s, 1 H, NHCOCF_3_), 5.09 (s, 1 H, H-1^B^), 5.03 (s, 1 H, H-1^C^), 5.00 (s, 1 H, H-1^D^), 4.74 (d, 1 H, *J* = 11.2 Hz, PhC*Ha*Hb), 4.72 (s, 1 H, H-1^A^), 4.71 (d, 1 H, *J* = 10.8 Hz, PhC*Ha*Hb′), 4.69 (d, 1 H, *J* = 11.2 Hz, PhCHa*Hb*), 4.63 (s, 2 H, PhC*H*_*2*_″), 4.68 (d, 1 H, *J* = 10.8 Hz, PhCHa*Hb*′), 4.25 (d, 1 H, *J* = 12.0 Hz, PhC*Ha*Hb‴), 4.16 (d, 1 H, *J* = 12.0 Hz, PhCHa*Hb*‴), 4.09 (br. s, 1 H, H-2^C^), 4.00 (br. s, 2 H, H-2^A^, H-2^D^), 3.86 (dd, 1 H, *J*_2,3_ = 3.0 Hz, *J*_3,4_ = 9.5 Hz, H-3^B^), 3.83–3.78 (m, 1 H, OC*Ha*HbCH_2_CH_2_N), 3.75–3.69 (m, 3 H, H-3^A^, H-3^C^, H-3^D^), 3.66–3.60 (m, 2 H, H-2^B^, H-5^D^), 3.60–3.52 (m, 3 H, H-4^B^, H-5^B^, OCHa*Hb*CH_2_C*H*_*2*_N), 3.51-3.39 (m, 5 H, H-4^D^, H-5^A^, H-5^С^, OCH_2_CH_2_C*H*_*2*_N), 3.37 (t, 1 H, *J* = 10.0 Hz, H-4^A^), 3.32 (t, 1 H, *J* = 9.8 Hz, H-4^C^), 2.32 (br. s, 1 H, OH), 1.94-1.86 (m, 2 H, OCH_2_C*H*_*2*_CH_2_N), 1.36–1.33 (m, 9 H, H-6^A^, H-6^B^, H-6^D^), 1.19 (d, 3 H, *J*_6,5_ = 6.1 Hz, H-6^C^). ^13^C NMR (150 MHz, CDCl_3_): δ 137.4, 137.1, 128.6, 128.5, 128.4, 128.3, 128.2, 128.0, 127.8, 127.5 (Ar), 100.8 (C-1^C^), 100.7 (C-1^D^), 99.1 (C-1^A^), 98.4 (C-1^B^), 78.4 (C-3^A^), 77.7, 77.6 (C-3^C^, C-3^D^), 77.4 (C-3^B^), 76.3 (C-2^B^), 73.3 (C-2^C^), 72.8 (C-2^A^), 72.5, 72.1, 72.0, 71.8 (Ph*C*H_2_), 68.1 (C-5^B^), 67.9 (C-5^C^), 67.5 (C-5^A^), 67.4 (C-5^D^), 67.1 (C-2^D^), 65.9 (O*C*H_2_CH_2_CH_2_N), 64.5 (×2) (C-4^A^, C-4^B^), 63.9 (C-4^С^), 63.8 (C-4^D^), 38.1 (OCH_2_CH_2_ *C*H_2_N), 28.4 (OCH_2_ *C*H_2_CH_2_N), 18.5 (×3), 18.4 (C-6^A^, C-6^B^, C-6^C^, C-6^D^). HRMS (ESI): calcd. for C_57_H_68_F_3_N_13_O_14_ [M + Na]^+^ *m/z* 1238.4853; found *m/z* 1238.4854.

**3-Trifluoroacetamidopropyl 3-*O*-acetyl-4-azido-3-*O*-benzyl-4,6-dideoxy-α-d-mannopyranosyl-(1→3)-4-azido-2-*O*-benzyl-4,6-dideoxy-α-d-mannopyranoside (20)**. A mixture of thiglycoside **15** (220 mg, 0.295 mmol), alcohol **11** (76 mg, 0.444 mmol) and mol. sieve 4 Å in DCM (5 mL) was stirred for 1 h at room temperature and then cooled to −45°C. NIS (132 mg, 0.587 mmol) and TMSOTf (22 μL, 0.121 mmol) were added, and the mixture was stirred for 1 h with gradual increase of the temperature to −10°C. Pyridine (50 μL) was added, the mixture was diluted with chloroform (10 mL), the solids were filtered off and washed with chloroform (4 × 4 mL). The filtrate was washed with 0.5 M Na_2_S_2_O_3_ solution (30 mL) and water (50 mL), and concentrated. Column chromatography of the residue (petroleum ether–EtOAc, 20→30%) produced glycoside **20** (194 mg, 89%) as a colorless syrup, [α]_D_ +83 (c 1, СHCl_3_). ^1^H NMR (600 MHz, CDCl_3_): δ 7.39–7.27 (m, 10 H, Ar), 6.57 (br. s, 1 H, NHCOCF_3_), 5.48 (br, s, 1 H, H-2^B^), 5.04 (s, 1 H, H-1^B^), 4.78 (s, 1 H, H-1^A^), 4.70 (d, 1 H, *J* = 11.2 Hz, PhC*Ha*Hb), 4.65 (d, 1 H, *J* = 11.8 Hz, PhC*Ha*Hb′), 4.60 (d, 1 H, *J* = 11.8 Hz, PhCHa*Hb*′), 4.53 (d, 1 H, *J* = 11.2 Hz, PhCHa*Hb*), 3.86 (dd, 1 H, *J*_2,3_ = 3.2 Hz, *J*_3,4_ = 9.8 Hz, H-3^B^), 3.83 (dd, 1 H, *J*_2,3_ = 3.2 Hz, *J*_3,4_ = 10.2 Hz, H-3^A^), 3.78-3.74 (m, 1 H, OC*Ha*HbCH_2_CH_2_N), 3.71 (br. s, 1 H, H-2^A^), 3.69 (dq, 1 H, *J*_4,5_ = 10.0 Hz, *J*_5,6_ = 6.1 Hz, H-5^B^), 3.62 (t, 1 H, *J* = 10.0 Hz, H-4^A^), 3.53–3.38 (m, 5 H, H-4^B^, H-5^A^, OCHa*Hb*CH_2_C*H*_*2*_N), 2.14 (s, 3 H, CH_3_CO), 1.88–1.83 (m, 2 H, OCH_2_C*H*_*2*_CH_2_N), 1.35 (d, 3 H, *J*_6,5_ = 6.1 Hz, H-6^A^), 1.27 (d, 3 H, *J*_6,5_ = 6.1 Hz, H-6^B^). ^13^C NMR (150 MHz, CDCl_3_): δ 170.1 (CH_3_ *C*O), 137.5, 137.2, 128.5, 128.4, 128.3, 128.0, 127.9, 127.5 (Ar), 99.7 (C-1^B^), 97.2 (C-1^A^), 79.2 (C-3^A^), 76.5 (C-2^A^), 75.8 (C-3^B^), 72.6, 71.5 (2 Ph*C*H_2_), 67.9 (C-5^B^), 67.6 (C-5^A^), 67.4 (C-2^B^), 65.7 (O*C*H_2_CH_2_CH_2_N), 64.2 (C-4^A^), 63.8 (C-4^B^), 37.9 (OCH_2_CH_2_ *C*H_2_N), 28.4 (OCH_2_ *C*H_2_CH_2_N), 20.9 (*C*H_3_CO), 18.6 (C-6^B^), 18.5 (C-6^A^). HRMS (ESI): calcd. for C_33_H_40_F_3_N_7_O_9_ [M + Na]^+^ *m/z* 758.2732; found *m/z* 758.2732.

**3-Trifluoroacetamidopropyl 4-azido-3-*O*-benzyl-4,6-dideoxy-α-d-mannopyranosyl-(1→3)-4-azido-2-*O*-benzyl-4,6-dideoxy-α-d-mannopyranoside (21)**. 1 M sodium methoxide (25 μL) was added to a solution of compound 20 (77 mg, 0.105 mmol) in MeOH (2.5 mL), and the mixture was stirred for 3 h at room temperature. The solution was made neutral by adding Amberlite IR-120 (H^+^), the resin was filtered off and washed with MeOH (4 × 4 mL). The filtrate was evaporated, and the residue was subjected to column chromatography (toluene–EtOAc, 15→25%) to give deacetylated product **21** (70 mg, 96%) as a colorless syrup, [α]_D_ +89 (c 1, СHCl_3_). ^1^H NMR (600 MHz, CDCl_3_): δ 7.43–7.32 (m, 10 H, Ar), 6.60 (br. s, 1 H, NHCOCF_3_), 5.09 (s, 1 H, H-1^B^), 4.80 (s, 1 H, H-1^A^), 4.70 (d, 1 H, *J* = 11.6 Hz, PhC*Ha*Hb), 4.68 (d, 1 H, *J* = 11.6 Hz, PhCHa*Hb*), 4.67 (d, 1 H, *J* = 11.6 Hz, PhC*Ha*Hb′), 4.60 (d, 1 H, *J* = 11.6 Hz, PhCHa*Hb*′), 4.19 (br. s, 1 H, H-2^B^), 3.84 (dd, 1 H, *J*_2,3_ = 3.2 Hz, *J*_3,4_ = 10.0 Hz, H-3^A^), 3.79–3.74 (m, 2 H, H-3^B^, OC*Ha*HbCH_2_CH_2_N), 3.72 (br. s, 1 H, H-2^A^), 3.68 (dq, 1 H, *J*_4,5_ = 10.0 Hz, *J*_5,6_ = 6.3 Hz, H-5^B^), 3.60 (t, 1 H, *J* = 10.2 Hz, H-4^A^), 3.52–3.40 (m, 5 H, H-4^B^, H-5^A^, OCHa*Hb*CH_2_C*H*_*2*_N), 2.49 (s, 1 H, OH), 1.89–1.84 (m, 2 H, OCH_2_C*H*_*2*_CH_2_N), 1.35 (d, 3 H, *J*_6,5_ = 6.3 Hz, H-6^A^), 1.28 (d, 3 H, *J*_6,5_ = 6.3 Hz, H-6^B^). ^13^C NMR (150 MHz, CDCl_3_): δ 137.5, 137.2, 128.6, 128.5, 128.2, 128.1, 128.0, 127.5 (Ar), 101.4 (C-1^B^), 97.2 (C-1^A^), 78.9 (C-3^A^), 78.0 (C-3^B^), 76.8 (C-2^A^), 72.6, 71.9 (2 Ph*C*H_2_), 67.5 (×2) (C-5^A^, C-5^B^), 67.2 (C-2^B^), 65.7 (O*C*H_2_CH_2_CH_2_N), 64.3 (C-4^A^), 63.7 (C-4^B^), 37.9 (OCH_2_CH_2_ *C*H_2_N), 28.4 (OCH_2_ *C*H_2_CH_2_N), 18.5 (×2) (C-6^A^, C-6^B^). HRMS (ESI): calcd. for C_31_H_38_F_3_N_7_O_8_ [M + Na]^+^ *m/z* 716.2626; found *m/z* 716.2628.

**3-Trifluoroacetamidopropyl 2-*O*-acetyl-4-azido-3-*O*-benzyl-4,6-dideoxy-α-d-mannopyranosyl-(1→2)-4-azido-3-*O*-benzyl-4,6-dideoxy-α-d-mannopyranosyl-(1→3)-4-azido-2-*O*-benzyl-4,6-dideoxy-α-d-mannopyranoside (22)**. Mol. sieve 4 Å (500 mg) was added to a solution of imidate **14** (150 mg, 0.32 mmol) and acceptor **21** (195 mg, 0.28 mmol) in DCM (5 mL), the resulting mixture was stirred for 1 h at room temperature, and then cooled to −40°C. TMSOTf (11.6 μL, 0.064 mmol) was added, and the mixture was stirred for 1 h with gradual increasing the temperature to −10°C, after which the reaction was quenched with Et_3_N (20 μL). The mixture was diluted with chloroform (10 mL), and the solids were filtered off and washed with chloroform (4 × 4 mL). The filtrate was washed with 1 M HCl (20 mL) and water (30 mL), and the solvent was evaporated. The residue was purified by column chromatography (petroleum ether–EtOAc, 20→30%) to yield trisaccharide **22** (260 mg, 93%) as a colorless foam, [α]_D_ +70 (c 1, СHCl_3_). ^1^H NMR (600 MHz, CDCl_3_): δ 7.40–7.27 (m, 15 H, Ar), 6.55 (poorly resolved t, 1 H, NHCOCF_3_), 5.44 (br. s, 1 H, H-2^C^), 5.00 (s, 1 H, H-1^B^), 4.92 (s, 1 H, H-1^C^), 4.79 (s, 1 H, H-1^A^), 4.74 (d, 1 H, *J* = 11.0 Hz, PhC*Ha*Hb), 4.68 (d, 1 H, *J* = 11.6 Hz, PhC*Ha*Hb′), 4.65 (d, 1 H, *J* = 11.8 Hz, PhC*Ha*Hb′′), 4.64 (d, 1 H, *J* = 11.6 Hz, PhCHa*Hb*′), 4.60 (d, 1 H, *J* = 11.8 Hz, PhCHa*Hb*′′), 4.55 (d, 1 H, *J* = 11.0 Hz, PhCHa*Hb*), 4.07 (br. s, 1 H, H-2^B^), 3.81–3.74 (m, 4 H, H-3^A^, H-3^B^, H-3^C^, OC*Ha*HbCH_2_CH_2_N), 3.65 (br. s, 1 H, H-2^A^), 3.62–3.54 (m, 3 H, H-4^A^, H-5^B^, H-5^C^), 3.53–3.40 (m, 5 H, H-4^C^, H-5^A^, OCHa*Hb*CH_2_C*H*_*2*_N), 3.38 (t, 1 H, *J* = 9.8 Hz, H-4^B^), 2.10 (s, 3 H, CH_3_CO), 1.89–1.84 (m, 2 H, OCH_2_C*H*_*2*_CH_2_N), 1.35 (d, 3 H, *J*_6,5_ = 6.3 Hz, H-6^A^), 1.33 (d, 3 H, *J*_6,5_ = 6.1 Hz, H-6^C^), 1.26 (d, 3 H, *J*_6,5_ = 6.1 Hz, H-6^B^). ^13^C NMR (150 MHz, CDCl_3_): δ 169.8 (CH_3_ *C*O), 137.4, 137.1, 128.6, 128.5, 128.4, 128.0, 127.9, 127.6 (Ar), 101.0 (C-1^B^), 99.3 (C-1^C^), 97.4 (C-1^A^), 78.2 (C-3^A^), 77.4 (C-3^B^), 76.7 (C-2^A^), 75.4 (C-3^C^), 73.2 (C-2^B^), 72.7, 71.9, 71.6 (3 Ph*C*H_2_), 68.0 (C-5^B^), 67.7 (C-5^C^), 67.6 (C-5^A^), 67.1 (C-2^C^), 65.7 (O*C*H_2_CH_2_CH_2_N), 64.7 (C-4^A^), 63.8 (×2) (C-4^B^, C-4^C^), 37.8 (OCH_2_CH_2_ *C*H_2_N), 28.4 (OCH_2_ *C*H_2_CH_2_N), 18.6, 18.5, 18.4 (C-6^A^, C-6^B^, C-6^C^). HRMS (ESI): calcd. for C_46_H_55_F_3_N_10_O_12_ [M + Na]^+^ *m/z* 1019.3845; found *m/z* 1019.3846.

**3-Trifluoroacetamidopropyl 4-azido-3-*O*-benzyl-4,6-dideoxy-α-d-mannopyranosyl-(1→2)-4-azido-3-*O*-benzyl-4,6-dideoxy-α-d-mannopyranosyl-(1→3)-4-azido-2-*O*-benzyl-4,6-dideoxy-α-d-mannopyranoside (23)**. 1 M sodium methoxide (70 μL) was added to a solution of compound **22** (347 mg, 0.348 mmol) in MeOH (7 mL). After being stirred for 4 h at room temperature, the mixture was made neutral with Amberlite IR-120 (H^+^) and the resin was filtered off and washed with MeOH (4 × 4 mL). The filtrate was concentrated, and the residue was purified by column chromatography (toluene–EtOAc, 10→20%) to afford deacetylated trisaccharide **23** (306 mg, 92%) as a colorless foam, [α]_D_ +86 (c 1, СHCl_3_). ^1^H NMR (600 MHz, CDCl_3_): δ 7.43-7.30 (m, 15 H, Ar), 6.58 (poorly resolved t, 1 H, NHCOCF_3_), 5.03 (s, 1 H, H-1^B^), 5.01 (s, 1 H, H-1^C^), 4.79 (s, 1 H, H-1^A^), 4.74 (d, 1 H, *J* = 11.2 Hz, PhC*Ha*Hb), 4.70 (d, 1 H, *J* = 11.2 Hz, PhCHa*Hb*), 4.65 (s, 2 H, PhC*H*_*2*_′), 4.65 (d, 1 H, *J* = 11.8 Hz, PhC*Ha*Hb′′), 4.61 (d, 1 H, *J* = 11.8 Hz, PhCHa*Hb*′′), 4.12 (br. s, 1 H, H-2^B^), 4.01 (s, 1 H, H-2^C^), 3.81–3.74 (m, 3 H, H-3^A^, H-3^B^, OC*Ha*HbCH_2_CH_2_N), 3.73 (dd, 1 H, *J*_2,3_ = 3.2 Hz, *J*_3,4_ = 9.8 Hz, H-3^C^), 3.66 (br. s, 1 H, H-2^A^), 3.64–3.55 (m, 3 H, H-4^A^, H-5^B^, H-5^C^), 3.54–3.40 (m, 5 H, H-4^C^, H-5^A^, OCHa*Hb*CH_2_C*H*_*2*_N), 3.36 (t, 1 H, *J* = 9.8 Hz, H-4^B^), 2.35 (br. s, 1 H, OH), 1.90–1.84 (m, 2 H, OCH_2_C*H*_*2*_CH_2_N), 1.36 (d, 3 H, *J*_6,5_ = 6.1 Hz, H-6^A^), 1.34 (d, 3 H, *J*_6,5_ = 6.1 Hz, H-6^C^), 1.27 (d, 3 H, *J*_6,5_ = 6.3 Hz, H-6^B^). ^13^C NMR (150 MHz, CDCl_3_): δ 137.4, 137.3, 137.1, 128.6, 128.5, 128.3, 128.2, 128.0, 127.6 (Ar), 101.1 (C-1^B^), 100.7 (C-1^C^), 97.4 (C-1^A^), 78.2 (C-3^A^), 77.6 (C-3^C^), 77.5 (C-3^B^), 76.7 (C-2^A^), 73.2 (C-2^B^), 72.7, 72.0 (×2) (3 Ph*C*H_2_), 67.9 (C-5^B^), 67.6 (C-5^A^), 67.4 (C-5^C^), 67.1 (C-2^C^), 65.7 (O*C*H_2_CH_2_CH_2_N), 64.7 (C-4^A^), 64.0 (C-4^B^), 63.8 (C-4^C^), 37.9 (OCH_2_CH_2_ *C*H_2_N), 28.4 (OCH_2_ *C*H_2_CH_2_N), 18.6 (C-6^B^), 18.5 (C-6^A^), 18.3 (C-6^C^). HRMS (ESI): calcd. for C_44_H_53_F_3_N_10_O_11_ [M + NH_4_]^+^ *m/z* 972.4186; found *m/z* 972.4170.

**5-(*tert*-Butyl)-2-methylphenyl 2-*O*-acetyl-3,4-di-*O*-benzyl-1-thio-α,β-** **d** **-rhamnopyranoside (27).** BF_3_·Et_2_O (68 μL, 0.55 mmol) was added to a chilled (ice bath) solution of 1,2-diacetate **27** (195 mg, 0.455 mmol) and 5-(*tert*-butyl)-2-methylthiophenol in DCM (4 mL). The mixture was stirred for 15 min., then the cooling bath was removed and stirring was continued at room temperature for next 2 h. The mixture was diluted with chloroform (30 mL), washed with water (30 mL) and saturated NaHCO_3_ solution, and concentrated. Column chromatography of the residue (petroleum ether–EtOAc, 0→10%) produced an anomeric mixture (α:β ratio ∼1.3:1) of thiglycosides **27** (219 mg, 88%) as a colorless syrup. ^1^H NMR (600 MHz, CDCl_3_): δ 7.67–7.13 (m, 30 H, Ar), 5.84 (d, 1H, *J*_2,3_ = 3.2 Hz, H-2_β_), 5.68 (br. s, 1.3 H, H-2_α_), 5.37 (s, 1.3 H, H-1_α_), 4.97 (d, 1.3 H, *J* = 11.0 Hz, PhC*Ha*Hb_α_), 4.95 (d, 1 H, *J* = 11.0 Hz, PhC*Ha*Hb_β_), 4.84 (d, 1 H, *J* = 11.0 Hz, PhC*Ha*Hb_α_′), 4.81 (s, 1 H, H-1_β_), 4.77 (d, 1.3 H, *J* = 11.2 Hz, PhC*Ha*Hb_α_′), 4.67 (d, 1.3 H, *J* = 11.0 Hz, PhCHa*Hb*_α_), 4.65 (d, 1 H, *J* = 11.0 Hz, PhCHa*Hb*_β_), 4.61 (d, 1.3 H, *J* = 11.2 Hz, PhCHa*Hb*_α_′), 4.53 (d, 1 H, *J* = 11.0 Hz, PhCHa*Hb*_β_′), 4.32 (dq, 1.3 H, *J*_4,5_ = 9.4 Hz, H-5_α_), 4.00 (dd, 1.3 H, *J*_2,3_ = 3.2 Hz, *J*_3,4_ = 9.4 Hz, H-3_α_), 3.68 (dd, 1 H, *J*_3,4_ = 8.7 Hz, H-3_β_), 3.55 (t, 1.3 H, *J* = 9.4 Hz, H-4_α_), 3.51 (t, 1 H, *J* = 9.2 Hz, H-4_β_), 3.49 (dq, 1 H, *J*_4,5_ = 9.2 Hz, H-5_β_), 2.42 (s, 4 H, C*H*_*3*_-Ar_α_), 2.39 (s, 3 H, C*H*_*3*_-Ar_β_), 2.28 (s, 3 H, CH_3_CO_β_), 2.18 (s, 4 H, CH_3_CO_α_), 1.49 (d, 3 H, *J*_5,6_ = 5.7 Hz, H-6_β_), 1.38 (d, 3 H, *J*_5,6_ = 6.1 Hz, H-6_α_), 1.33 (s, 21 H, C(CH_3_)_3_). ). ^13^C NMR (150 MHz, CDCl_3_): δ 170.4, 170.3 (CH_3_ *C*O), 149.7, 149.6, 138.4, 138.2, 137.7, 137.6, 136.7, 135.8, 133.2, 132.6, 130.0, 129.8, 128.4, 128.3, 128.1, 128.0, 127.8, 127.7, 125.0, 124.5 (Ar), 85.8 (C-1_α_), 84.8 (C-1_β_), 81.3 (C-3_β_), 80.2 (C-4_α_), 79.3 (C-4_β_), 78.5 (C-3_α_), 76.1 (C-5_β_), 75.5, 71.9, 71.8 (Ph*C*H_2_), 70.9 (C-2_α_), 70.7 (C-2_β_), 69.1 (C-5_α_), 31.3 (C(*C*H_3_)_3_), 21.1 (*C*H_3_CO_α_), 20.9 (*C*H_3_CO_β_), 20.2 (*C*H_3_-Ar), 18.3 (C-6_β_), 17.9 (C-6_α_). HRMS (ESI): calcd. for C_33_H_40_O_5_S [M + Na]^+^ *m/z* 571.2489; found *m/z* 571.2482.

**3-Trifluoroacetamidopropyl 2-*O*-acetyl-3,4-di-*O*-benzyl-α-d-rhamnopyranosyl-(1→2)-4-azido-3-*O*-benzyl-4,6-dideoxy-α-d-mannopyranosyl-(1→2)-4-azido-3-*O*-benzyl-4,6-dideoxy-α-d-mannopyranosyl-(1→3)-4-azido-2-*O*-benzyl-4,6-dideoxy-α-d-mannopyranosyl-(1→2)-4-azido-3-*O*-benzyl-4,6-dideoxy-α-d-mannopyranoside (28)**. A mixture of thiglycoside **27** (58 mg, 0.106 mmol), acceptor **19** (103 mg, 0.085 mmol) and vol. sieve 4 Å (150 mg) in DCM (3 mL) was for 1 h at room temperature and then cooled to −20°C. NIS (48 mg, 0.212 mmol) and TMSOTf (3.8 μL, 0.021 mmol) were added, and stirring was continued for 1 h at −20°C. The reaction was quenched with pyridine (20 μL), the mixture was diluted with chloroform (10 mL), and the solids were filtered off and washed with chloroform (4 × 4 mL). The filtrate was washed with 0.5 M Na_2_S_2_O_3_ solution (20 mL) and water (30 mL), and concentrated. Column chromatography of the residue (toluene–EtOAc, 8→13%) produced pentasaccharide **28** (121 mg, 90%) as a colorless foam, [α]_D_ +49 (c 1, СHCl_3_). ^1^H NMR (600 MHz, CDCl_3_): δ 7.42–7.22 (m, 30 H, Ar), 6.66 (poorly resolved t, 1 H, NHCOCF_3_), 5.46 (br. s, 1 H, H-2^E^), 5.08 (s, 1 H, H-1^B^), 5.04 (s, 1 H, H-1^D^), 4.99 (s, 1 H, H-1^C^), 4.90 (d, 1 H, *J* = 10.8 Hz, benzylic H), 4.85 (s, 1 H, H-1^E^), 4.76-4.51 (m, 10 H, 9 benzylic H, H-1^A^), 4.23 (d, 1 H, *J* = 11.8 Hz, PhC*Ha*Hb), 4.15 (d, 1 H, *J* = 11.8 Hz, PhCHa*Hb*), 4.03 (br. s, 1 H, H-2^C^), 4.00 (br. s, 1 H, H-2^A^), 3.94–3.90 (m, 2 H, H-2^D^, H-3^E^), 3.85 (dd, 1 H, *J*_3,2_ = 2.8 Hz, *J*_3,4_ = 9.4 Hz, H-3^B^), 3.83–3.78 (m, 1 H, OC*Ha*HbCH_2_CH_2_N), 3.78–3.69 (m, 3 H, H-3^A^, H-3^D^, H-5^E^), 3.66 (dd, 1 H, *J*_3,2_ = 2.8 Hz, *J*_3,4_ = 10.0 Hz, H-3^C^), 3.61 (br. s, 1 H, H-2^B^), 3.59–3.52 (m, 4 H, H-4^B^, H-5^B^, H-5^D^, OCH_2_CH_2_C*Ha*HbN), 3.51–3.32 (m, 7 H, H-4^A^, H-4^D^, H-4^E^, H-5^A^, H-5^C^, OCHa*Hb*CH_2_CH_2_N, OCH_2_CH_2_CHa*Hb*N), 3.25 (t, 1 H, *J* = 9.8 Hz, H-4^C^), 2.14 (s, 3 H, CH_3_CO), 1.93–1.88 (m, 2 H, OCH_2_C*H*_*2*_CH_2_N), 1.36–1.33 (m, 6 H, H-6^A^, H-6^D^), 1.31 (d, 3 H, *J*_6,5_ = 6.1 Hz, H-6^B^), 1.22 (d, 3 H, *J*_6,5_ = 6.3 Hz, H-6^E^), 1.17 (d, 3 H, *J*_6,5_ = 6.3 Hz, H-6^C^). ^13^C NMR (150 MHz, CDCl_3_): δ 169.9 (CH_3_ *C*O), 138.4, 137.0, 137.5, 137.3, 137.1, 128.6, 128.5, 128.4, 128.3, 128.2, 128.1, 128.0, 127.9, 127.8, 127.7, 127.6, 127.5 (Ar), 100.8 (C-1^C^), 100.5 (C-1^D^), 99.3 (С-1^E^), 99.1 (C-1^A^), 98.4 (C-1^B^), 79.9 (C-4^E^), 78.4 (C-3^A^), 77.5 (C-3^E^), 77.2 (C-3^B^), 77.1 (C-3^C^), 76.9 (C-3^D^), 76.2 (C-2^B^), 75.4 (Ph*C*H_2_), 72.8 (C-2^A^), 72.5 (Ph*C*H_2_), 71.9, 71.8 (×2), 71.7 (4 Ph*C*H_2_), 68.8 (C-2^E^), 68.1, 67.9, 67.8 (C-5^B^, C-5^C^, C-5^D^), 67.5 (C-5^A^), 65.9 (O*C*H_2_CH_2_CH_2_N), 64.6 (C-4^B^), 64.4 (C-4^A^), 64.0, 63.9 (C-4^C^, C-4^D^), 38.1 (OCH_2_CH_2_ *C*H_2_N), 28.4 (OCH_2_ *C*H_2_CH_2_N), 21.1 (*C*H_3_CO), 18.0 (×4) (C-6^A^, C-6^B^, C-6^C^, C-6^D^), 17.8 (C-6^E^). HRMS (ESI): calcd. for C_79_H_92_F_3_N_13_O_19_ [M + NH_4_]^+^ *m/z* 1601.6923; found *m/z* 1601.6906.

**3-Trifluoroacetamidopropyl 3,4-di-*O*-benzyl-α-d-rhamnopyranosyl-(1→2)-4-azido-3-*O*-benzyl-4,6-dideoxy-α-d-mannopyranosyl-(1→2)-4-azido-3-*O*-benzyl-4,6-dideoxy-α-d-mannopyranosyl-(1→3)-4-azido-2-*O*-benzyl-4,6-dideoxy-α-d-mannopyranosyl-(1→2)-4-azido-3-*O*-benzyl-4,6-dideoxy-α-d-mannopyranoside (29).** 1 M sodium methoxide (20 μL) was added to a solution of compound **28** (121 mg, 0.076 mmol) in MeOH (2 mL). After being stirred for 4 h at room temperature, the mixture was made neutral with Amberlite IR-120 (H^+^) and the resin was filtered off and washed with MeOH (4 × 4 mL). The filtrate was concentrated, and the residue was purified by column chromatography (toluene–EtOAc, 8→15%) to yield deacetylated pentasaccharide **29** (109 mg, 92%) as a colorless foam, [α]_D_ +59 (c 1, СHCl_3_). ^1^H NMR (600 MHz, CDCl_3_): δ 7.42–7.23 (m, 30 H, Ar), 6.63 (br. s, 1 H, NHCOCF_3_), 5.08 (s, 1 H, H-1^B^), 5.05 (s, 1 H, H-1^D^), 5.00 (s, 1 H, H-1^C^), 4.97 (s, 1 H, H-1^E^), 4.88 (d, 1 H, *J* = 11.0 Hz, benzylic H), 4.75–4.62 (m, 8 H, H-1^A^, 7 benzylic H), 4.57 (d, 1 H, *J* = 11.0 Hz, benzylic H), 4.54 (d, 1 H, *J* = 11.6 Hz, benzylic H), 4.24 (d, 1 H, *J* = 11.8 Hz, PhC*Ha*Hb), 4.17 (d, 1 H, *J* = 11.8 Hz, PhCHa*Hb*), 4.06 (br. s, 1 H, H-2^E^), 4.03 (br. s, 1 H, H-2^C^), 4.00–3.97 (m, 2 H, H-2^A^, H-2^D^), 3.87–3.82 (m, 2 H, H-3^B^, H-3^E^), 3.82–3.77 (m, 1 H, OC*Ha*HbCH_2_CH_2_N), 3.77–3.69 (m, 3 H, H-3^A^, H-3^D^, H-5^E^), 3.67 (dd, 1 H, , *J*_3,2_ = 2.6 Hz, *J*_3,4_ = 9.8 Hz, H-3^C^), 3.61 (br. s, 1 H, H-2^B^), 3.58–3.51 (m, 4 H, H-4^B^, H-5^B^, H-5^D^, OCH_2_CH_2_C*Ha*HbN), 3.51–3.43 (m, 4 H, H-4^E^, H-5^A^, OCHa*Hb*CH_2_CH_2_N, OCH_2_CH_2_CHa*Hb*N), 3.40–3.34 (m, 3 H, H-4^A^, H-4^D^, H-5^C^), 3.27 (t, 1 H, *J* = 9.8 Hz, H-4^C^), 2.40 (br. s, 1 H, OH), 1.94–1.85 (m, 2 H, OCH_2_C*H*_*2*_CH_2_N), 1.36-1.32 (m, 6 H, H-6^A^, H-6^D^), 1.31 (d, 3 H, *J*_6,5_ = 6.1 Hz, H-6^B^), 1.20 (d, 3 H, *J*_6,5_ = 6.1 Hz, H-6^E^), 1.17 (d, 3 H, *J*_6,5_ = 6.1 Hz, H-6^C^). ^13^C NMR (150 MHz, CDCl_3_): δ 138.4, 137.9, 137.4, 137.3, 137.2, 128.6, 128.5, 128.4, 128.3, 128.2, 128.1, 128.0, 127.8, 127.7, 127.5 (Ar), 100.8 (C-1^C^), 100.7 (×2), (C-1^D^, C-1^E^), 99.1 (C-1^A^), 98.5 (C-1^B^), 79.9 (C-4^E^), 79.5 (C-3^E^), 78.4 (C-3^A^), 77.2 (C-3^B^), 77.1 (×2) (C-3^C^, C-3^D^), 76.4 (С-2^B^), 75.4 (Ph*C*H_2_), 73.4 (C-2^C^), 73.2 (C-2^D^), 72.9 (C-2^A^), 72.5, 72.2, 72.1, 71.9, 71.8 (5 Ph*C*H_2_), 68.6 (C-2^E^), 68.1 (×2), 67.9 (×2) (C-5^B^, C-5^C^, C-5^D^, C-5^E^), 67.5 (C-5^A^), 65.9 (O*C*H_2_CH_2_CH_2_N), 64.6 (C-4^B^), 64.5 (C-4^A^), 64.2 (C-4^D^), 64.0 (C-4^C^), 38.1 (OCH_2_CH_2_ *C*H_2_N), 28.4 (OCH_2_ *C*H_2_CH_2_N), 18.5 (×4) (C-6^A^, C-6^B^, C-6^C^, C-6^D^), 17.7 (C-6^E^). HRMS (ESI): calcd. for C_77_H_90_F_3_N_13_O_18_ [M + NH_4_]^+^ *m/z* 1559.6817; found *m/z* 1559.6804.

**3-Aminopropy 4,6-dideoxy-4-formamido-α-** **d** **-mannopyranosyl-(1**→**3)-4,6-dideoxy-4-formamido-α-** **d** **-mannopyranoside (4).** A mixture of diazide **21** (95 mg, 0.137 mmol) and Pd(OH)_2_/C (30 mg) in MeOH was stirred in a hydrogen atmosphere at 35°C for 1.5 h. The catalyst was filtered off and washed with MeOH (4 × 4 mL), and the filtrate was concentrated. Column chromatography (DCM–MeOH, 0→8%) of the residue produced diamine **24** (71 mg, 81%) as a colorless syrup. HRMS (ESI): calcd. for C_31_H_42_F_3_N_3_O_8_ [M + H]^+^ *m/z* 642.2997; found *m/z* 642.2991.

Formic acid (25.5 μL, 0.663 mmol) and DCC (69 mg, 0.334 mmol) were added to a solution of diamine **24** (71 mg, 0.111 mmol) in a mixture of DCM–MeOH (9:1, 4 mL). The mixture was stirred for 1 h, the solvents were evaporated, and the residue was suspended in DCM (4 mL). Dicyclohexylurea was filtered off and washed with DCM (3 × 3 mL), the filtrate was concentrated, and the residue was purified by column chromatography (DCM–MeOH, 0→7%) to give bis(formamide) **25** (68 mg, 88%) as a white amorphous solid. HRMS (ESI): calcd. for C_33_H_42_F_3_N_3_O_10_ [M + NH_4_]^+^ *m/z* 715.3161; found *m/z* 715.3167.

A mixture of compound **25** (68 mg, 0.097 mmol) and Pd(OH)_2_/C (20 mg) in MeOH (3 mL) was stirred under hydrogen for 2 h at room temperature. The catalyst was remove by filtration and washed with MeOH (4 × 4 mL), and the filtrate was concentrated. Column chromatography of the residue afforded triol **26** (46 mg, 92%) as a white amorphous solid. HRMS (ESI): calcd. for C_19_H_30_F_3_N_3_O_10_ [M + Na]^+^ *m/z* 540.1775; found *m/z* 540.1780.

Ambersep 900 (OH^−^) (3 mL) was added to a solution of compound **26** (46 mg, 0.089 mmol) in 50% aqueous MeOH (2 mL). The mixture was kept for 1 h with periodic shaking, and then the resin was filtered off and washed with 50% aqueous MeOH (6 × 3 mL). The filtrate was concentrated and the residue was subjected to gel-permeation chromatography to give title compound **4** (36 mg, 92%) as a white amorphous solid. The data of NMR and mass-spectra were identical to those described previously (Tsvetkov and Nifantiev, 2023).

**3-Aminopropy 4,6-dideoxy-4-formamido-α-d-mannopyranosyl-(1→2)-4,6-dideoxy-4-formamido-α-d-mannopyranosyl-(1→3)-4,6-dideoxy-4-formamido-α-d-mannopyranosyl-(1→2)-4,6-dideoxy-4-formamido-α-d-mannopyranoside (1).** The title compound was synthesized in four steps from protected precursor **19** in total yield of 55% (for details see Supplementary Material). A white fluffy solid; contained a non-stoichiometric amount (0.45 equiv.) of AcOH; [α]_D_ +55 (c 1, water). ^1^H NMR (600 MHz, D_2_O): δ 8.24–8.18 (m, 2.8 H, H_*Z*_CON), 8.06-7.99 (m, 1.2 H, H_*E*_CON), 5.11–4.92 (m, 4 H, 4 H-1), 4.20–3.77 (m, 15.8 H, 4 H-2, 4 H-3, 4 H-4_*Z*_, 4 H-5, C*Ha*HbCH_2_CH_2_N), 3.61–3.56 (m, 1 H, OCHa*Hb*CH_2_CH_2_N), 3.51–3.35 (m, 1.2 H, 4 H-4_*E*_), 3.16–3.07 (m, 2 H, OCH_2_CH_2_C*H*_*2*_N), 2.02–1.95 (m, 2 H, OCH_2_C*H*_*2*_CH_2_N), 1.90 (s, ∼1.4 H, CH_3_COOH), 1.30-1.21 (m, 12 H, 4 H-6). ^13^C NMR (150 MHz, D_2_O): δ 169.0, 168.9 (H_*E*_CON), 166.1, 165.8 (H_*Z*_CON), 103.5, 103.4, 103.1, 102.9, 101.9, 99.6 (4 C-1), 79.4, 79.2, 79.0, 78,7, 78.6, 78,4, 77.5 (C-2^A^, C-2^C^, C-3^B^), 70.1,70.0, 69.9, 69.6, 69.5, 69.2, 69.0, 68.9, 68.7, 68.6, 68.5, 68.3 (C-3^A^, C-5^A^, C-2^B^, C-5^B^, C-3^C^, C-5^C^, C-2^D^, C-3^D^, C-5^D^), 66.4 (O*C*H_2_CH_2_CH_2_N), 58.2, 58.0, 57.9, 56.7 (4 C-4_*E*_), 53.3, 53.1, 52.9, 52.2, 52.1 (4 C-4_*Z*_), 38.6 (OCH_2_CH_2_ *C*H_2_N), 27.9 (OCH_2_ *C*H_2_CH_2_N), 18.4, 18.2, 18.1, 18.0, 17.9 (4 C-6). HRMS (ESI): calcd. for C_31_H_53_N_5_O_17_ [M + H]^+^ *m/z* 768.3509; found *m/z* 768.3510.

**3-Aminopropyl 4,6-dideoxy-4-formamido-α-d-mannopyranosyl-(1→3)-4,6-dideoxy-4-formamido-α-d-mannopyranosyl-(1→2)-4,6-dideoxy-4-formamido-α-d-mannopyranoside (2).** The title compound was obtained in four steps from protected progenitor **17** in total yield of 54% (for details see Supplementary Material). A white fluffy solid; contained a non-stoichiometric amount (0.40 equiv.) of AcOH; [α]_D_ +60 (c 1, water). ^1^H NMR (600 MHz, D_2_O): δ 8.23–8.18 (m, 2.1 H, 3 H_*Z*_CON), 8.06–8.01 (m, 0.9 H, 3 H_*E*_CON), 5.04-4.92 (m, 3 H, 3 H-1), 4.21–3.79 (m, 12.1 H, 3 H-2, 3 H-3, 3 H-4_*Z*_, 3 H-5, OC*Ha*HbCH_2_CH_2_N), 3.61–3.56 (m, 1 H, OCHa*Hb*CH_2_CH_2_N), 3.48 (t, 0.3 H, *J* = 10.1 Hz, H-4_*E*_), 3.43 (t, 0.3 H, *J* = 10.3 Hz, H-4_*E*_), 3.36 (t, 0.3 H, *J* = 10.5 Hz, H-4_*E*_), 3.17–3.07 (m, 2 H, OCH_2_CH_2_C*H*_*2*_N), 2.02–1.95 (m, 2 H, OCH_2_C*H*_*2*_CH_2_N), 1.91 (s, 1.2 H, 0.4 C*H*_*3*_COOH), 1.30–1.21 (m, 9 H, 3 H-6). ^13^C NMR (150 MHz, D_2_O): δ 169.0, 168.9 (3 H_*E*_CON), 165.9, 165.8 (3 H_*Z*_CON), 103.4, 103.2, 103.1, 102.9 (C-1^B^, C-1^C^), 99.4 (C-1^A^), 78.8, 77.8, 77.5, 77.4 (C-2^A^, C-3^B^), 70.3, 70.0, 69.8, 69.7, 69.2, 69.1 69.0, 68.8, 68.7, 68.6, 68.2, (C-2^B^, C-2^C^, C-3^A^, C-3^C^, C-5^A^, C-5^B^, C-5^C^), 66.2 (O*C*H_2_CH_2_CH_2_N), 57.9, 57.7, 56.5 (C-4_*E*_^A^, C-4_*E*_^B^, C-4_*E*_^C^), 53.1, 52.7, 51.6 (C-4_*Z*_^A^, C-4_*Z*_^B^, C-4_*Z*_^C^), 38.4 (OCH_2_CH_2_ *C*H_2_N), 27.7 (OCH_2_ *C*H_2_CH_2_N), 24.3 (*C*H_3_COOH), 17.9, 17.7 (C-6^A^, C-6^B^, C-6^C^). HRMS (ESI): calcd. for C_24_H_42_N_4_O_13_ [M + H]+ *m/z* 595.2821; found *m/z* 595.2819.

**3-Aminopropyl 4,6-dideoxy-4-formamido-α-d-mannopyranosyl-(1→2)-4,6-dideoxy-4-formamido-α-d-mannopyranosyl-(1→3)-4,6-dideoxy-4-formamido-α-d-mannopyranoside (3).** The title trisaccharide was synthesized in four steps from protected precursor **23** in overall yield of 52% (for details see Supplementary Material). A white fluffy solid; contained a non-stoichiometric amount (0.35 equiv.) of AcOH; [α]_D_ +65 (c 1, water). ^1^H NMR (600 MHz, D_2_O): δ 8.22–8.19 (m, 2.2 H, 3 H_*Z*_CON), 8.05-8.01 (m, 0.8 H, 3 H_*E*_CON), 5.09–4.98 (m, 2 H, 2 H-1), 4.83 (d, 1 H, *J*_1,2_ = 2.0 Hz, H-1), 4.15–3.91 (m, 12.2 H, 3 H-2, 3 H-3, 3 H-4_*Z*_, 3 H-5, OC*Ha*HbCH_2_CH_2_N), 3.62–3.57 (m, 1 H, OCHa*Hb*CH_2_CH_2_N), 3.46 (t, 0.25 H, *J* = 10.3 Hz, H-4_*E*_), 3.43-3.35 (m, 0.5 H, 2 H-4_*E*_), 3.17–3.07 (m, 2 H, OCH_2_CH_2_C*H*_*2*_N), 2.03-1.95 (m, 2 H, OCH_2_C*H*_*2*_CH_2_N), 1.91 (s, ∼1 H, 0.35 C*H*_*3*_COOH), 1.29–1.20 (m, 9 H, 3 H-6). ^13^C NMR (150 MHz, D_2_O): δ 169.1, 169.0 (3 H_*E*_CON), 166.2, 166.1 165.9 (3 H_*Z*_CON), 103.6, 103.5, 103.1, 102.1, 102.0, 100.9, 100.8 (C-1^A^, C-1^B^, C-1^C^), 79.4, 79.3, 78.7, 78.3, 77.9 (C-3^A^, C-2^B^), 70.2, 70.0, 69.7, 69.5, 69.3, 69.2, 69.1, 69.0, 68.9, 68.7, 68.6, 68.5, 68.2, 68.1 (C-2^A^, C-5^A^, C-3^B^, C-5^B^, C-2^C^, C-3^C^, C-5^C^), 66.2 (O*C*H_2_CH_2_CH_2_N), 58.1, 57.9, 56.7 (C-4_*E*_^A^, C-4_*E*_^B^, C-4_*E*_^C^), 53.1, 53.0, 52.4 (C-4_*Z*_^A^, C-4_*Z*_^B^, C-4_*Z*_^C^), 38.6 (OCH_2_CH_2_ *C*H_2_N), 27.9 (OCH_2_ *C*H_2_CH_2_N), 24.4 (*C*H_3_COOH), 18.4, 18.3, 18.2, 18.0 (C-6^A^, C-6^B^, C-6^C^). HRMS (ESI): calcd. for C_24_H_42_N_4_O_13_ [M + H]+ *m/z* 595.2821; found *m/z* 595.2815.

**3-Aminopropyl 4,6-dideoxy-4-formamido-α-** **d** **-mannopyranoside (5).** The title glycoside was synthesized in four steps from protected compound **13** in total yield of 57% (for details see Supplementary Material). A colorless glassy solid; contained a non-stoichiometric amount (0.4 equiv.) of AcOH; [α]_D_ +42 (c 1, water). ^1^H NMR (600 MHz, D_2_O): δ 8.19 (s, 0.7 H, H_*Z*_CON), 8.02 (s, 0.4 H, H_*E*_CON), 4.85–4.84 (m, 1 H, H-1), 3.98 (dd, 0.3 H, *J*_2,1_ = 2.0 Hz, *J*_2,3_ = 3.2 Hz, H-2_*E*_), 3.96 (t, 0.7 H, *J* = 2.4 Hz, H-2_*Z*_), 3.91-3.77 (m, 3.7 H, H-3, H-4_*Z*_, H-5, OC*Ha*HbCH_2_CH_2_N), 3.62–3.57 (m, 1 H, OCHa*Hb*CH_2_CH_2_N), 3.36 (t, 0.3 H, *J* = 10.1 Hz, H-4_*E*_), 3.17–3.08 (m, 2 H, OCH_2_CH_2_C*H*_*2*_N), 2.03–1.95 (m, 2 H, OCH_2_C*H*_*2*_CH_2_N), 1.90 (s, 1.2 H, CH_3_COO^–^), 1.26 (d, 0.9 H, *J*_6,5_ = 6.4 Hz, H-6_*E*_), 1.22 (d, 2.1 H, *J*_6,5_ = 6.3 Hz, H-6_*Z*_). ^13^C NMR (150 MHz, D_2_O): δ 169.0 (H_*E*_CON), 166.1 (H_*Z*_CON), 100.9 (C-1), 70.3 (C-2), 69.2 (C-3), 68.5 (C-5_*Z*_), 68.2 (C-5_*E*_), 66.2 (O*C*H_2_CH_2_CH_2_N), 57.9 (C-4_*E*_), 53.2 (C-4_*Z*_), 38.7 (OCH_2_CH_2_ *C*H_2_N), 27.8 (OCH_2_ *C*H_2_CH_2_N), 18.0 (C-6_*Z*_), 17.9 (C-6_*E*_). HRMS (ESI): calcd. for C_10_H_20_N_2_O_5_ [M + H]^+^ *m/z* 249.1445; found *m/z* 249.1449.

**3-Aminopropy α-d-rhamnopyranosyl-(1→2)-4,6-dideoxy-4-formamido-α-d-mannopyranosyl-(1→2)-4,6-dideoxy-4-formamido-α-d-mannopyranosyl-(1→3)-4,6-dideoxy-4-formamido-α-d-mannopyranosyl-(1→2)-4,6-dideoxy-4-formamido-α-d-mannopyranoside (6).** The title pentasaccharide was obtained in four steps from protected precursor **29** in total yield of 45% (for details see Supplementary Material). A white fluffy solid; contained a non-stoichiometric amount (0.35 equiv.) of AcOH; [α]_D_ +55 (c 1, water). ^1^H NMR (600 MHz, D_2_O): δ 8.25–8.19 (m, 2.9 H, 3 H_*Z*_CON), 8.07–8.00 (m, 1.1 H, 3 H_*E*_CON), 5.19–4.93 (m, 5 H, 5 H-1), 4.20-3.73 (m, 20 H, H, 5 H-2, 5 H-3, 4 H-4_*Z*_, H-4^E^, 5 H-5, OC*Ha*HbCH_2_CH_2_N), 3.62–3.57 (m, 1 H, OCHa*Hb*CH_2_CH_2_N), 3.52–3.39 (m, 1.1 H, 4 H-4_*E*_), 3.15–3.06 (m, 2 H, OCH_2_CH_2_C*H*_*2*_N), 2.01–1.95 (m, 2 H, OCH_2_C*H*_*2*_CH_2_N), 1.91 (s, 1.4 H, C*H*_*3*_COOH), 1.31–1.22 (m, 15 H, 5 H-6). ^13^C NMR (150 MHz, D_2_O): δ 169.0, 168.9 (3 H_*E*_CON), 166.1, 165.7 (3 H_*Z*_CON), 103.3, 103.1, 103.0, 102.1, 102.0, 101.9, 101.7, 99.6 (5 C-1), 79.0, 78.8, 78.6, 78.2, 77.9, 77.6, 77.4 (C-2^A^, C-3^B^, C-2^C^, C-2^D^), 73.2, 71.4, 71.3, 70.4, 70.0, 69.9, 69.7, 69.5, 69.4, 68.9, 68.7, 68.4 (C-3^A^, C-5^A^, C-2^B^, C-5^B^, C-3^C^, C-5^C^, C-3^D^, C-5^D^, C-2^E^, C-3^E^, C-4^E^, C-5^E^), 66.4 (O*C*H_2_CH_2_CH_2_N), 58.2, 58.1, 58.0, 56.6 (4 C-4_*E*_), 53.3, 53.2, 53.1, 52.2, 52.1 (4 C-4_*Z*_), 38.6 (OCH_2_CH_2_ *C*H_2_N), 28.1 (OCH_2_ *C*H_2_CH_2_N), 18.5, 18.3, 18.1, 18.0, 17.9, 17.8 (5 C-6). HRMS (ESI): calcd. for C_37_H_63_N_5_O_21_ [M + H]^+^ *m/z* 914.4088; found *m/z* 914.4089.

## Data Availability

The original contributions presented in the study are included in the article/Supplementary Material, further inquiries can be directed to the corresponding author.
