# Supplementary material for: New synthesis of oligosaccharides modelling the M epitope of the Brucella O-polysaccharide
Source: Front Chem. 2024 Jun 21;12:1424157. doi: 10.3389/fchem.2024.1424157 (PMC11224555; doi:10.3389/fchem.2024.1424157)

Supplementary material

**1. Transformation of protected oligosaccharides into free 3-aminopropyl
glycosides S2**

**2. ^1^H and ^13^C NMR spectra of synthesized compounds S10**

**1. Transformation of protected oligosaccharides into free 3-aminopropyl glycosides**

**3-Aminopropy 4,6-dideoxy-4-formamido-α-d-mannopyranosyl-(1→2)-4,6-dideoxy-4-formamido-α-d-mannopyranosyl-(1→3)-4,6-dideoxy-4-formamido-α-d-mannopyranosyl-(1→2)-4,6-dideoxy-4-formamido-α-d-mannopyranoside (1).** Compound **19** (130 mg, 0.107 mmol) in MeOH (5 mL) was hydrogenated in the presence of Pd(OH)_2_/C (55 mg) at 35 °C for 4 h. The catalyst was filtered off and washed with MeOH (5×4 mL), the filtrate was concentrated, and the residue was purified by column chromatography (DCM – MeOH, 5→17%) to yield the tetramine **S1** (82 mg, 69%) as a glassy solid. HRMS (ESI): calcd. for C_57_H_76_F_3_N_5_O_14_ [M + H]^+^ *m/z* 1112.5414; found *m/z* 1112.5393.

Formic acid (33 μL, 0.885 mmol) and a solution of DCC (91 mg, 0.441 mmol) in DCM and MeOH (9:1, 1 mL) were added to a solution of tetramine **S1** (82 mg, 0.074 mmol) in the same solvent mixture (4 mL). The mixture was stirred for 1 h, and then the solvents were evaporated. The residue was taken in DCM (4 mL), the precipitate of dicyclohexylurea was filtered off and washed with DCM (4×2 mL). The filtrate was concentrated, and the residue was subjected to column chromatography (DCM – MeOH, 0→10%) to produced the N-formylated product **S2** (86 mg, 96%) as a white amorphous solid. HRMS (ESI): calcd. for C_61_H_76_F_3_N_5_O_18_ [M + Na]^+^ *m/z* 1246.5030; found *m/z* 1246.5016.

A mixture of compound **S2** (86 mg, 0.070 mmol) and Pd(OH)_2_/C (40 mg) in MeOH (4 mL) was stirred under hydrogen for 2 h. The catalyst was filtered off and washed with MeOH (4×4 mL), the filtrate was concentrated, and the residue was chromatographed (DCM – MeOH, 25→40%) to afford the debenzylation product **S3** (55 mg, 91%) as a white amorphous solid. HRMS (ESI): calcd. for C_33_H_52_F_3_N_5_O_18_ [M + H]^+^ *m/z* 864.3332; found *m/z* 864.3328.

Ambersep 900 (OH^–^) (3 mL) was added to a solution of compound **S3**(55 mg, 0.064 mmol) in 50% aqueous MeOH (3 mL), and the mixture was kept for 1.5 h with periodical shaking. The anionite was filtered off and washed with 50% aqueous MeOH (5×4 mL), and the filtrate was concentrated. The residue was subjected to gel-permeation chromatography, the fraction, containing the target product, were pooled and lyophilized to provide tetrasaccharide **1** (46 mg, 91%) as a white fluffy solid; contained a non-stoichiometric amount (0.45 equiv.) of AcOH.

**3-Aminopropyl 4,6-dideoxy-4-formamido-α-d-mannopyranosyl-(1→3)-4,6-dideoxy-4-formamido-α-d-mannopyranosyl-(1→2)-4,6-dideoxy-4-formamido-α-d-mannopyranoside (2).** Pd(OH)_2_/C (50 mg) was added to a solution of triazide **17** (124 mg, 0.130 mmol) in MeOH (5 mL) and the mixture was stirred at 35 °C for 3.5 h. The catalyst was filtered off and washed with MeOH (5×4 mL), and the filtrate was concentrated. After chromatographic purification (DCM – MeOH, 8→16%) of the residue, triamine **S4** (83 mg, 73%) was obtained as a colorless syrup. HRMS (ESI): calcd. for C_44_H_59_F_3_N_4_O_11_ [M + H]+ *m/z* 877.4205; found *m/z* 877.4211.

Formic acid (32 μL, 0.85 mmol) and a solution of DCC (88 mg, 0.426 mmol) in DCM and MeOH (9:1, 1 mL) were added to a solution of triamine **S4** (83 mg, 0.095 mmol) in the same solvent mixture (4 mL). The mixture was stirred for 1 h, and the solvents were evaporated. The residue was taken in DCM (4 mL), dicyclohexylurea was filtered off and washed with DCM (3×3 mL). The filtrate was concentrated, and the residue was purified by column chromatography (DCM – MeOH, 2→8%) to give formamide **S5** (78 mg, 86%) as a colorless amorphous solid. HRMS (ESI): calcd. for C_47_H_59_F_3_N_4_O_14_ [M + NH_4_]+ *m/z* 978.4318; found *m/z* 978.4318.

A mixture of compound **S5** (78 mg, 0.081 mmol) and Pd(OH)_2_/C (40 mg) in MeOH (4 mL) was stirred in a hydrogen atmosphere at room temperature for 2 h. The catalyst was filtered off and washed with MeOH (5×3 mL), the filtrate was concentrated, and the residue was chromatographed (DCM – MeOH, 20 → 25%) to produce debenzylation product **S6** (50 mg, 89%) as a white amorphous solid. HRMS (ESI): calcd. for C_26_H_41_F_3_N_4_O_14_ [M + Na]+ *m/z* 713.2464; found *m/z* 713.2462.

Ambersep 900 (OH^–^) (3 mL) was added to a solution of compound **S6** (50 mg, 0.072 mmol) in 50% aqueous MeOH (4 mL), and the mixture was kept for 1 h with periodical shaking. The resin was filtered off and washed with 50% aqueous MeOH (6×3 mL), the filtrate was concentrated. The residue was subjected to gel-permeation chromatography, the fraction, containing the target product, were pooled and lyophilized to provide trisaccharide **2** (43 mg, 96%) as a white fluffy solid; contained a non-stoichiometric amount (0.40 equiv.) of AcOH.

**3-Aminopropyl 4,6-dideoxy-4-formamido-α-d-mannopyranosyl-(1→2)-4,6-dideoxy-4-formamido-α-d-mannopyranosyl-(1→3)-4,6-dideoxy-4-formamido-α-d-mannopyranoside (3).** A mixture of triazide **23** (134 mg, 0.140 mmol) and Pd(OH)_2_/C (55 mg) in MeOH (5 mL) was stirred in a hydrogen atmosphere at 35 °C for 3 h. The catalyst was filtered off and washed with MeOH (6×4 mL), the filtrate was concentrated, and the residue was chromatographed (DCM – MeOH, 5→13%) to give the triamine **S7** (94 mg, 76%) as a colorless syrup. HRMS (ESI): calcd. for C_44_H_59_F_3_N_4_O_11_ [M + H]+ *m/z* 877.4205; found *m/z* 877.4194.

Formic acid (36 μL, 0.96 mmol) and a solution of DCC (99 mg, 0.48 mmol) in DCM and MeOH (9:1, 1 mL) were added to a solution of triamine **S7** (94 mg, 0.107 mmol) in the same solvent mixture (3 mL). The mixture was stirred for 1 h, the solvents were evaporated, and the residue was suspended in DCM (4 mL). The precipitate of dicyclohexylurea was filtered off and washed with DCM (4×2 mL), the filtrate was concentrated, and the residue was purified by column chromatography (DCM – MeOH, 0→7%) to yield N-formylated **S8** (80 mg, 78%) as a white amorphous solid. HRMS (ESI): calcd. for C_47_H_59_F_3_N_4_O_14_ [M + K]+ *m/z* 999.3611; found *m/z* 999.3619.

Compound **S8** (80 mg, 0.083 mmol) was subjected to hydrogenolysis in the presence of Pd(OH)_2_/C (40 mg) in MeOH (4 mL). When the reaction was completed (1.5 h), the catalyst was filtered off and washed with MeOH (5×4 mL). The filtrate was evaporated, and the residue was purified by column chromatography (DCM – MeOH, 25→30%) to afford debenzylation product **S9** (55 mg, 96%) as a white amorphous solid. HRMS (ESI): calcd. for C_26_H_41_F_3_N_4_O_14_ [M + Na]+ *m/z* 713.2464; found *m/z* 713.2453.

A solution of compound **S9** (55 mg, 0.080 mmol) in 50% aqueous MeOH (3 mL) was treated with Ambersep 900 (OH^–^) (3 mL). After the reaction was completed (1.5 h), the anionite was removed by filtration and washed with 50 aqueous MeOH (6×3 mL). The filtrate was concentrated, and the residue was subjected to gel-permeation chromatography. The fraction, containing the target product, were pooled and lyophilized to produce trisaccharide **3** (45 mg, 92%) as a white fluffy solid; contained a non-stoichiometric amount (0.35 equiv.) of AcOH.

**3-Aminopropyl 4,6-dideoxy-4-formamido-α-d-mannopyranoside (5).** Pd(OH)_2_/C (15 mg) was added to a solution of azide **13** (51 mg, 0.118 mmol) in MeOH (2 mL), and the mixture was stirred under hydrogen at 35 °C for 1.5 h. The catalyst was removed by filtration and washed with MeOH (5×2 mL), and the filtrate was concentrated. Column chromatography (DCM – MeOH, 2→6%) of the residue produced amine **S10** (30 mg, 63%) as a colorless syrup. HRMS (ESI): calcd. for C_18_H_25_F_3_N_2_O_5_ [M + H]+ *m/z* 407.1788; found *m/z* 407.1781.

Formic acid (8.4 μL, 0.222 mmol) and a solution of DCC (22.5 mg, 0.109 mmol) in DCM and MeOH (9:1, 1 mL) were added to a solution of amine **S10** (30 mg, 0.074 mmol) in the same solvent mixture (2 mL). The mixture was stirred for 1 h and concentrated. The residue was taken in DCM (2 mL), the insoluble dicyclohexylurea was filtered off and washed with DCM (3×2 mL), the filtrate was concentrated. Column chromatography (DCM – MeOH, 0→6%) of the residue provided N-formyl derivative **S11** (31 mg, 97%) as a colorless amorphous solid. HRMS (ESI): calcd. for C_19_H_25_F_3_N_2_O_6_ [M + H]+ *m/z* 457.1557; found *m/z* 457.1558.

Compound **S11** (31 mg, 0.071 mmol) in MeOH (3 mL) was subjected to hydrogenolysis in the presence of Pd(OH)_2_/C (10 mg) for 1 h at room temperature. The catalyst was removed by filtration, washed with MeOH (5×3 mL), and the filtrate was concentrated. The residue was purified by column chromatography (DCM – MeOH, 9:1) to give compound **S12** (24 mg, 96%) as a colorless amorphous solid. HRMS (ESI): calcd. for C_12_H_19_F_3_N_2_O_6_ [M + Na]+ *m/z* 367.1087; found *m/z* 367.1082.

Ambersep 900 (OH^–^) (2 mL) was added to a solution of compound **S12** (24 mg, 0.070 mmol) in 50% aqueous MeOH (2 mL) and the mixture was kept for 1 h with periodical shaking. The resin was filtered off and washed with 50% aqueous MeOH (6×3 mL), the filtrate was concentrated, and the residue was lyophilized from 0.1 M aqueous AcOH to produce compound **5** (17 mg, 98%) as a colorless glassy solid.

**3-Aminopropy α-d-rhamnopyranosyl-(1→2)-4,6-dideoxy-4-formamido-α-d-mannopyranosyl-(1→2)-4,6-dideoxy-4-formamido-α-d-mannopyranosyl-(1→3)-4,6-dideoxy-4-formamido-α-d-mannopyranosyl-(1→2)-4,6-dideoxy-4-formamido-α-d-mannopyranoside (6).** A mixture of azide **29** (98 mg, 0.064 mmol) and Pd(OH)_2_/C (35 mg) in MeOH (3 mL) was stirred in a hydrogen atmosphere at 35 °C for 4.5 h. The catalyst was filtered off and washed with MeOH (6×3 mL), and the filtrate was concentrated. The residue was chromatographed (DCM – MeOH, 3→11%) to yield tetramine **S13** (58 mg, 64%) as a colorless syrup. HRMS (ESI): calcd. for C_77_H_98_F_3_N_5_O_18_ [M + H]+ *m/z* 1438.6932; found *m/z* 1438.6926.

Formic acid (18 μL, 0.48 mmol) and a solution of DCC (50 mg, 0.24 mmol) in a mixture of DCM and MeOH (9:1, 1 mL) were added to a solution of amine **S13** (58 mg, 0.040 mmol) in the same solvent mixture (3 mL). After the reaction was completed (1 h), the solvents were evaporated, and the residue was dissolved DCM (3 mL). Dicyclohexylurea was filtered off and washed with DCM (3×2 mL), the filtrate was concentrated, and the residue was chromatographed (DCM – MeOH, 4→7%) to produce N-formyl product **S14** (44 mg, 71%) as a colorless amorphous solid. HRMS (ESI): calcd. for C_81_H_98_F_3_N_5_O_22_ [M + NH_4_]+ *m/z* 1567.6994; found *m/z* 1567.6984.

A mixture of compound **S14** (44 mg, 0.028 mmol) and Pd(OH)_2_/C (25 mg) in MeOH (2.5 mL) was stirred under hydrogen at room temperature for 2.5 h. The catalyst was filtered off and washed with MeOH (5×3 mL). The filtrate was concentrated to give compound **S15** (29 mg, 100%) as a colorless amorphous solid. HRMS (ESI): calcd. for C_39_H_62_F_3_N_5_O_22_ [M + H + K]^2+^ *m/z* 524.6771; found *m/z* 524.6739. Compound **S15** was used in the next step without further purification.

Ambersep 900 (OH^–^) (3 mL) was added to a solution of compound **S15** (29 mg, 0.029 mmol) in 50% aqueous MeOH (3 mL) and the mixture was kept for 1 h with periodical shaking. The resin was filtered off and washed with 50% aqueous MeOH (6×3 mL), and the filtrate was concentrated. The residue was subjected to gel-permeation chromatography, the fraction, containing the target product, were pooled and lyophilized to give pentasaccharide **6** (26 mg, 100%) as a white fluffy solid; contained a non-stoichiometric amount (0.35 equiv.) of AcOH.


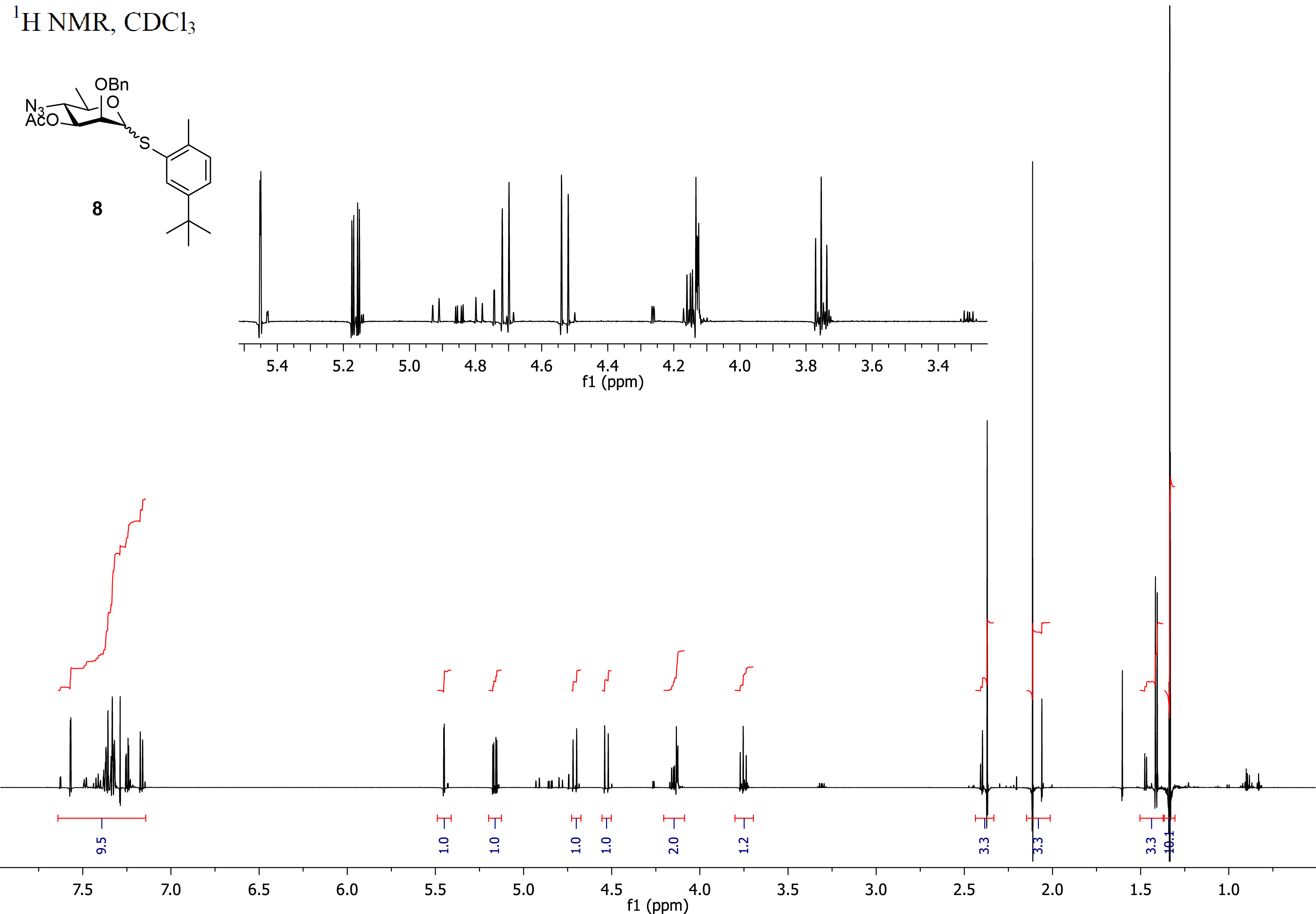


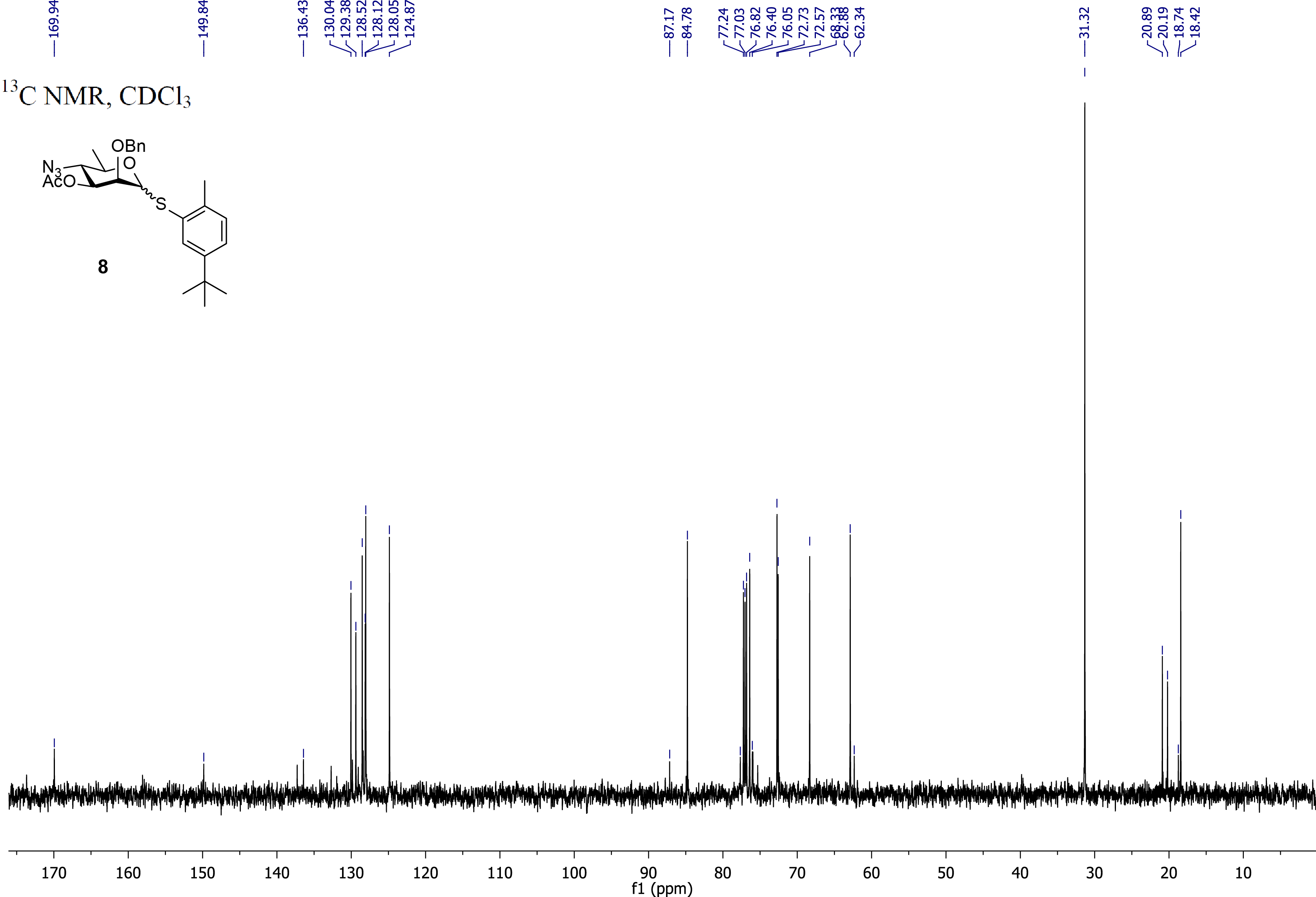


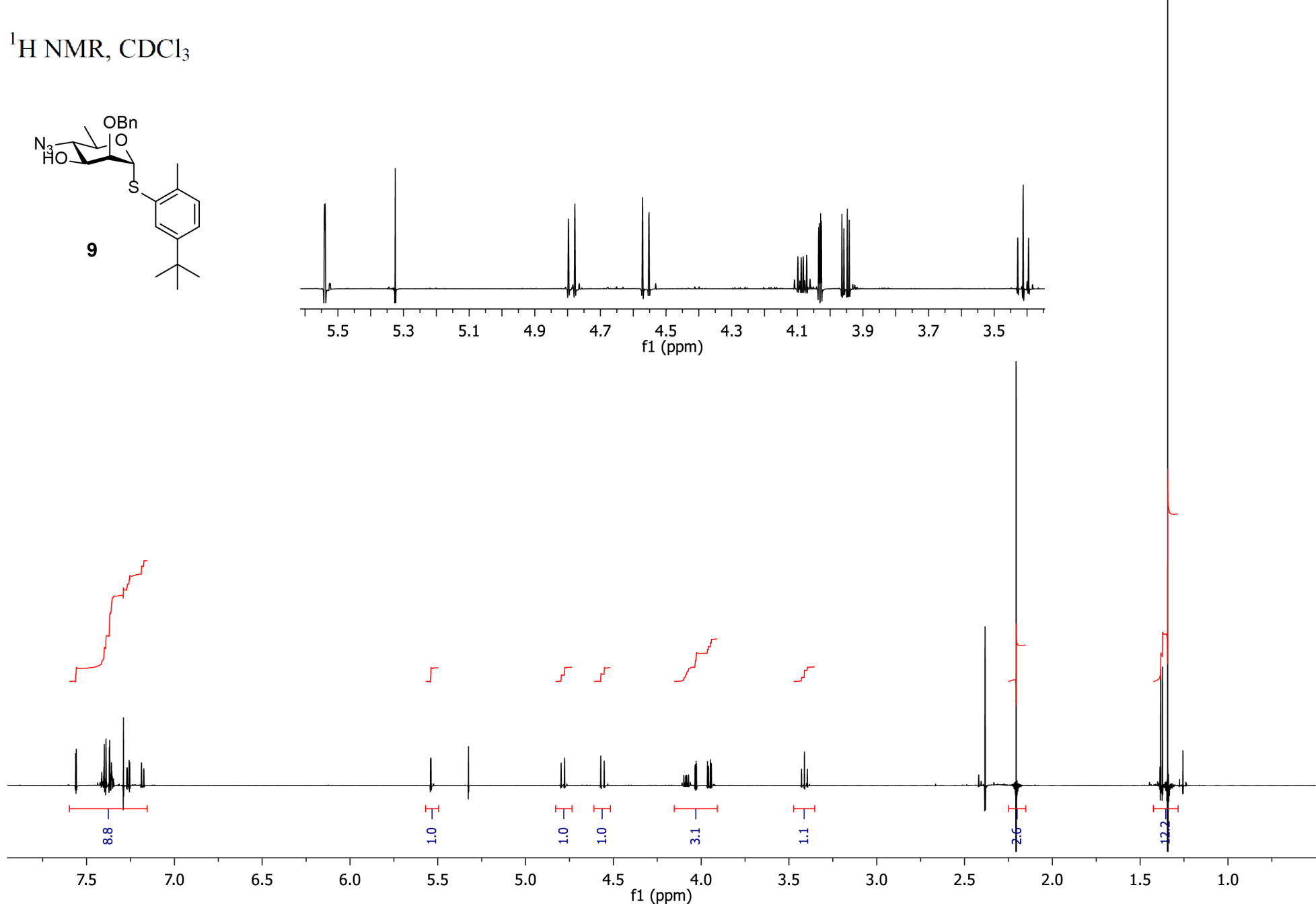


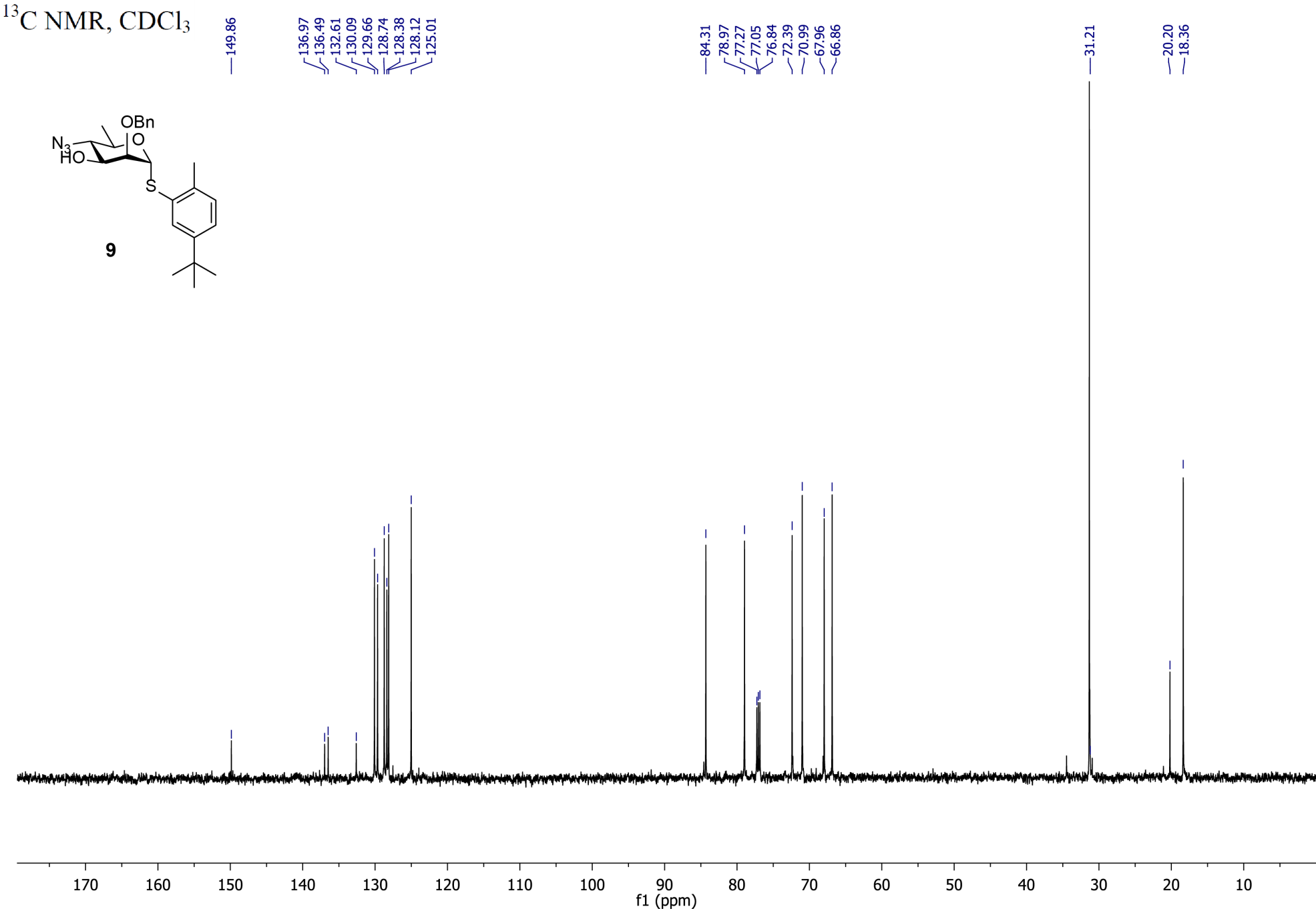


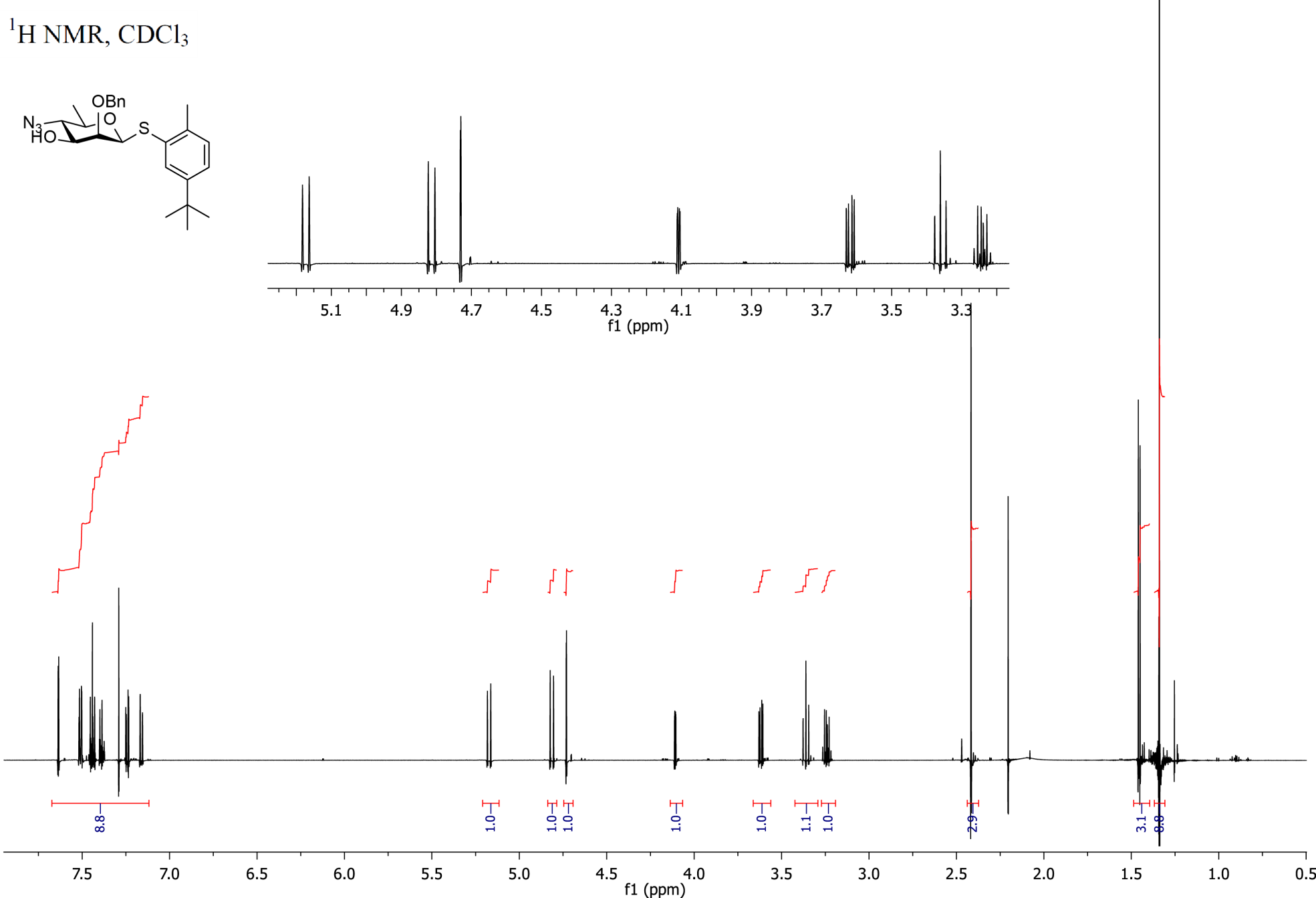


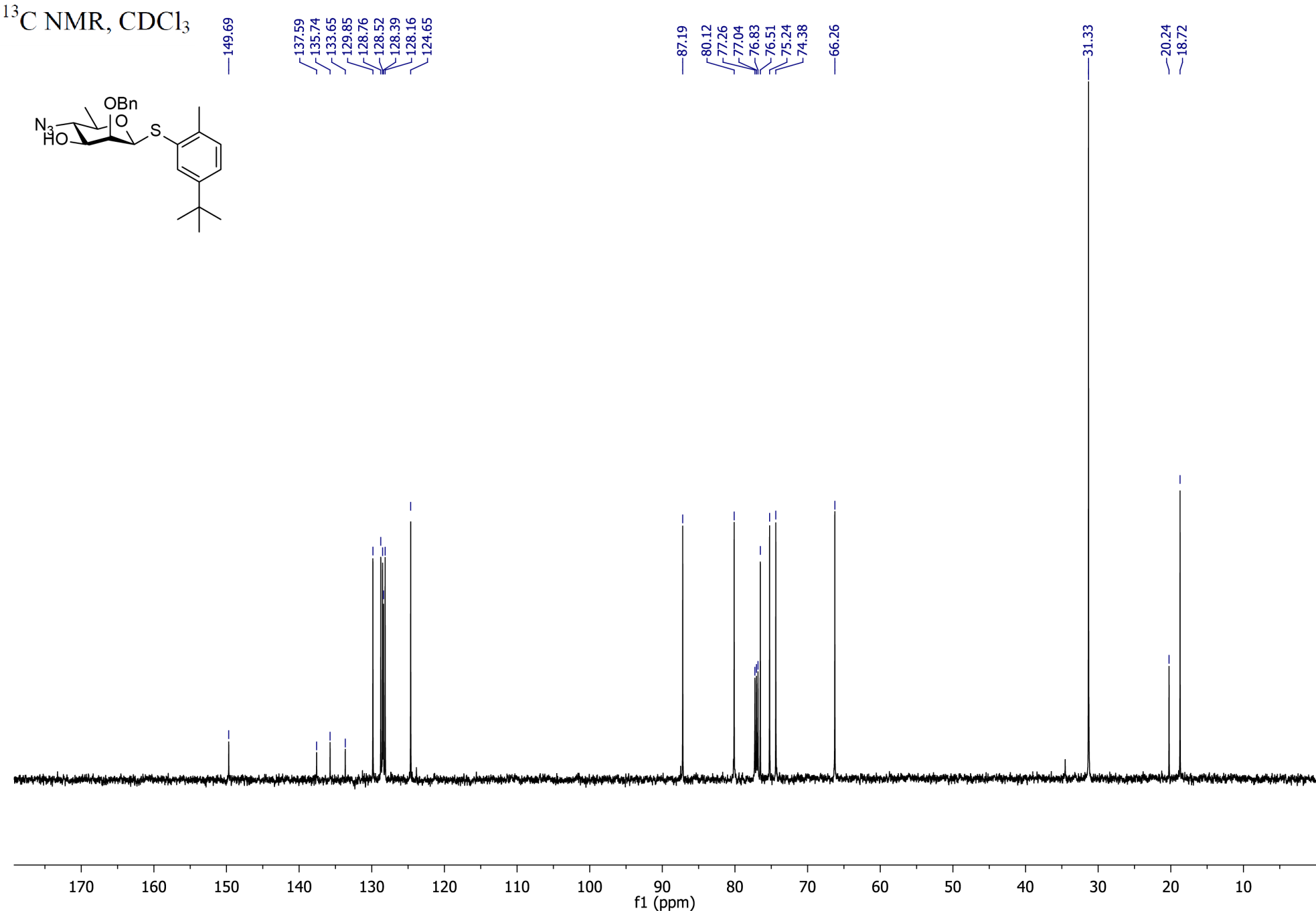


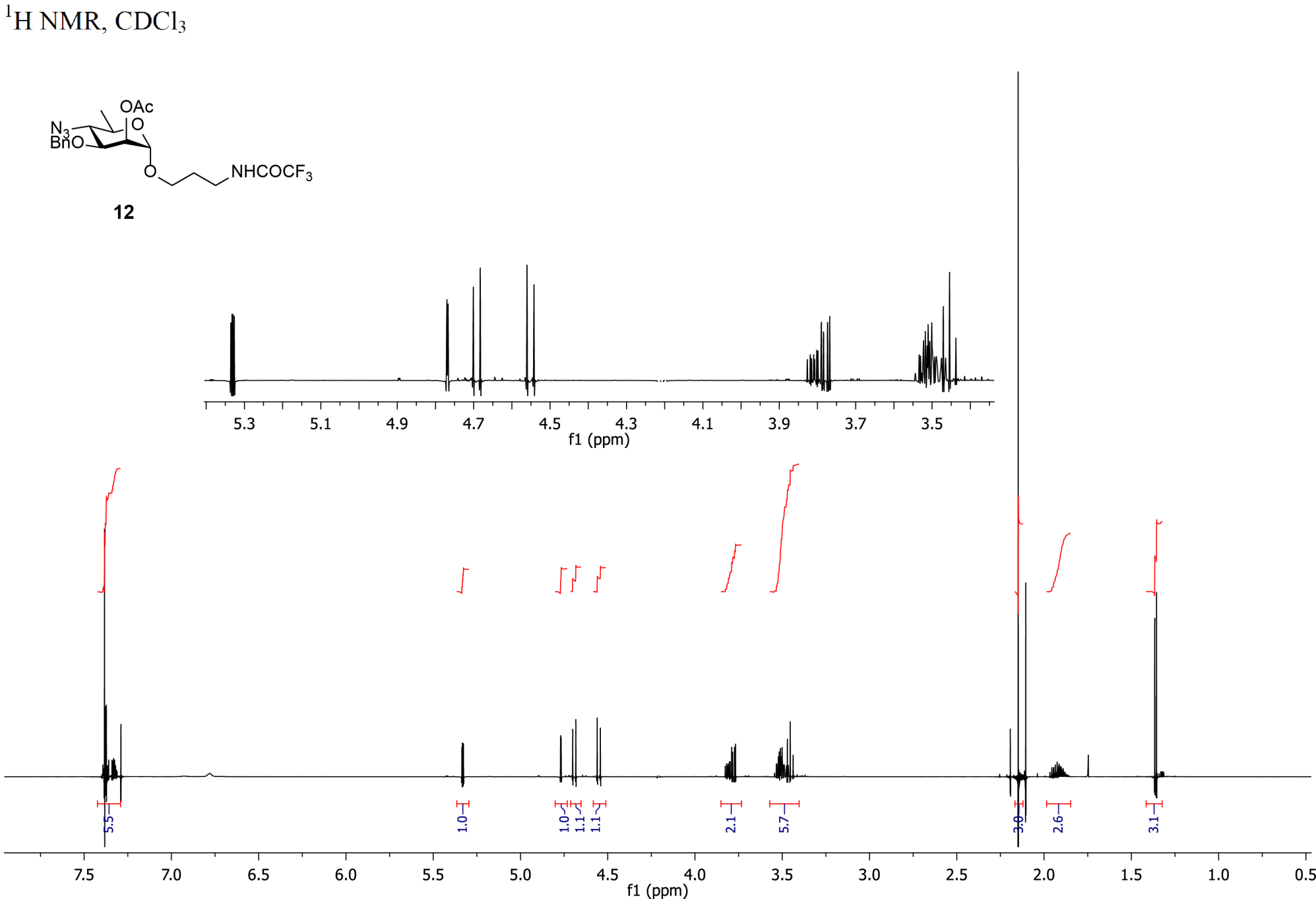


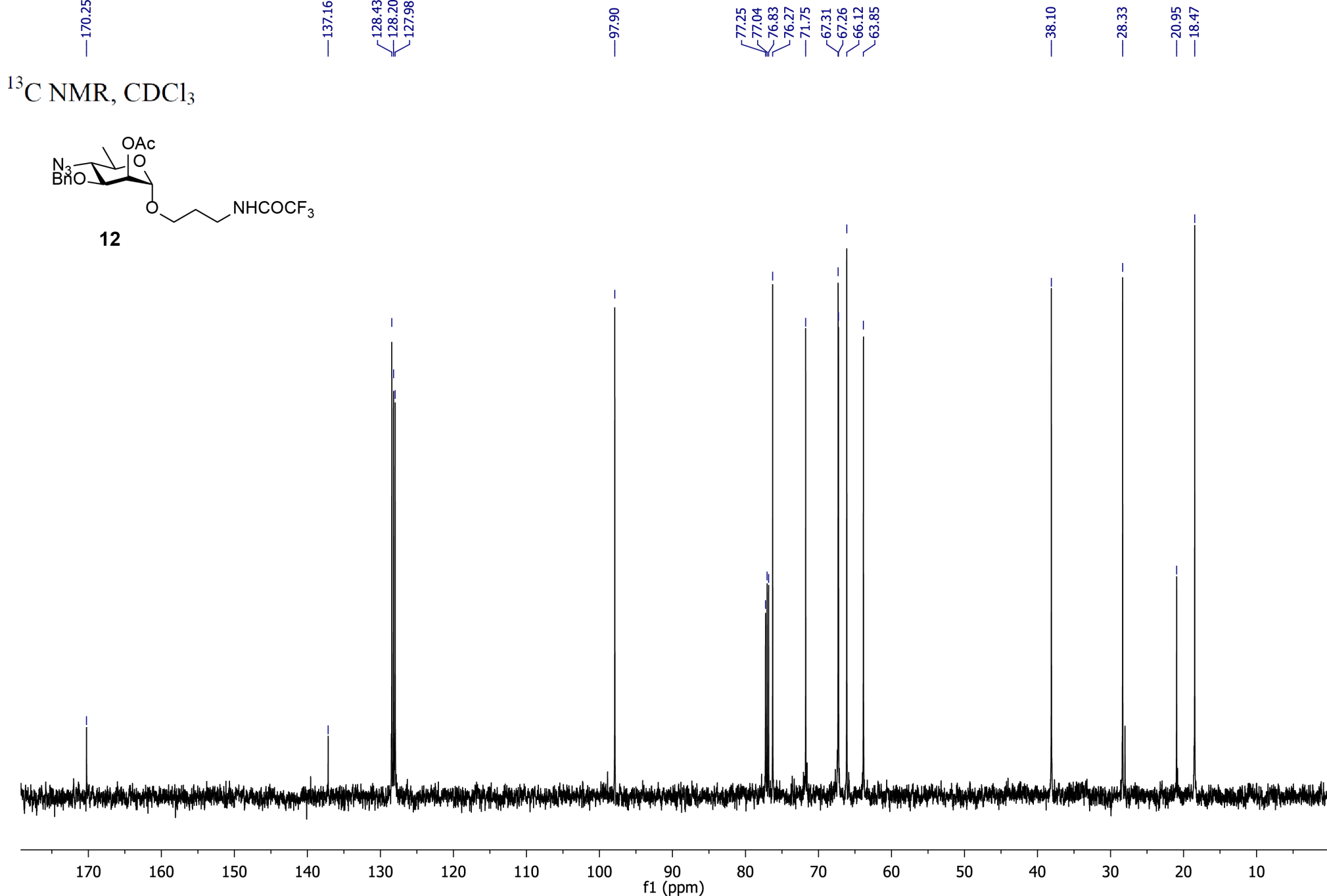


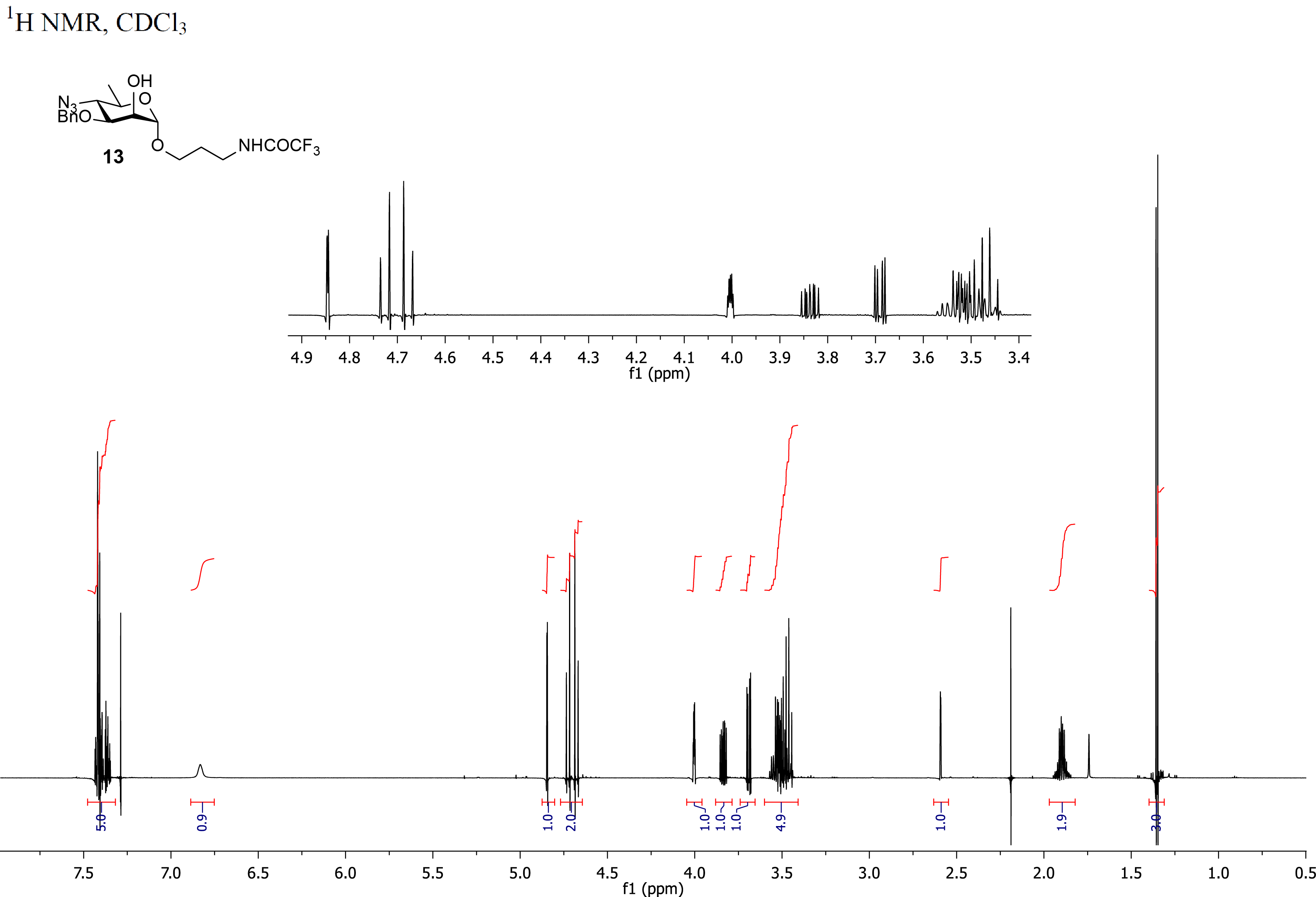


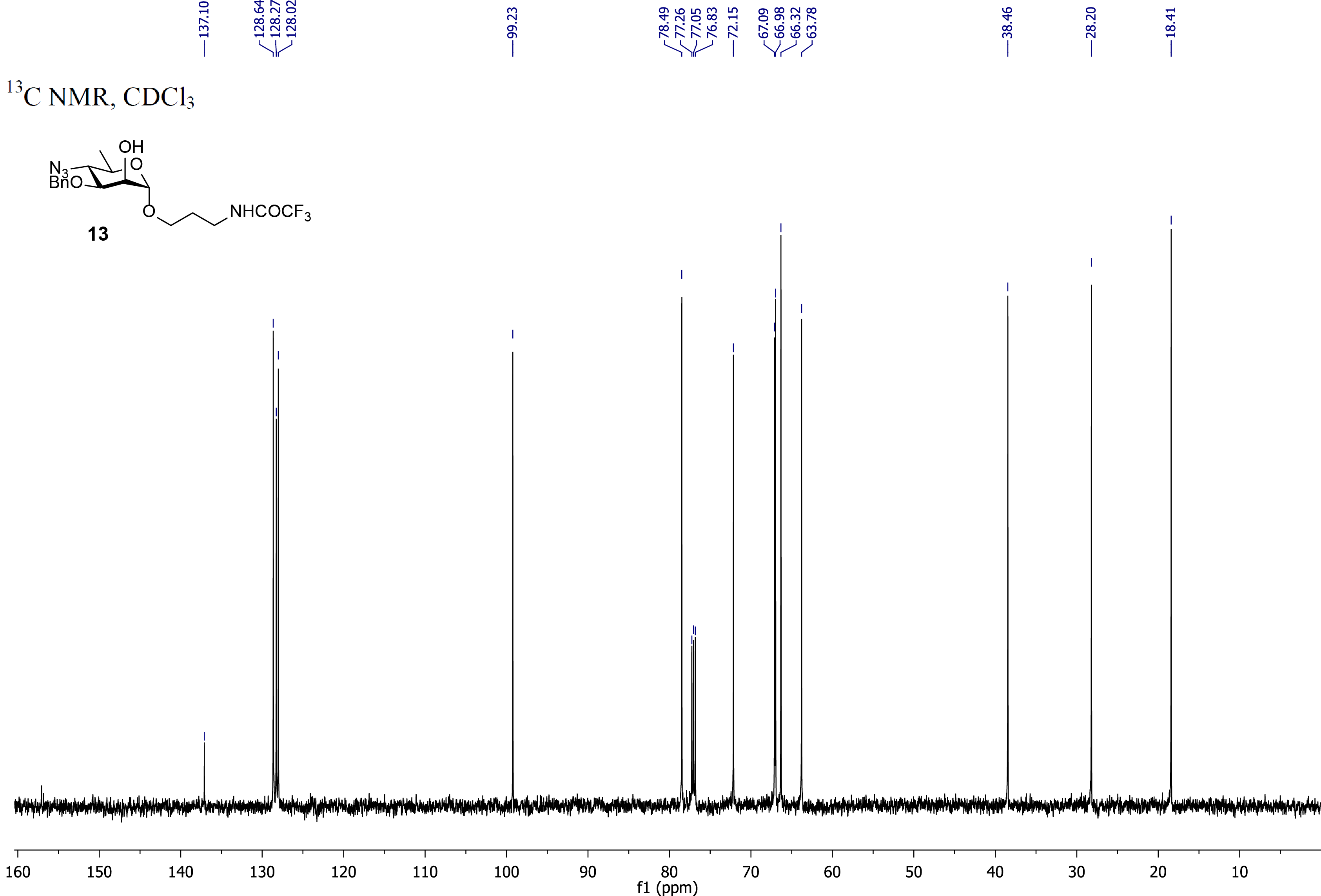


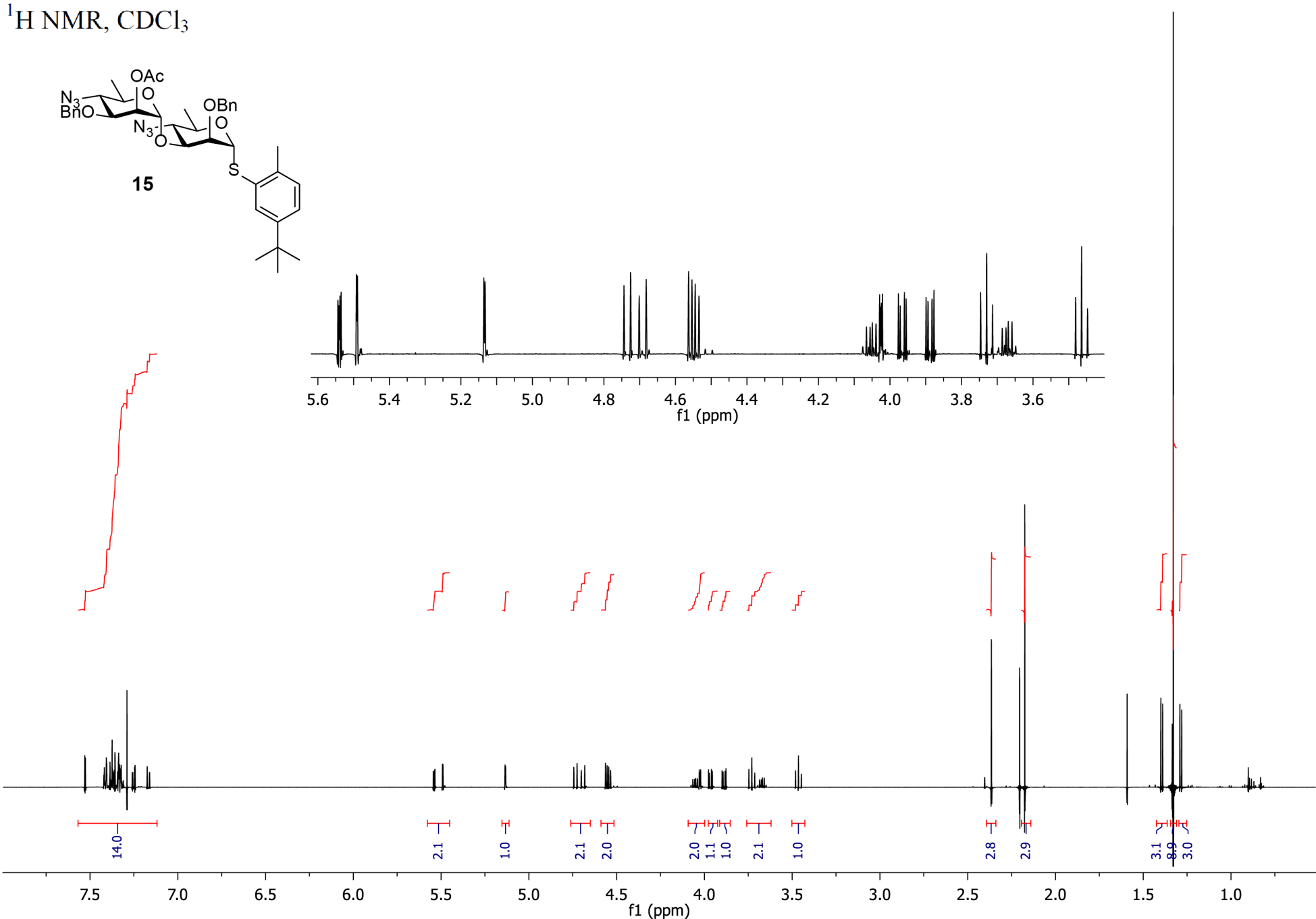


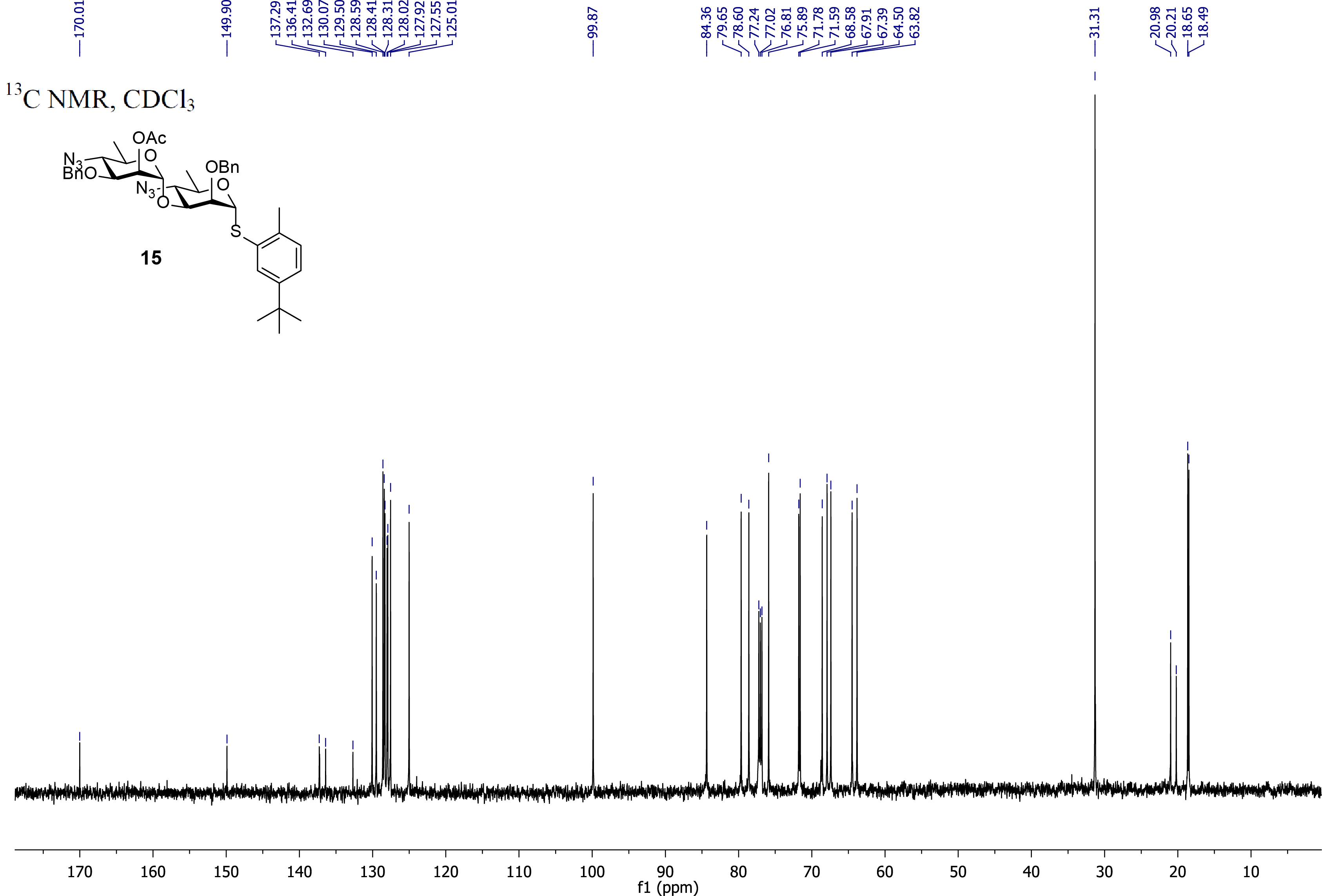


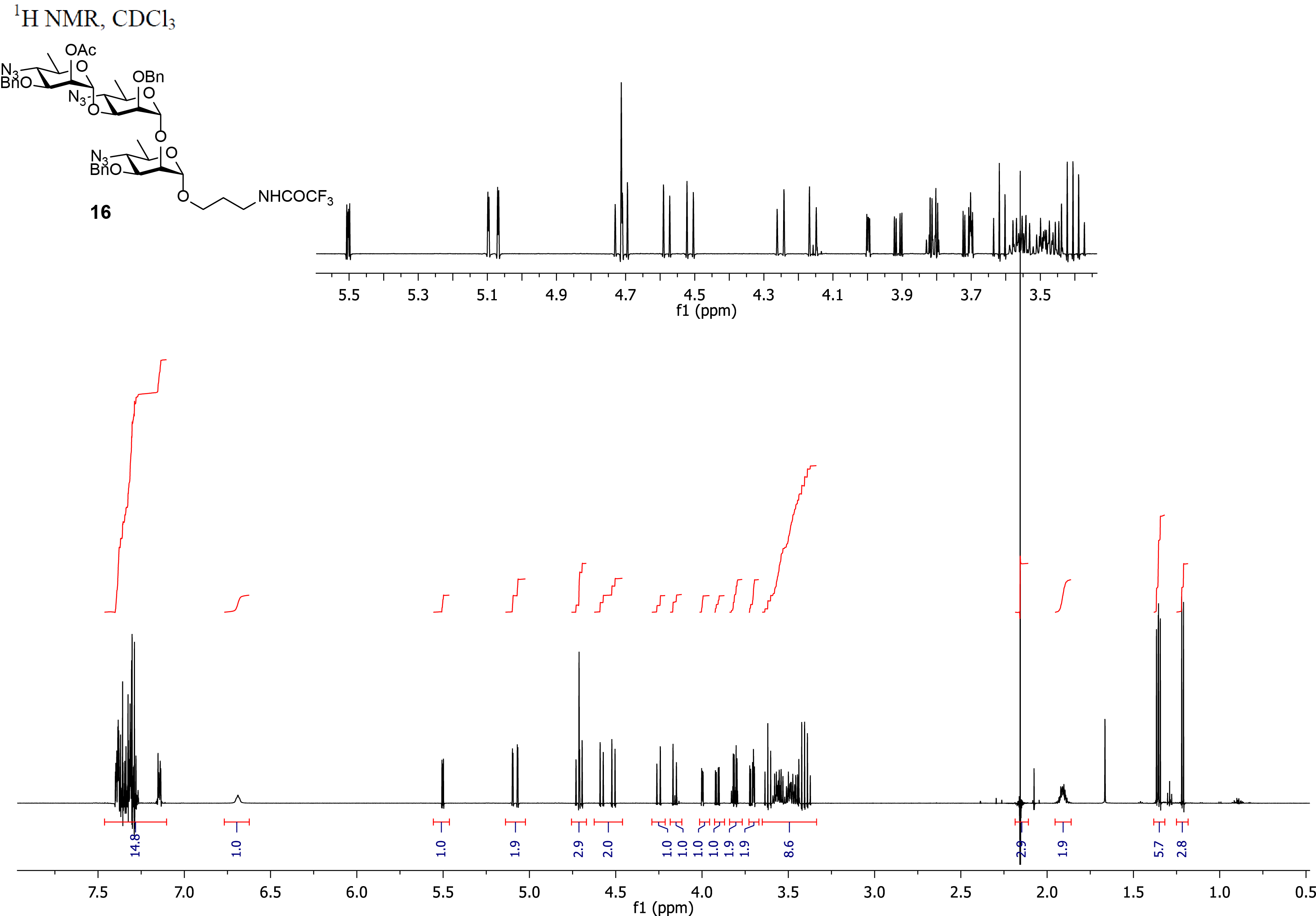


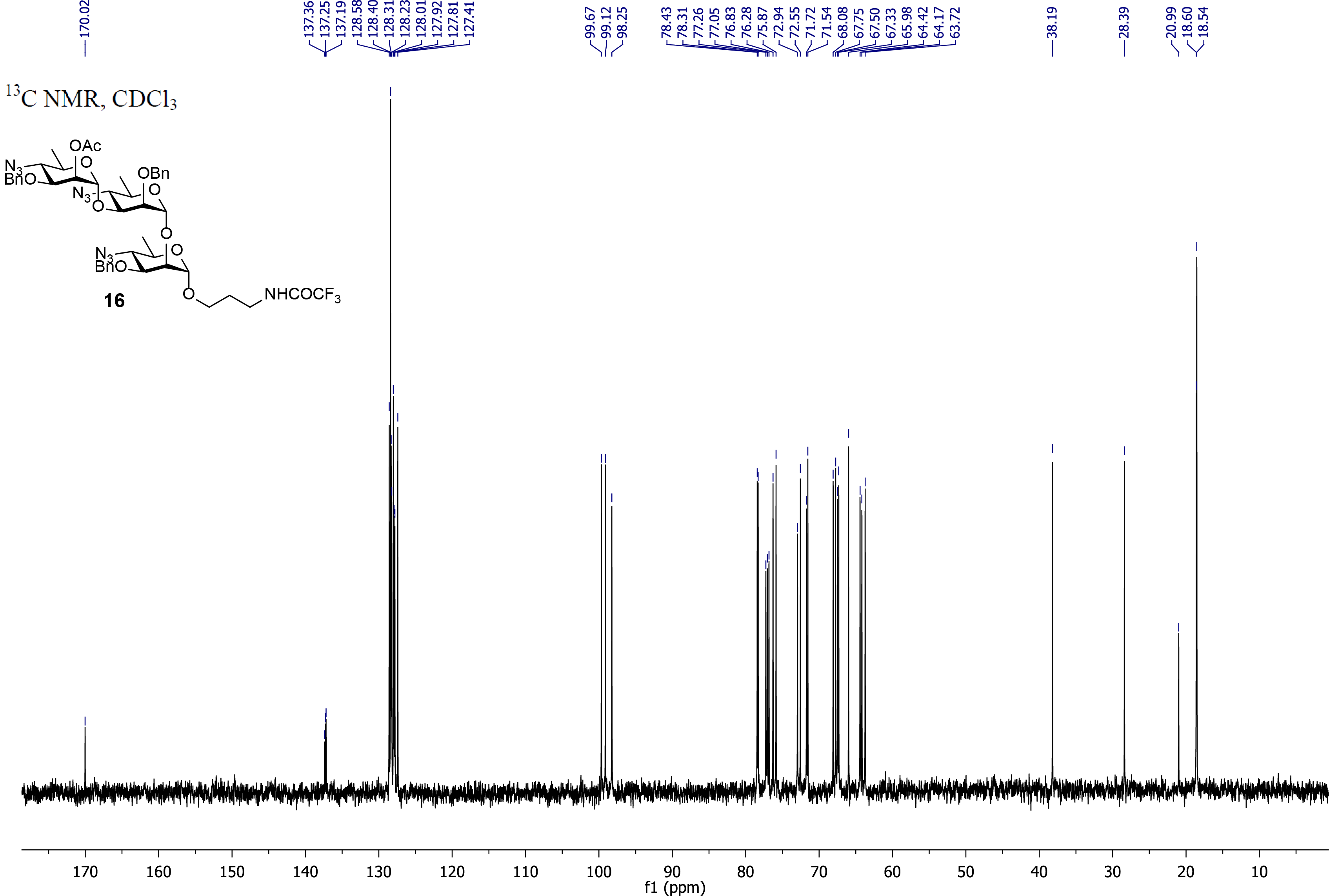


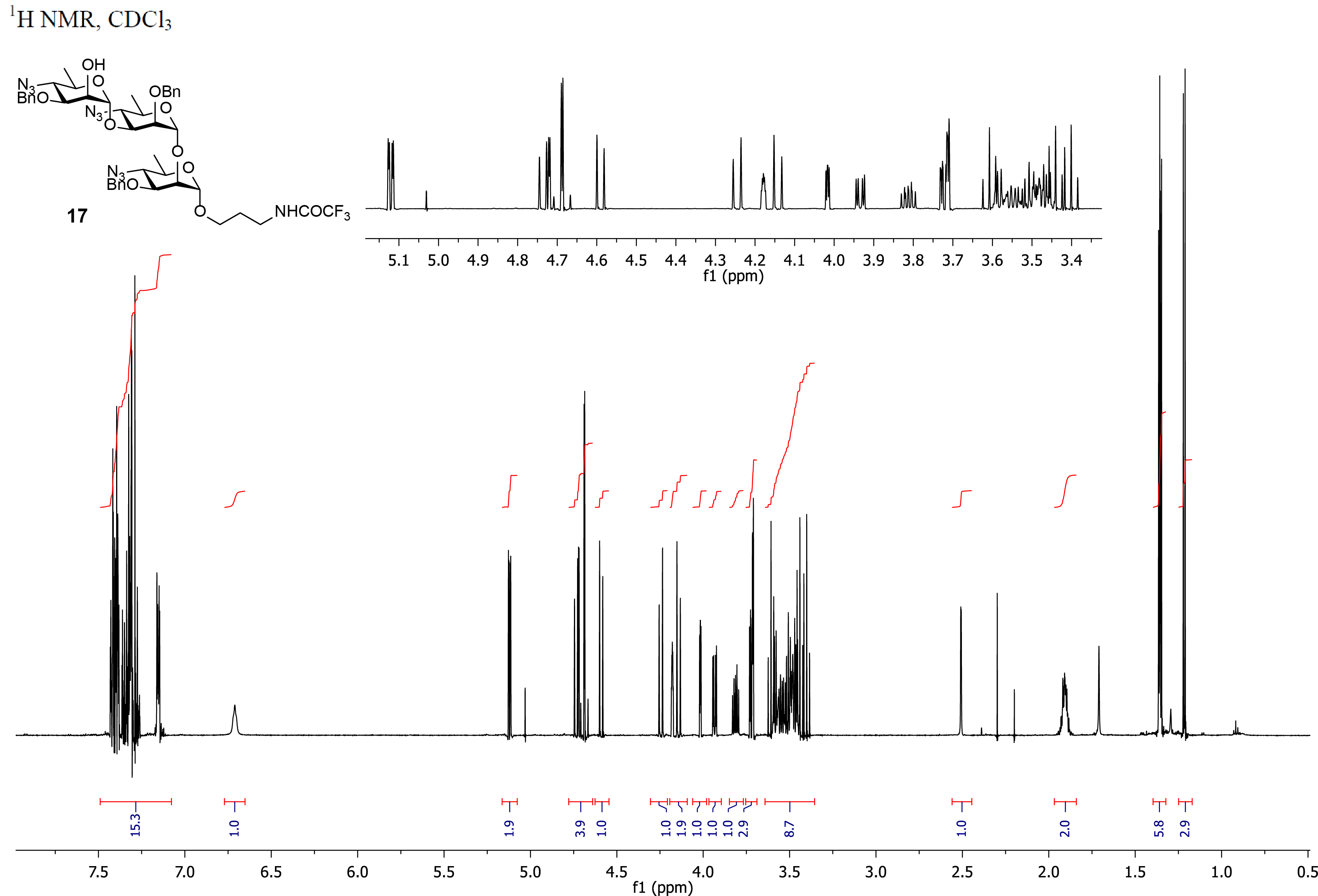


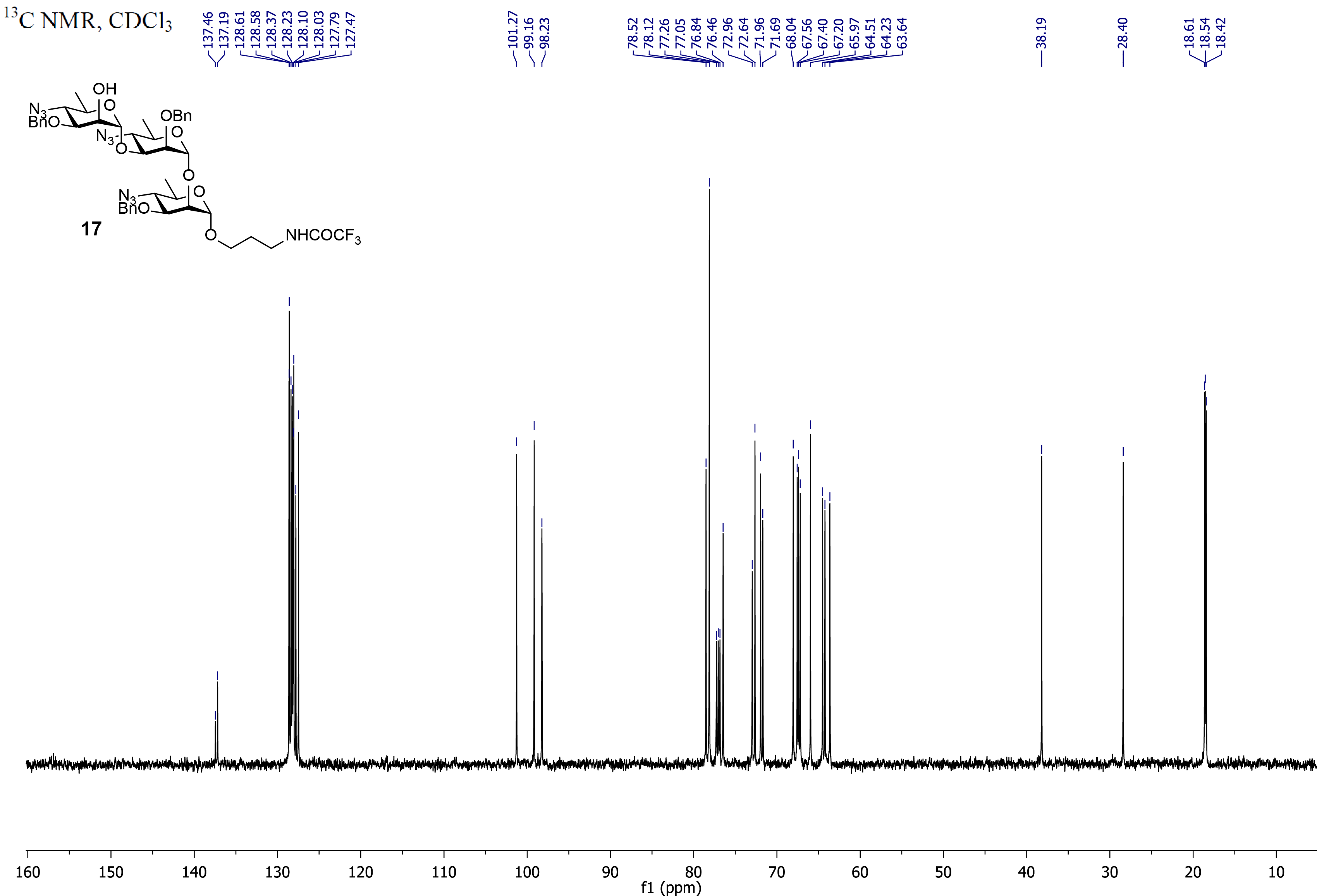


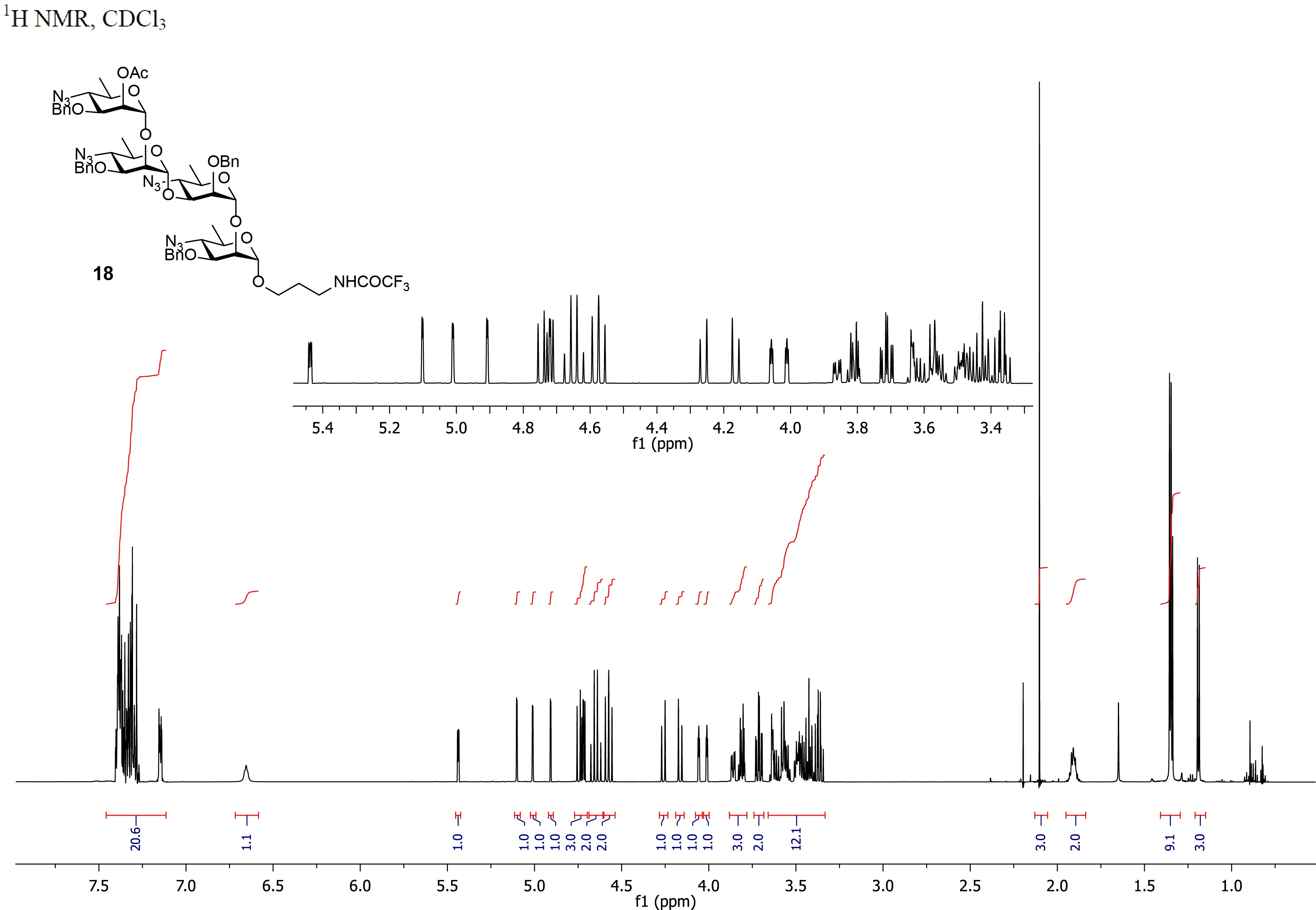


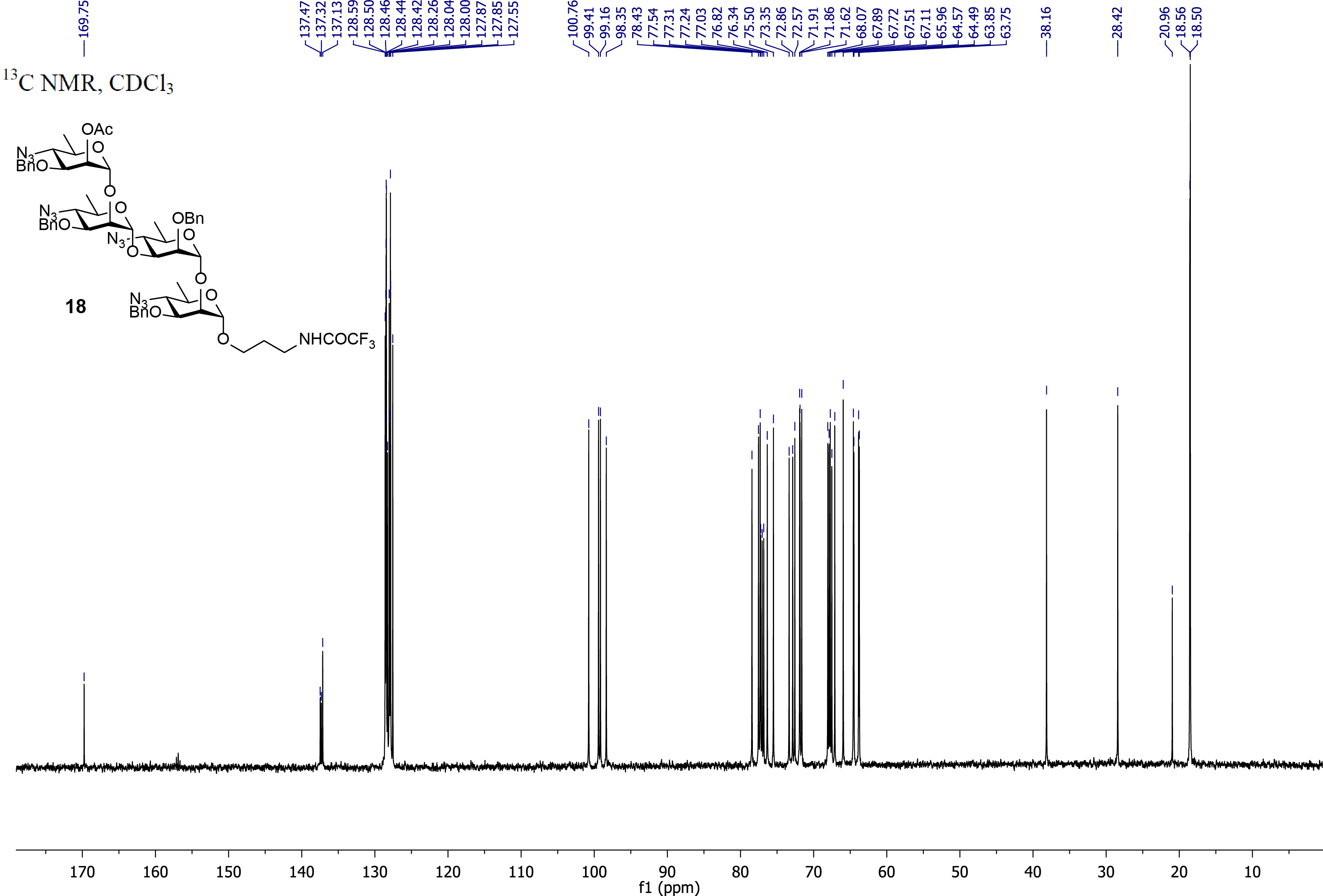


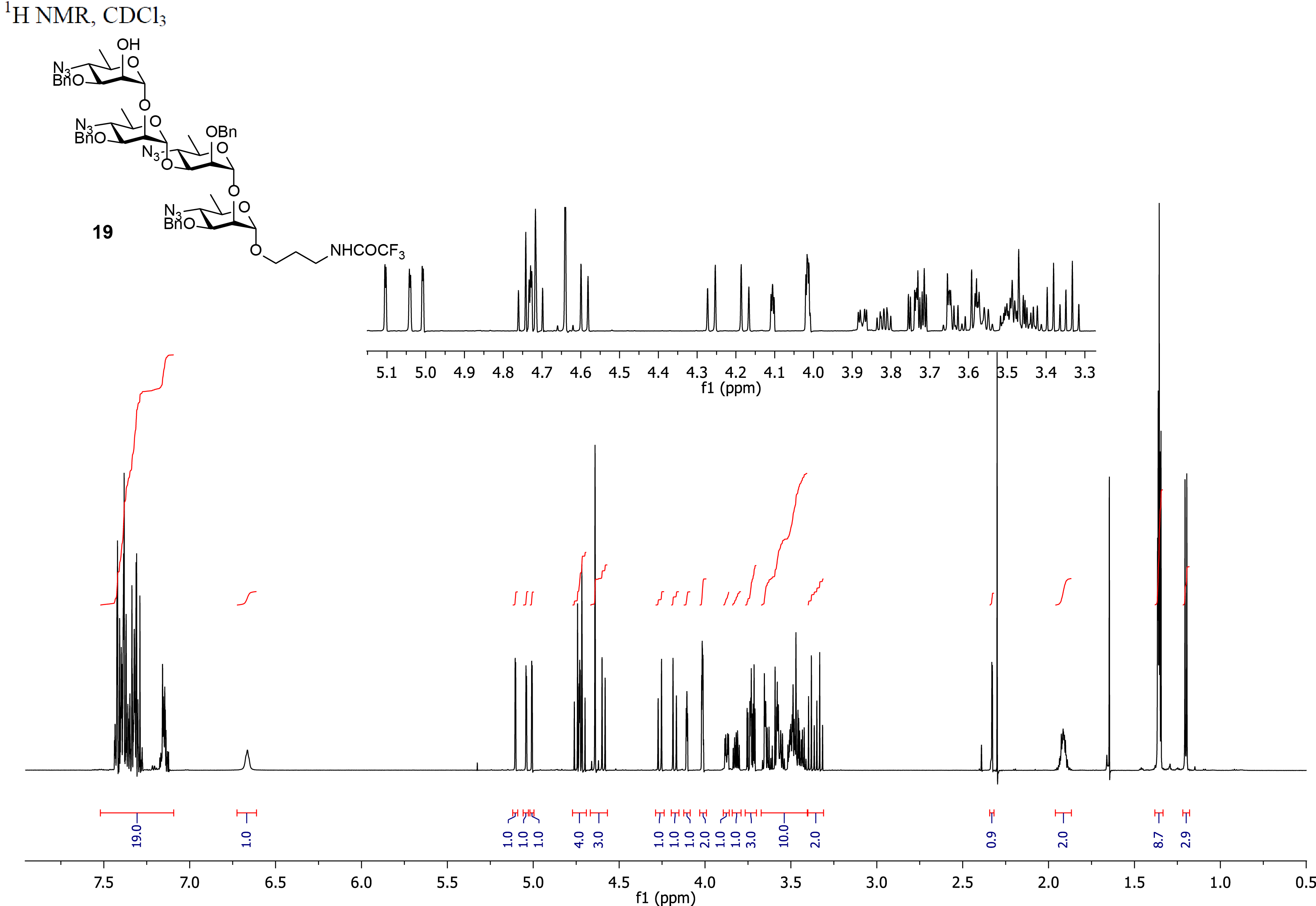


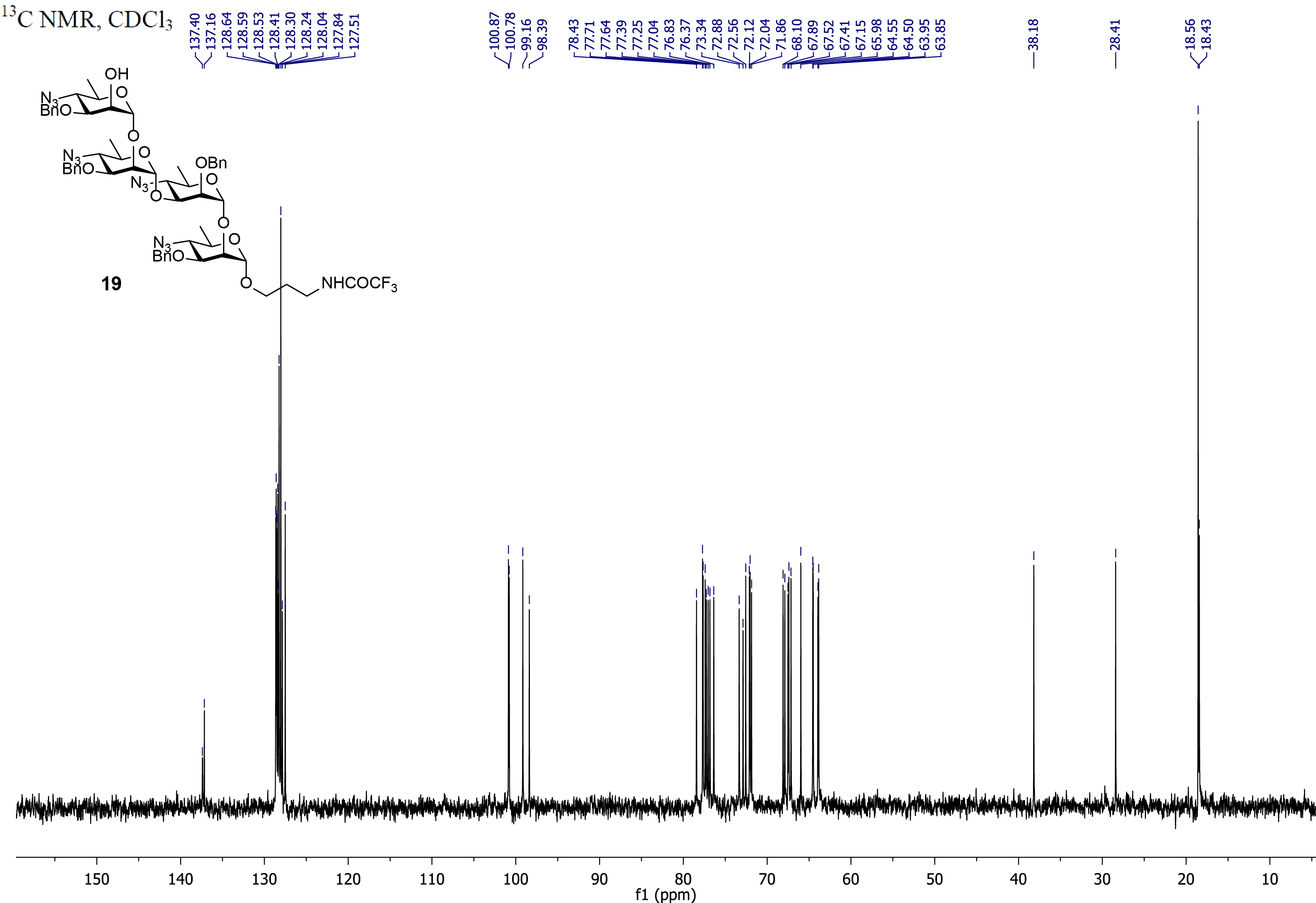


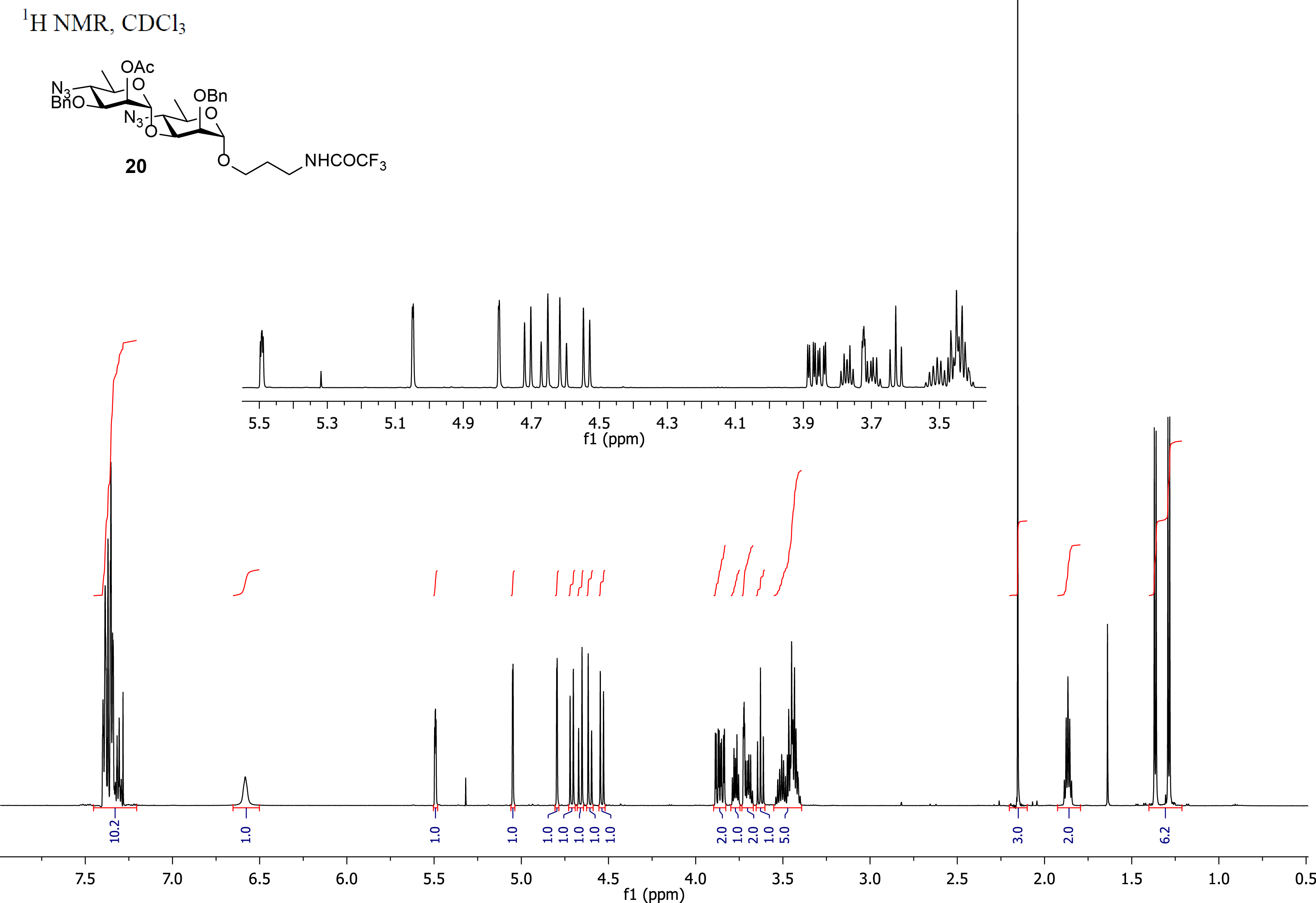


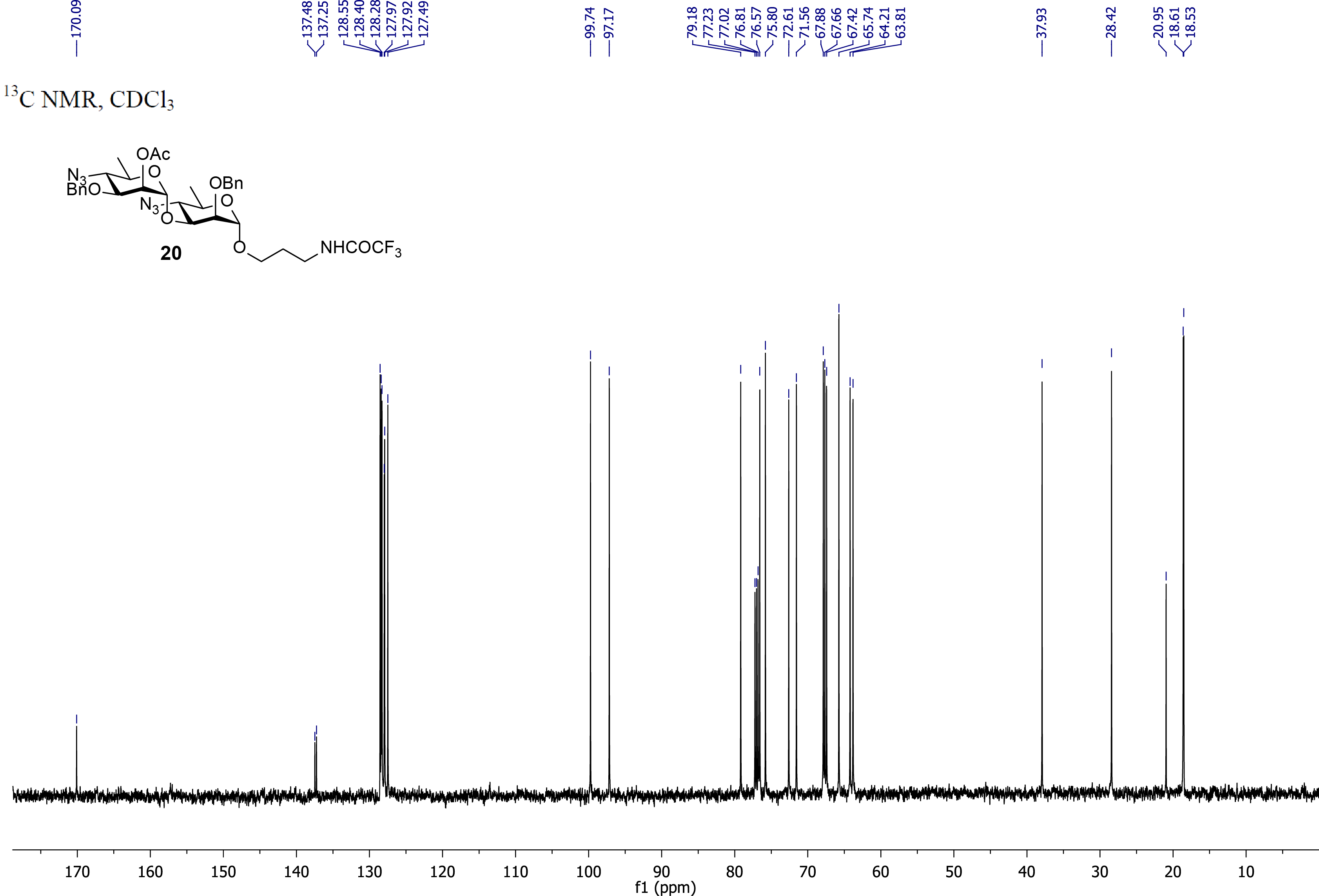


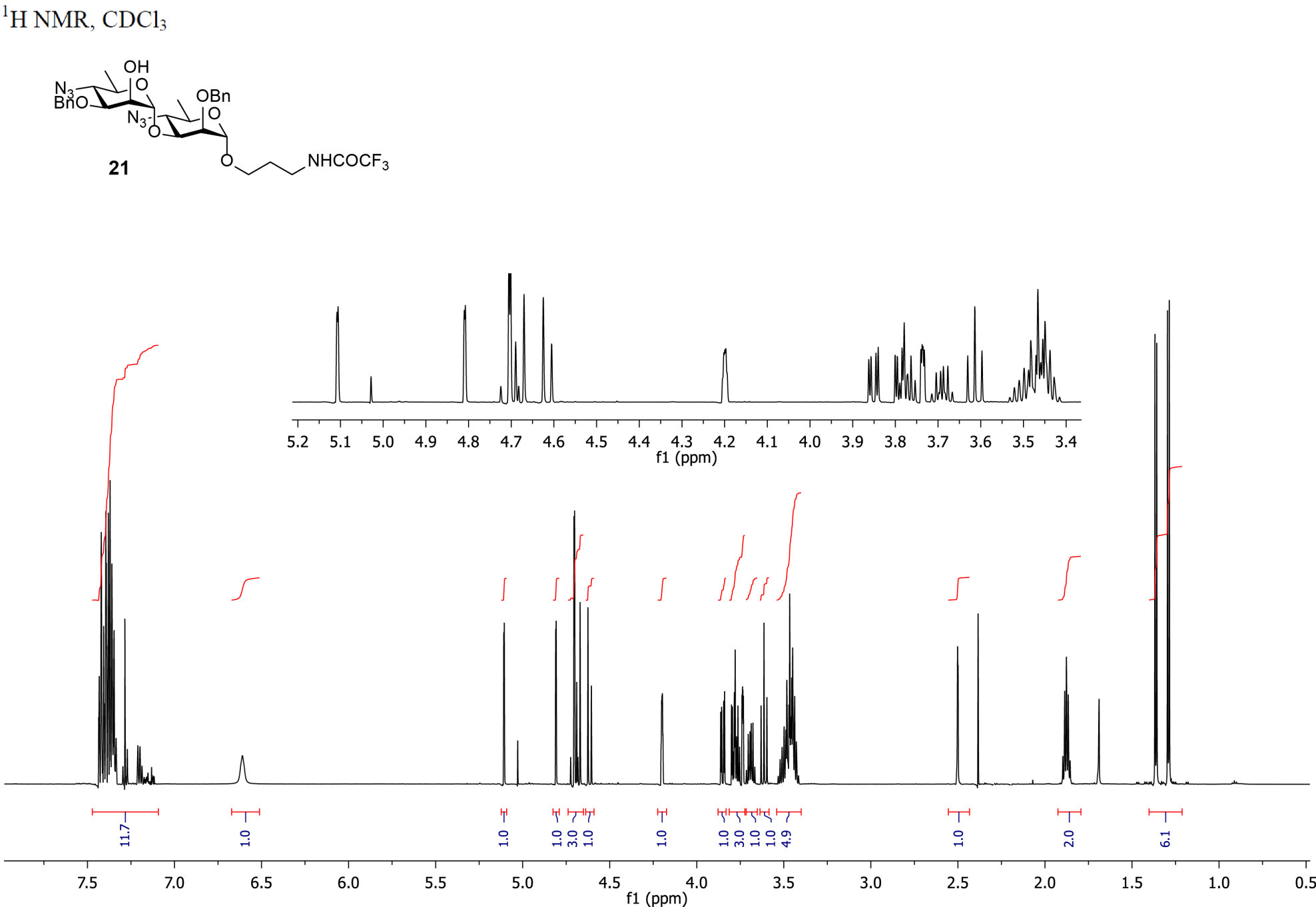


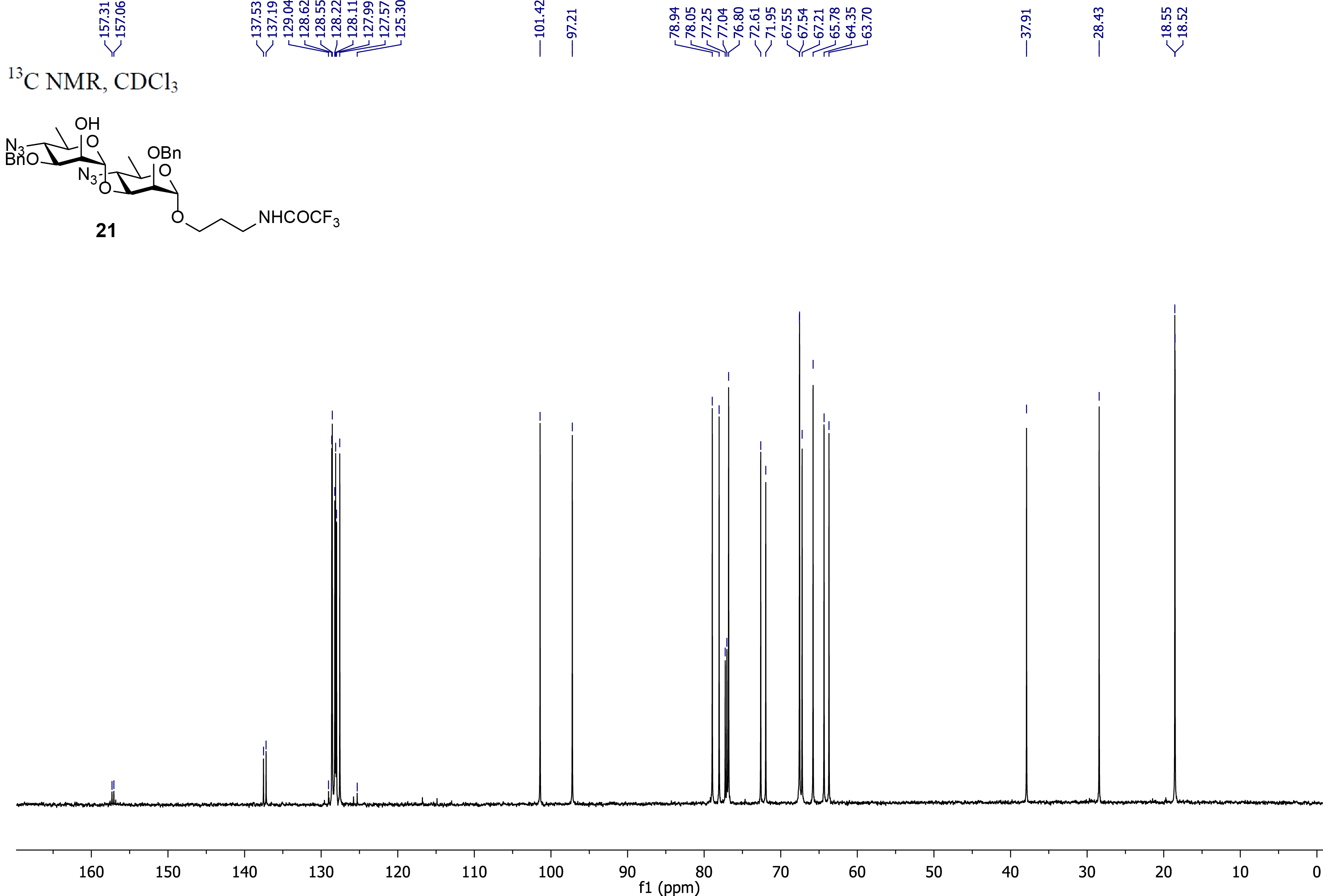


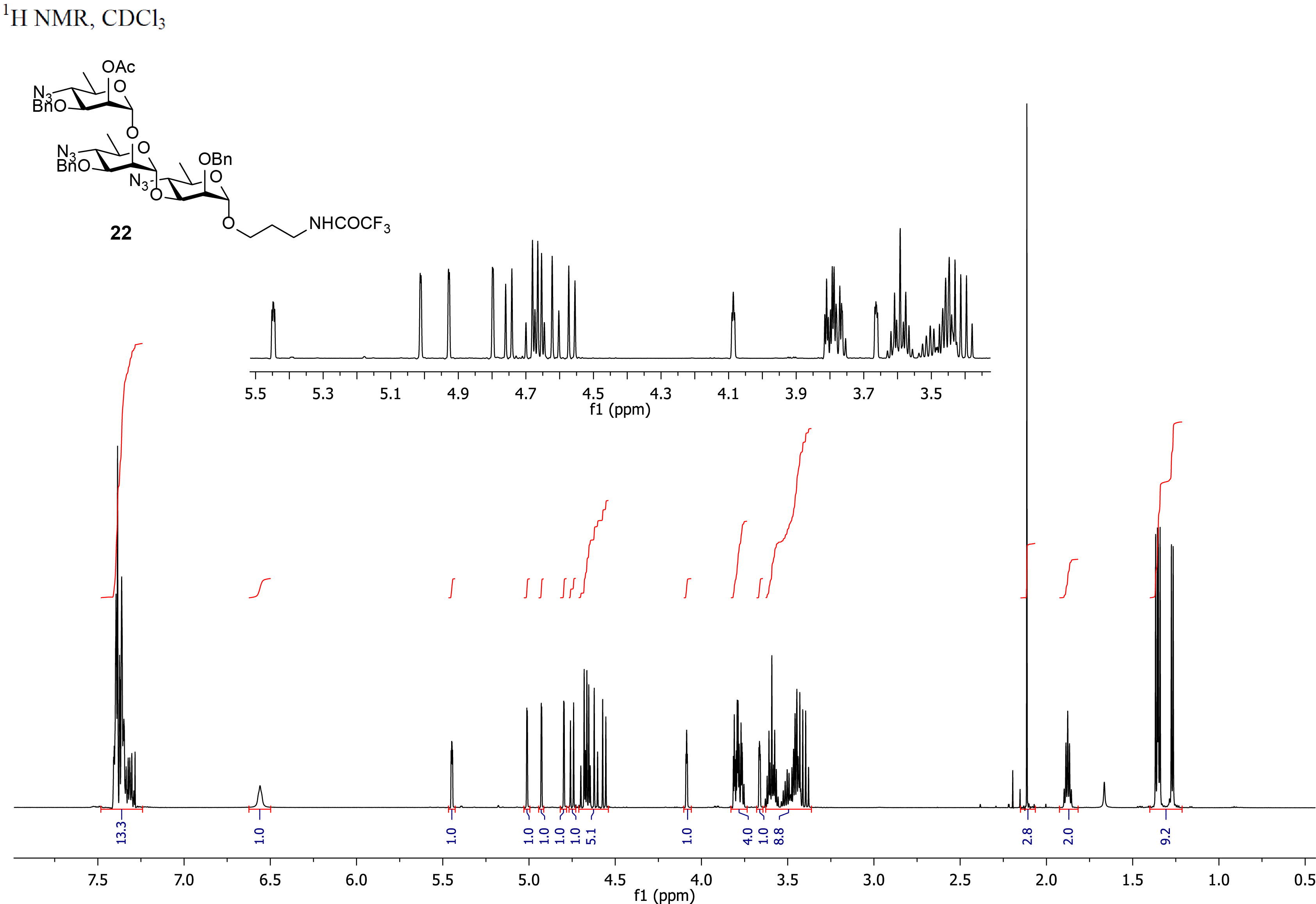

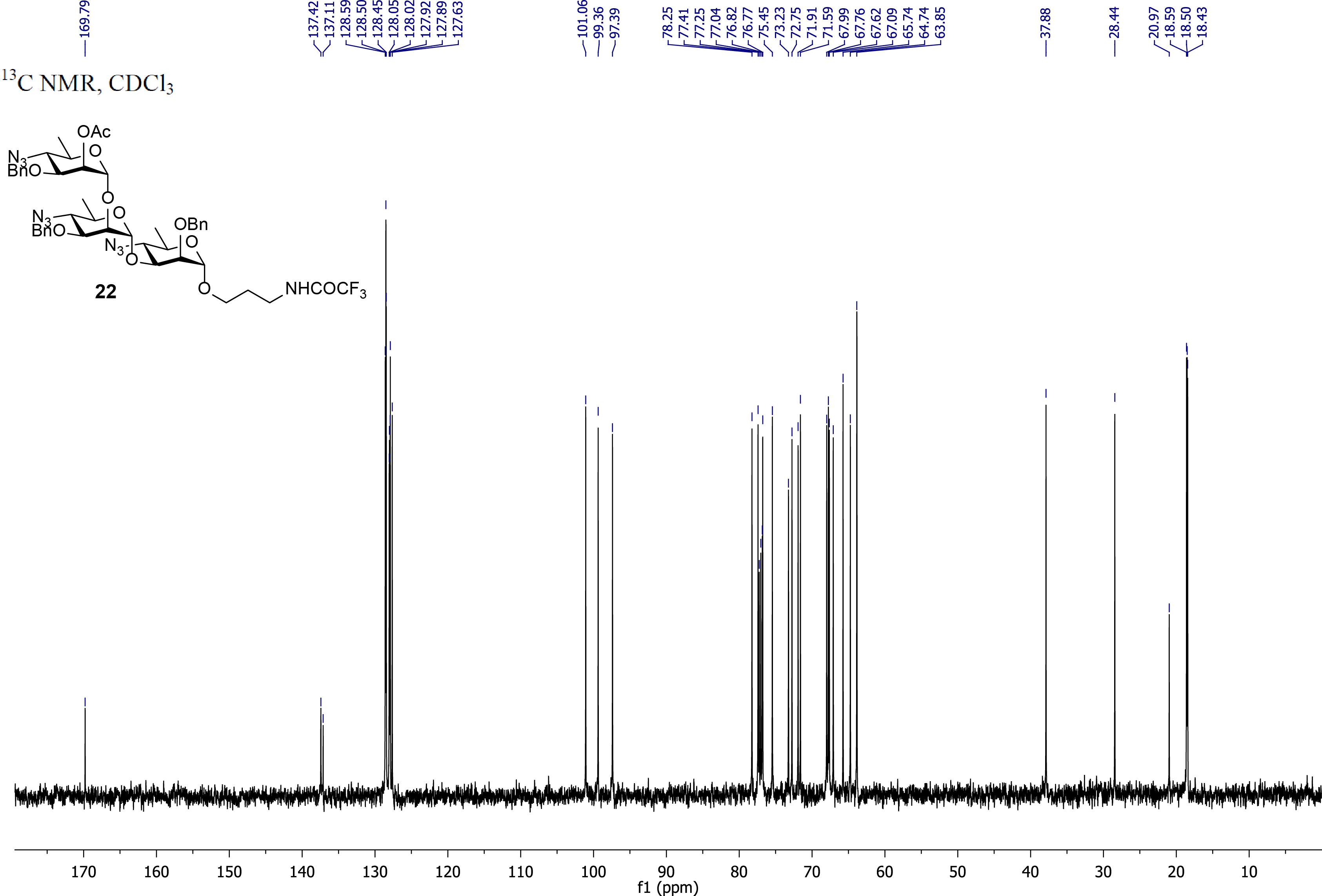


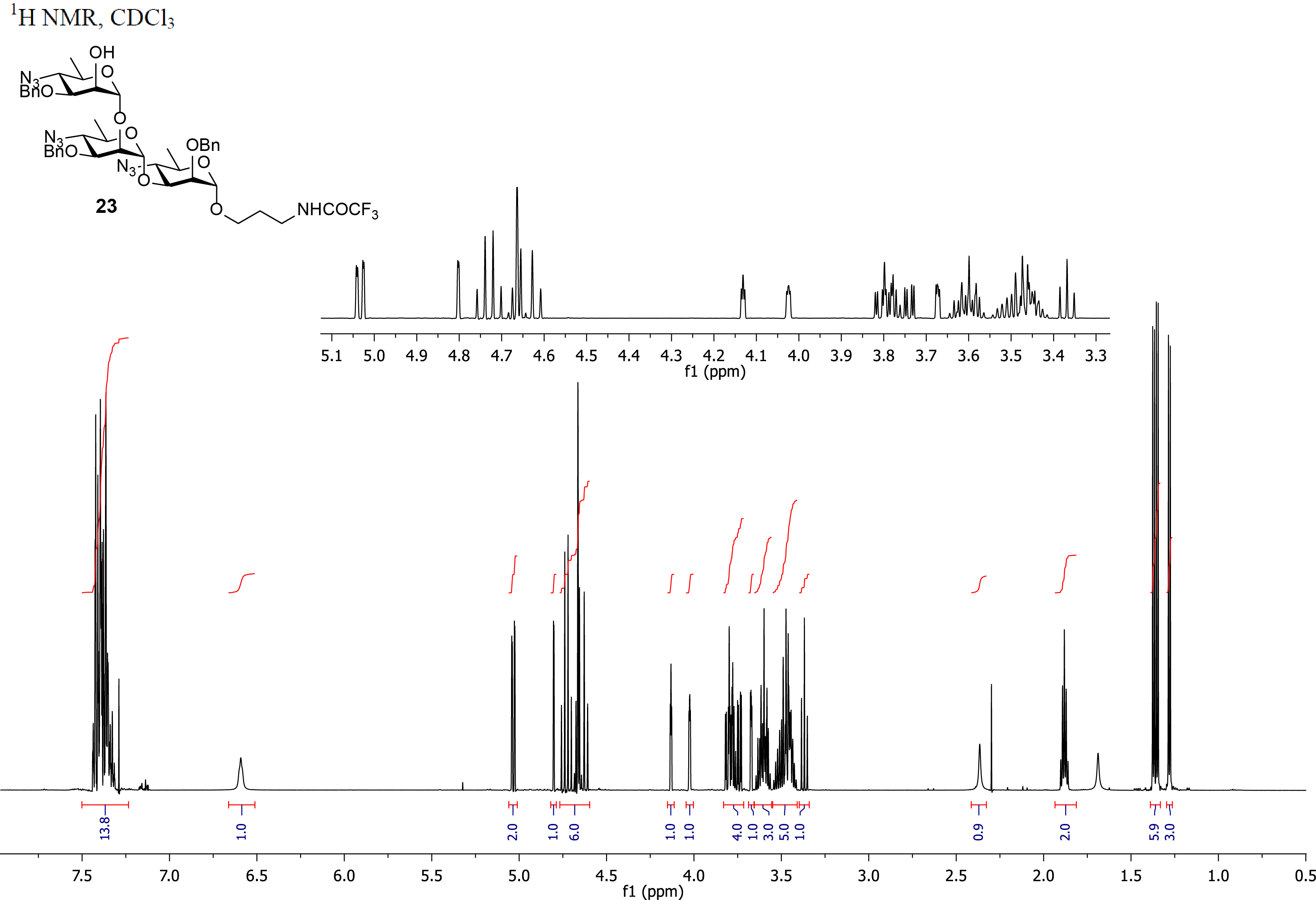


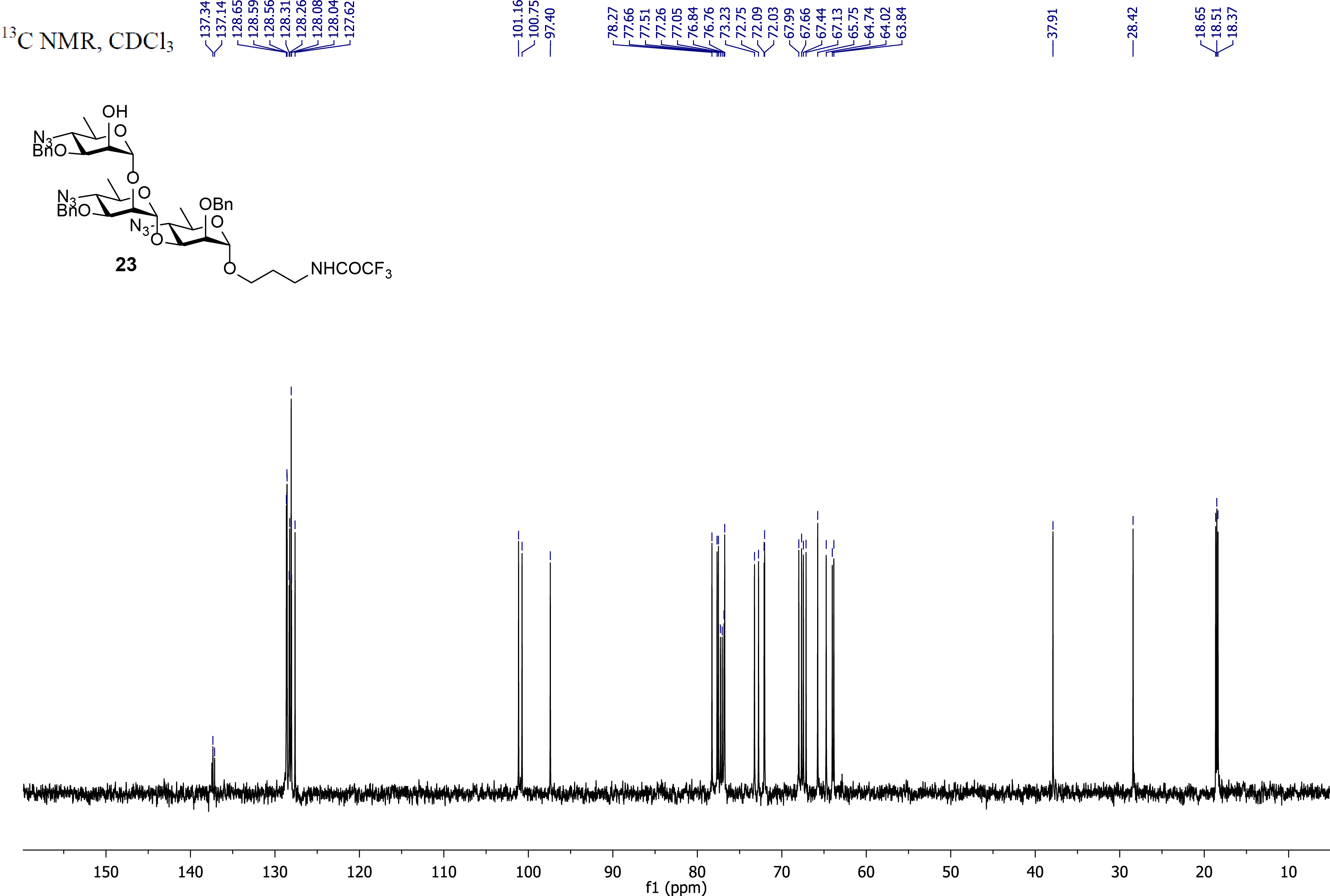


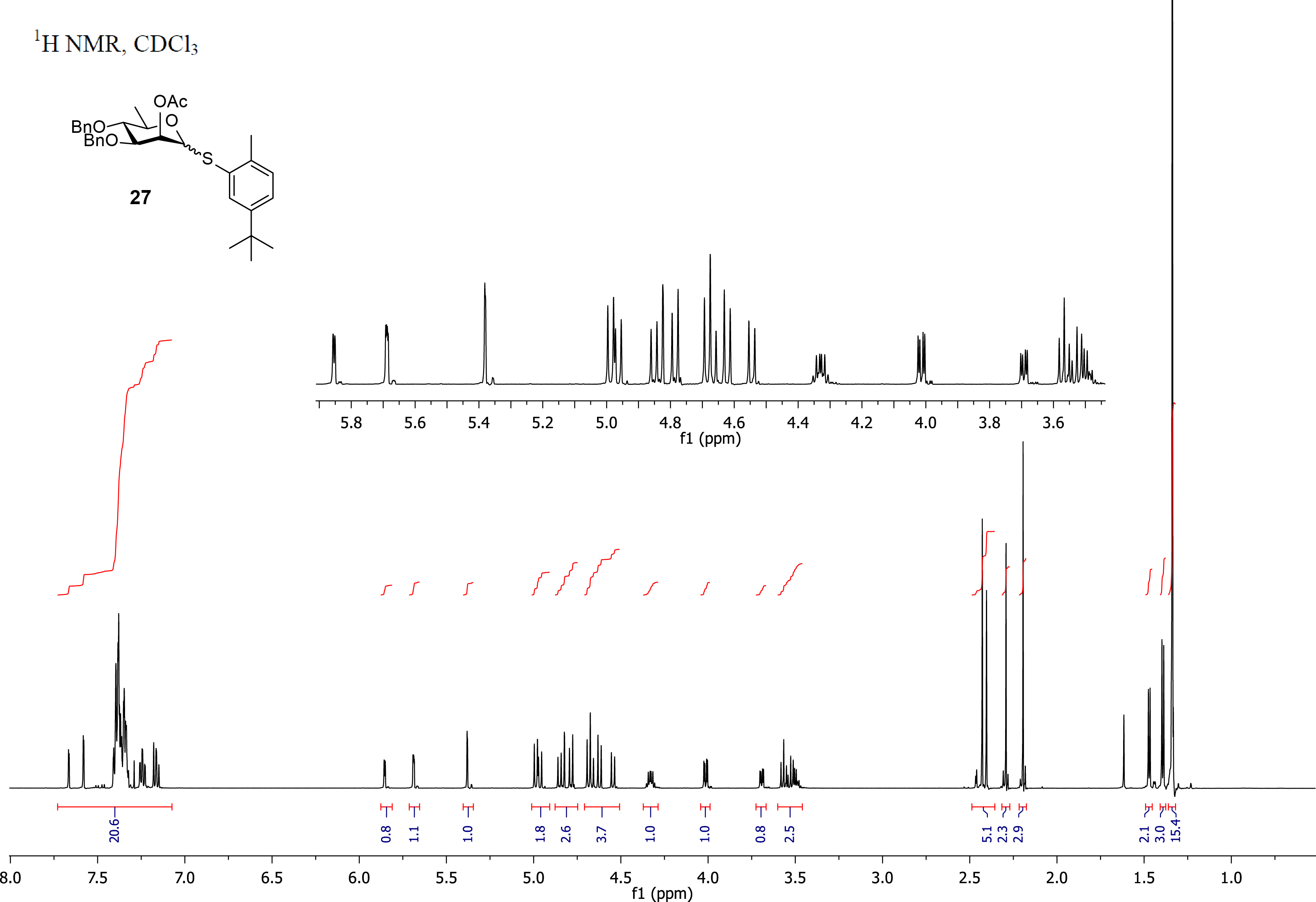


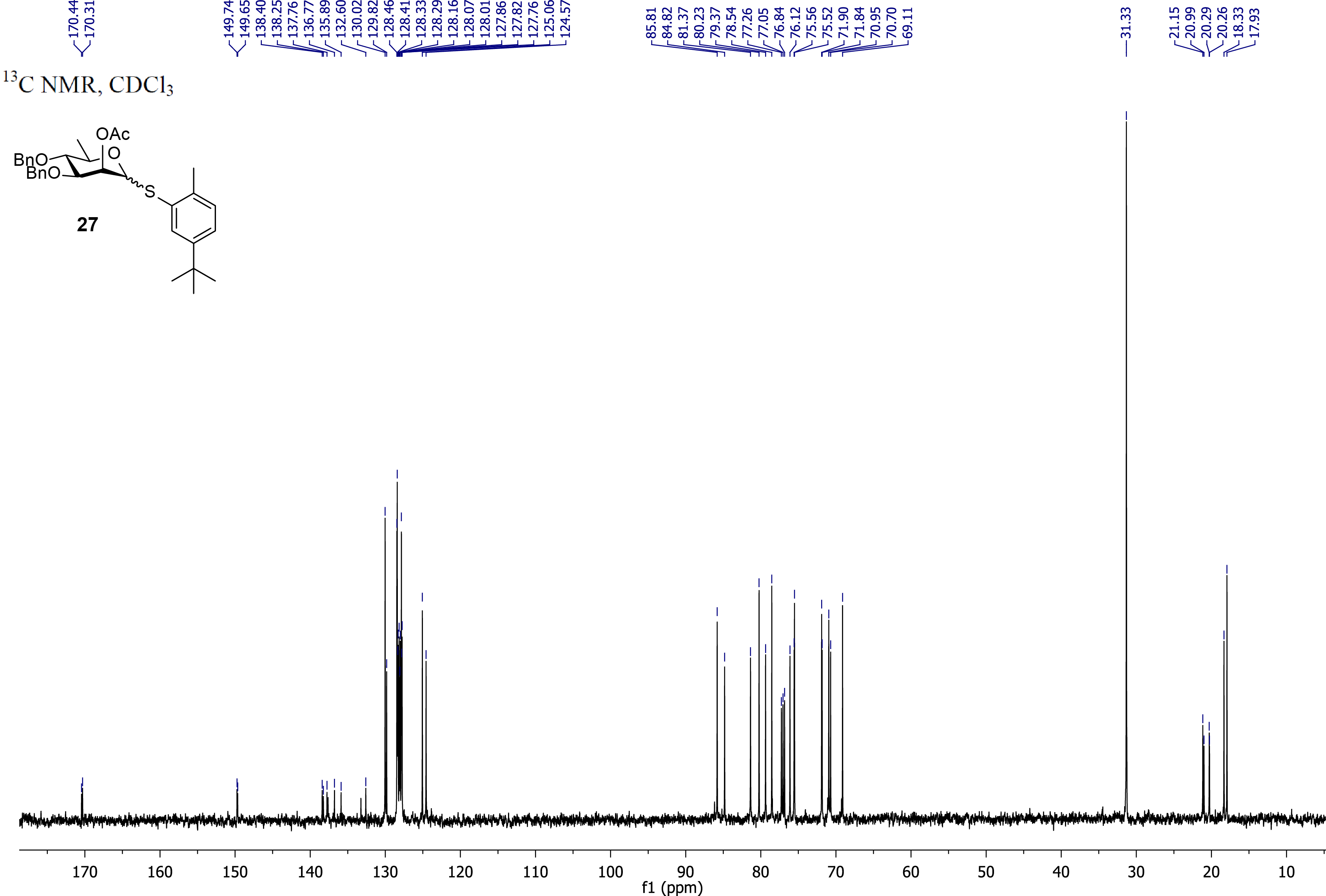


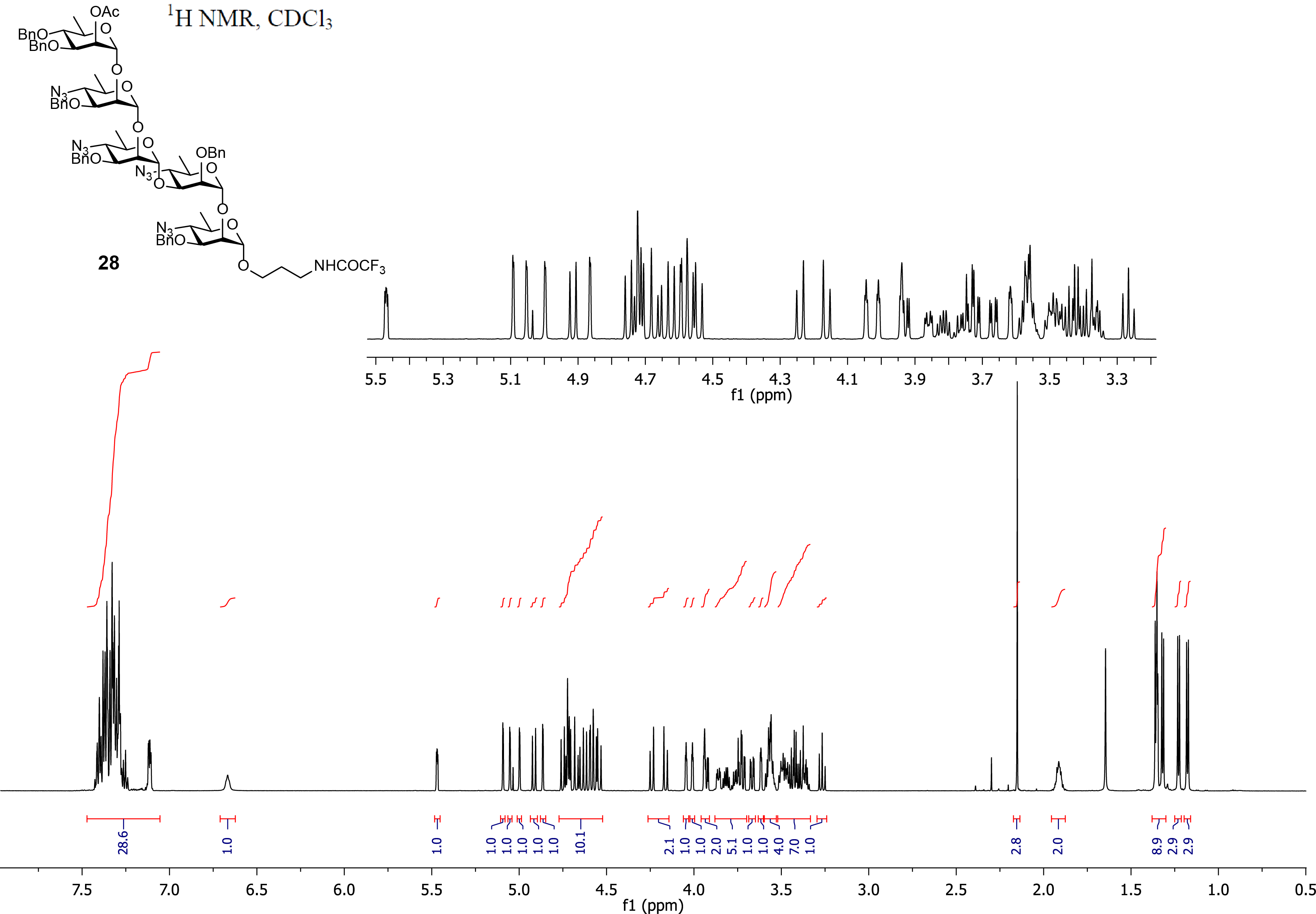


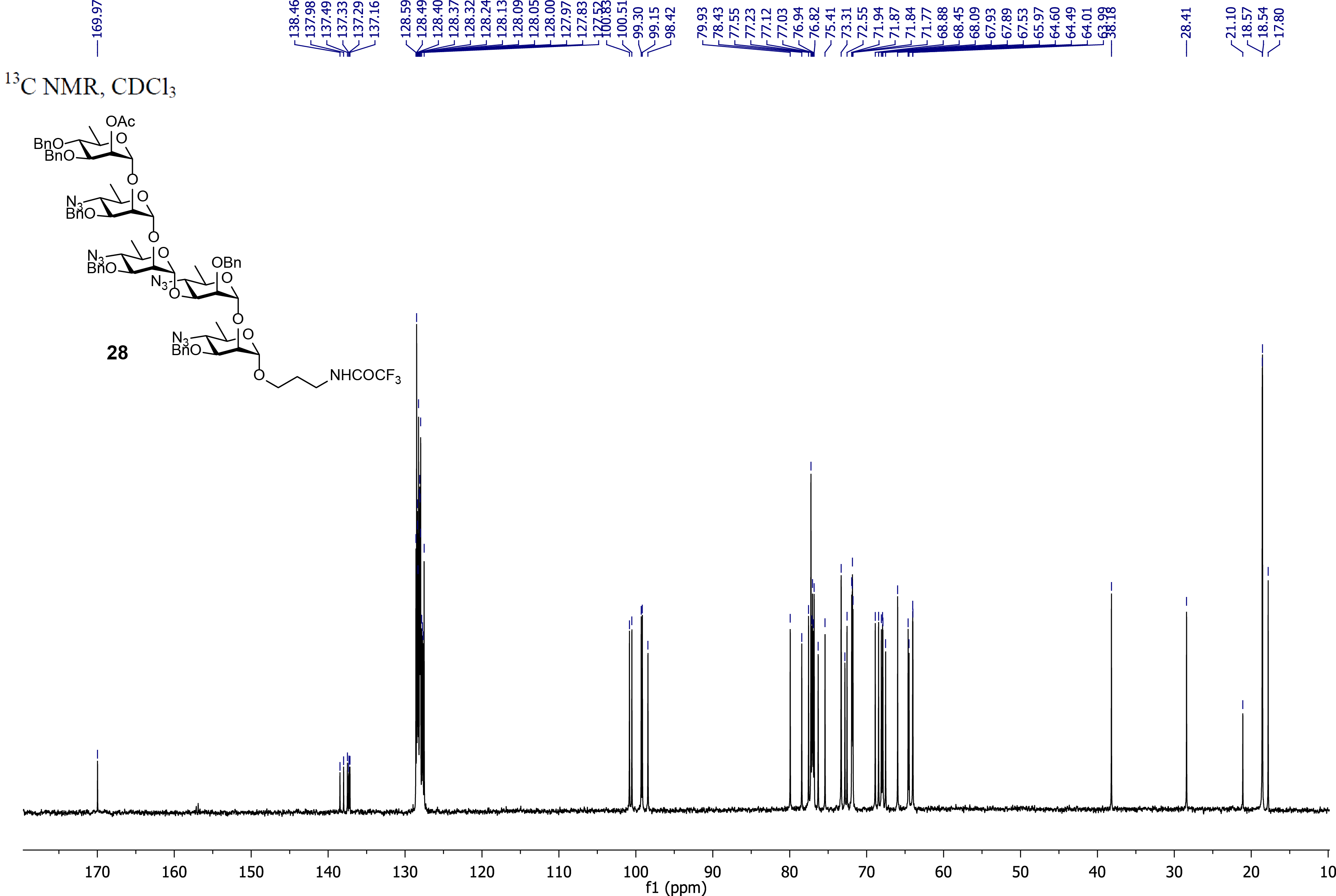


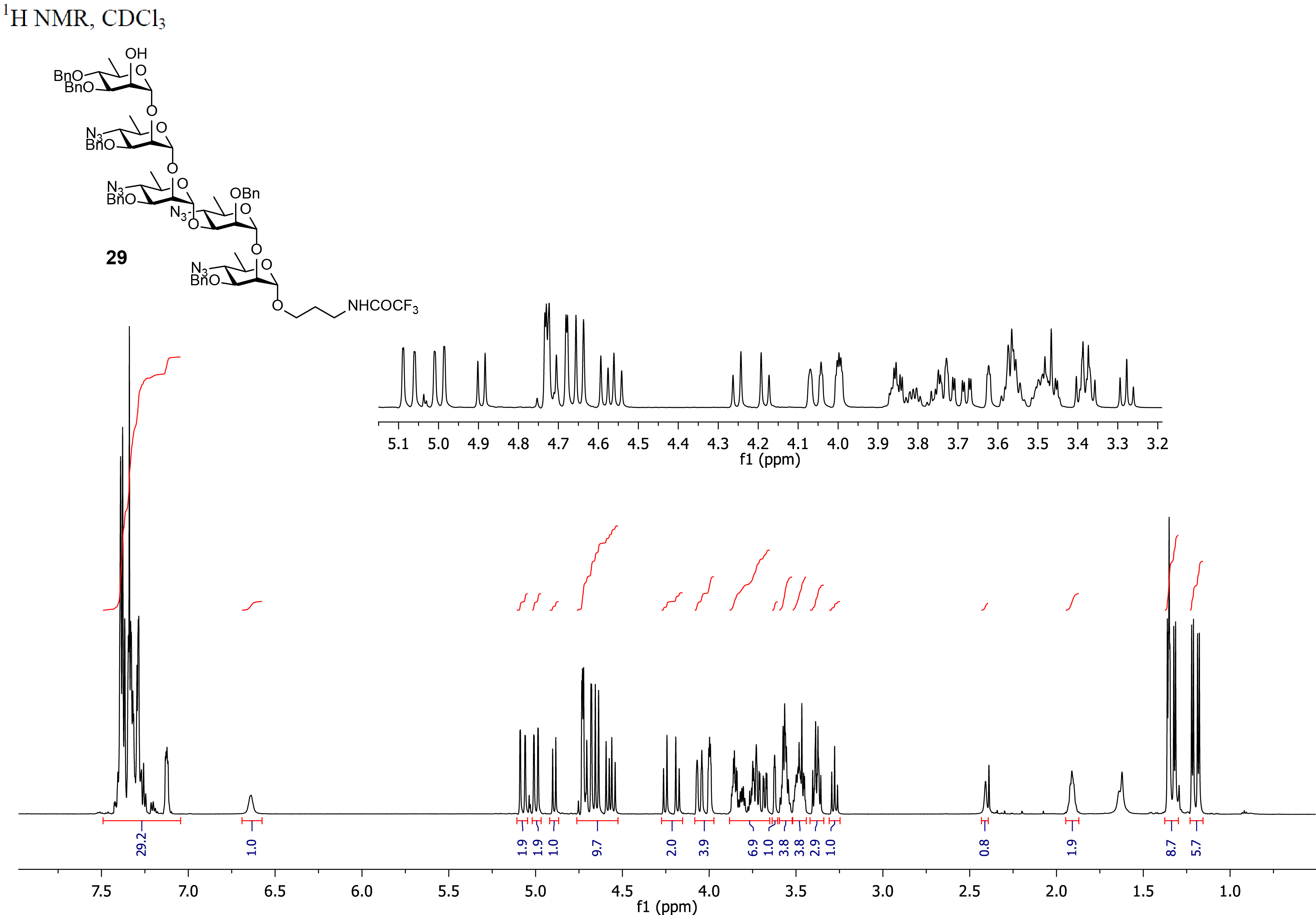


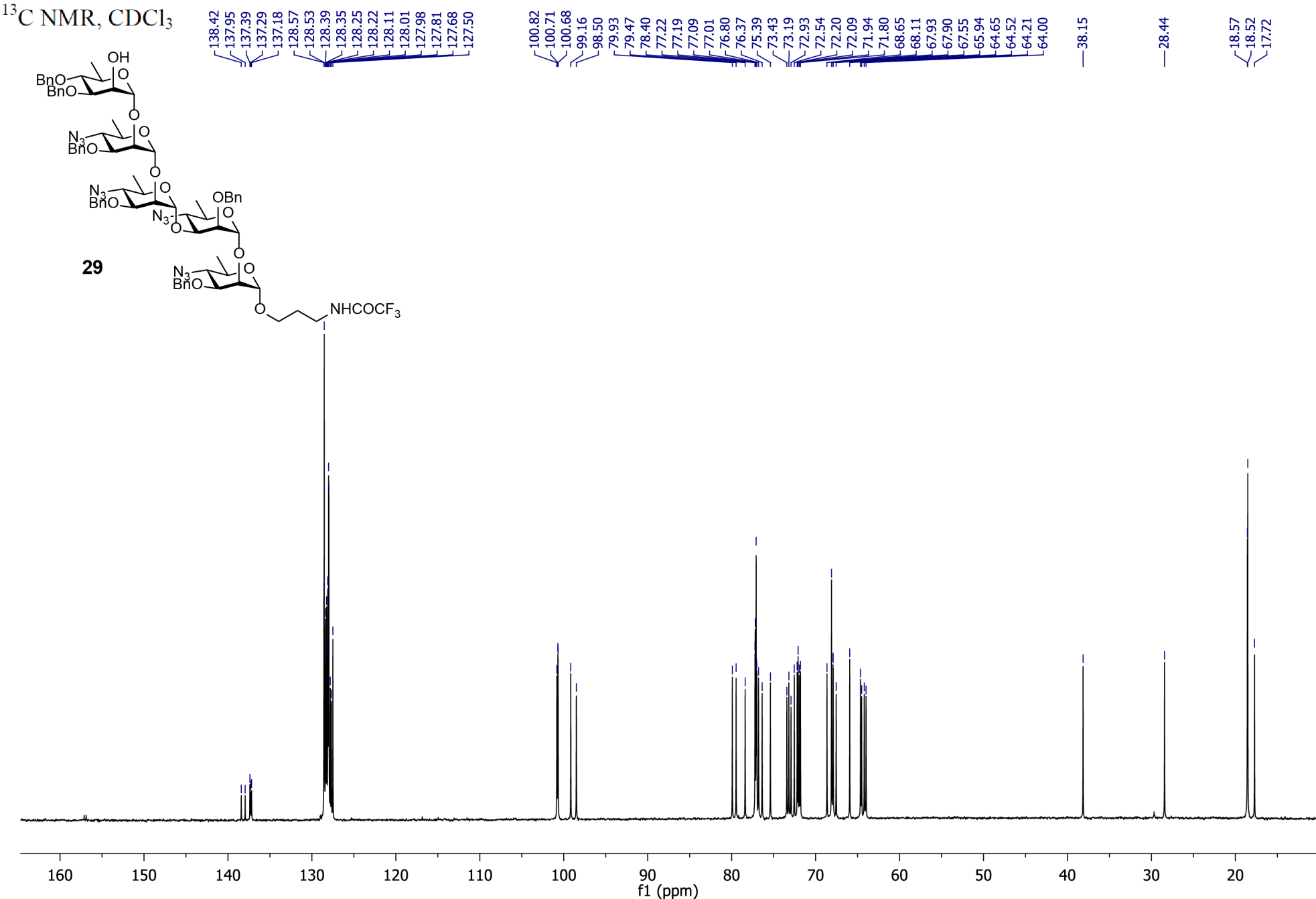


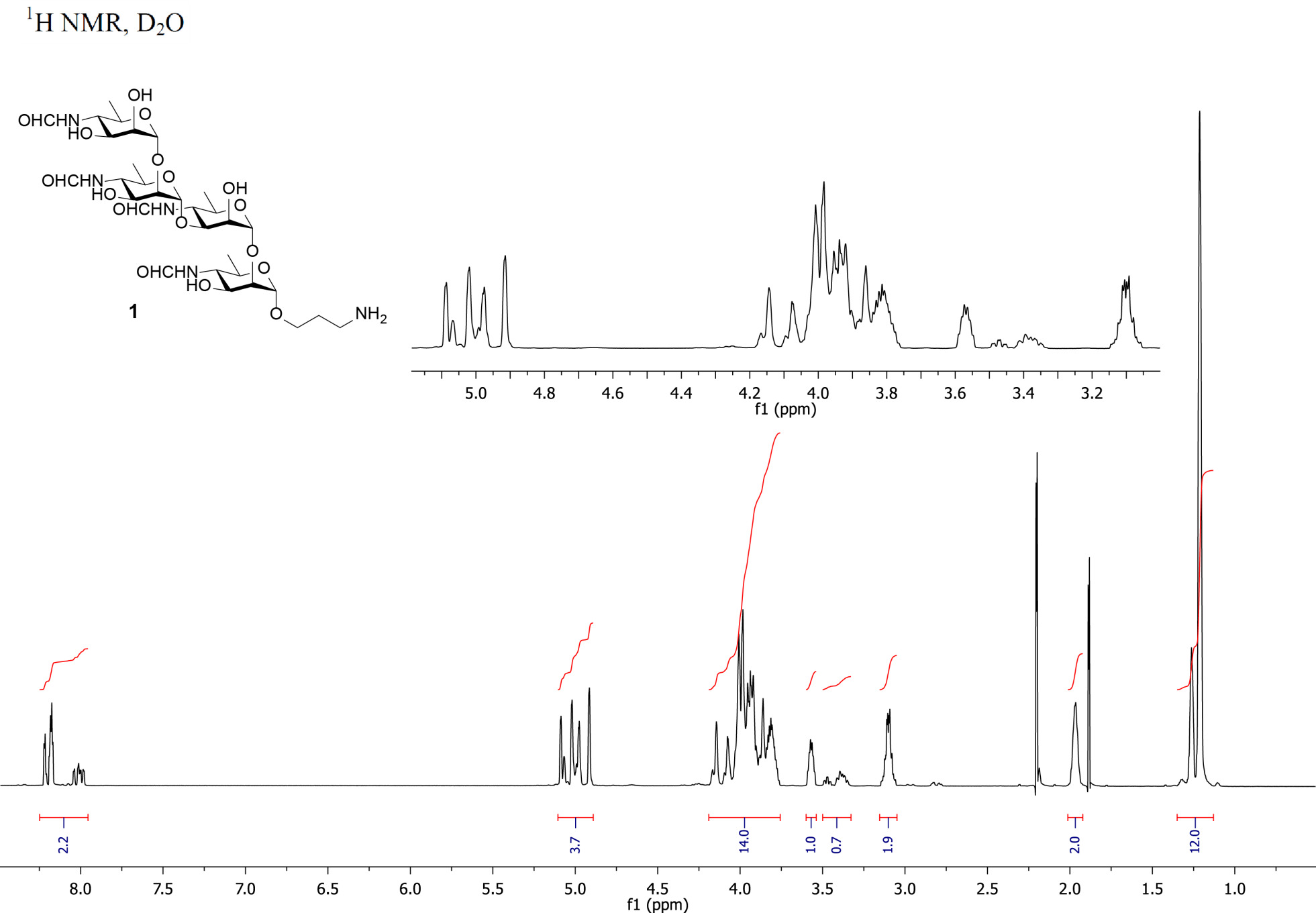


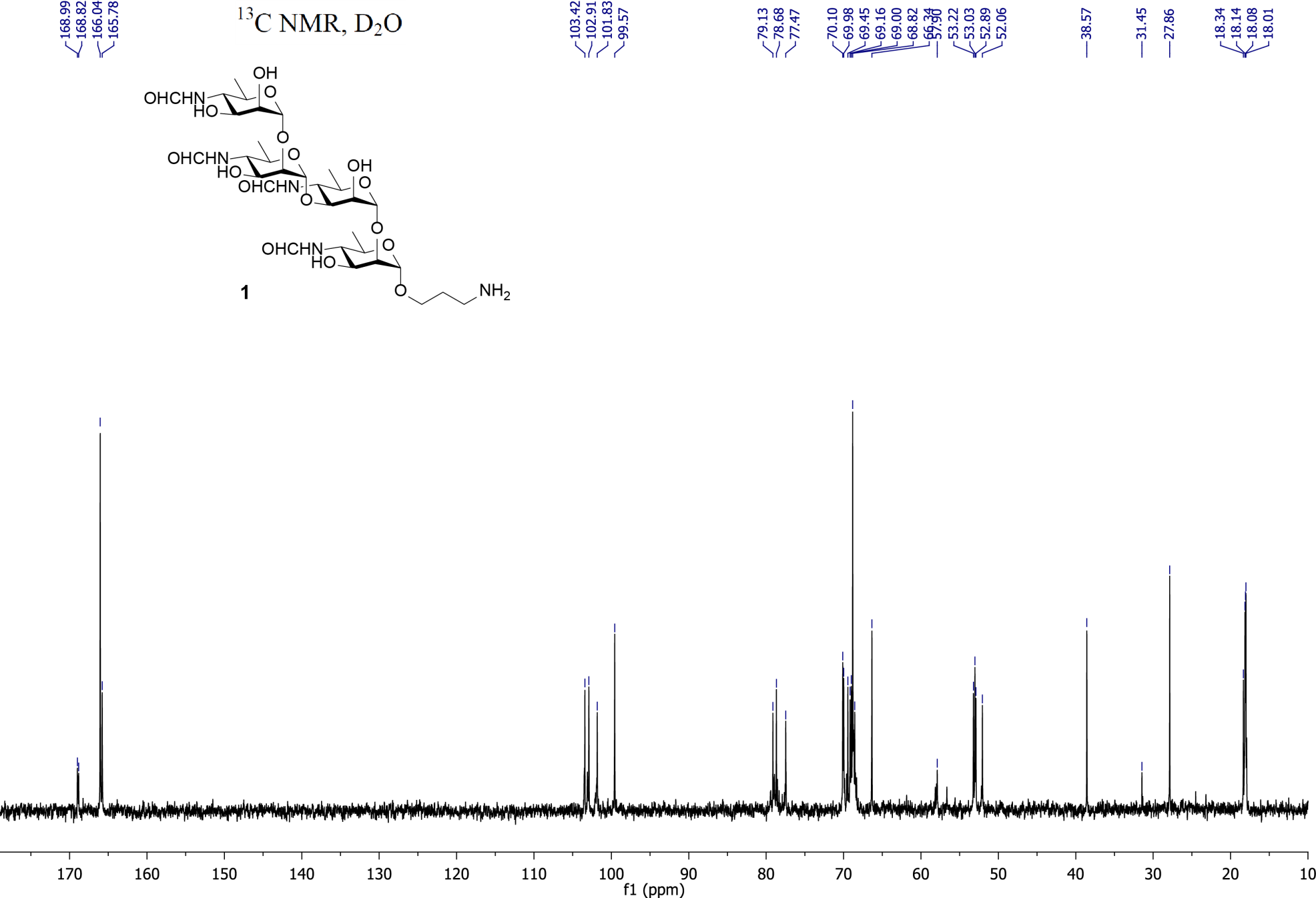


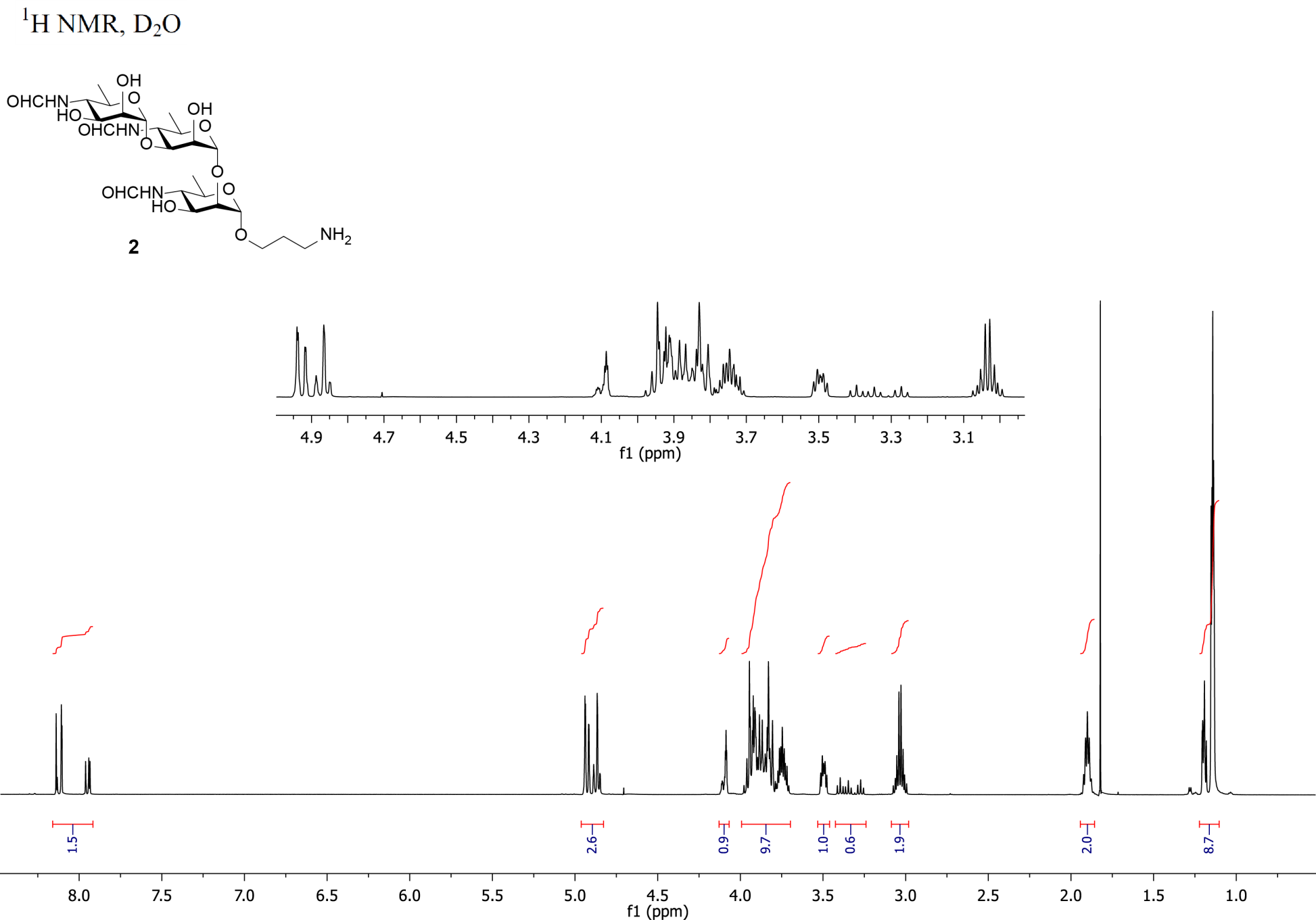


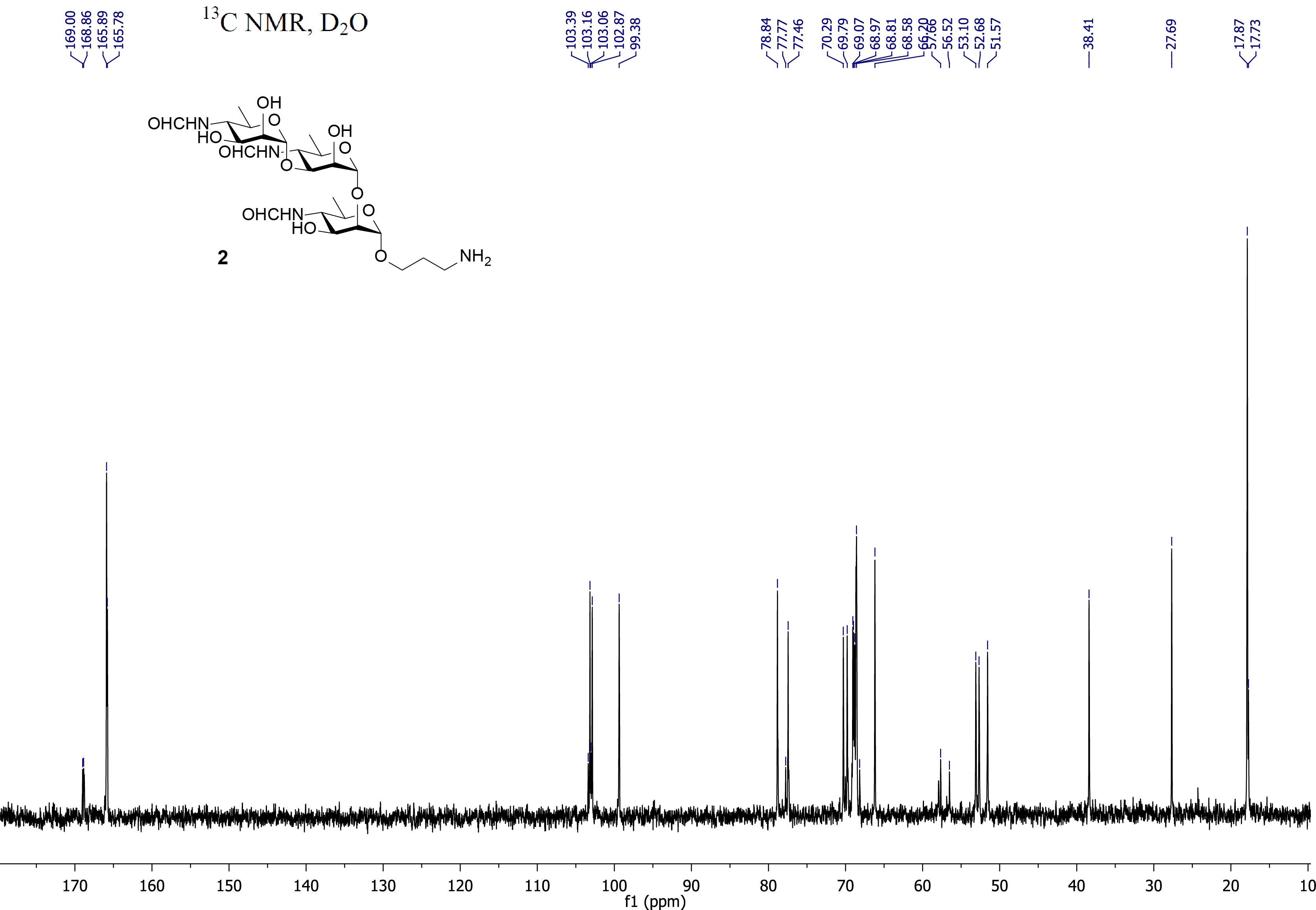


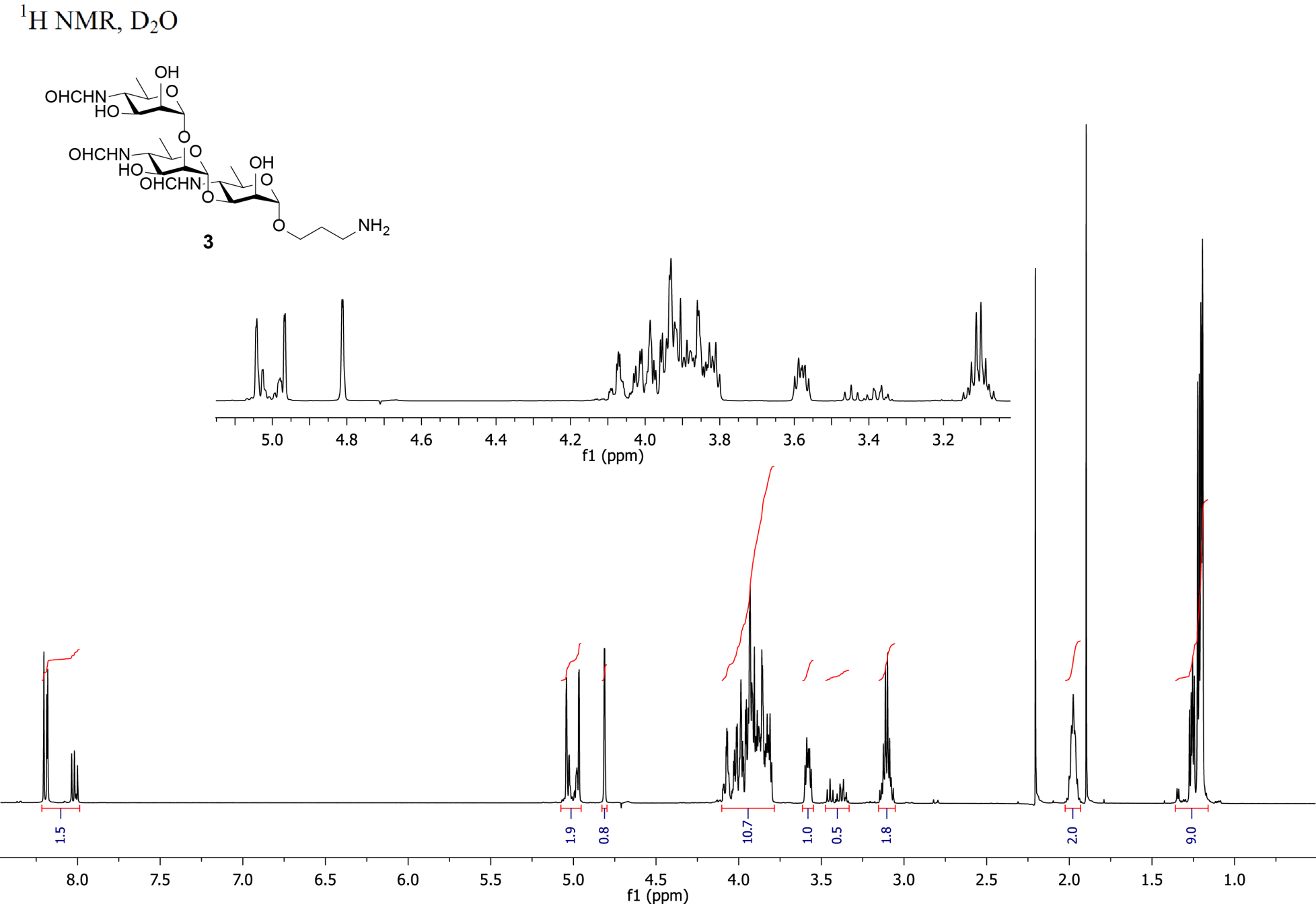


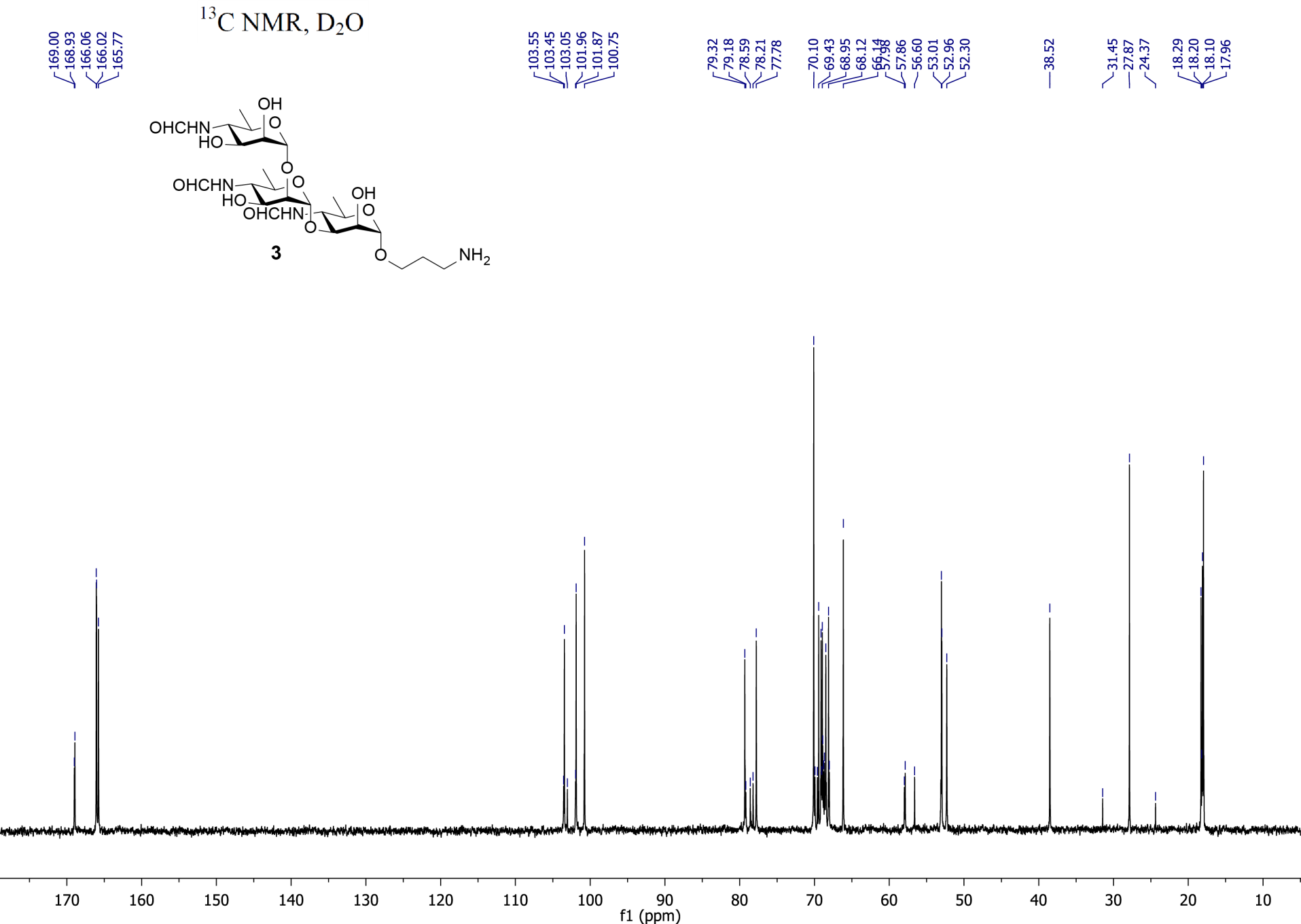

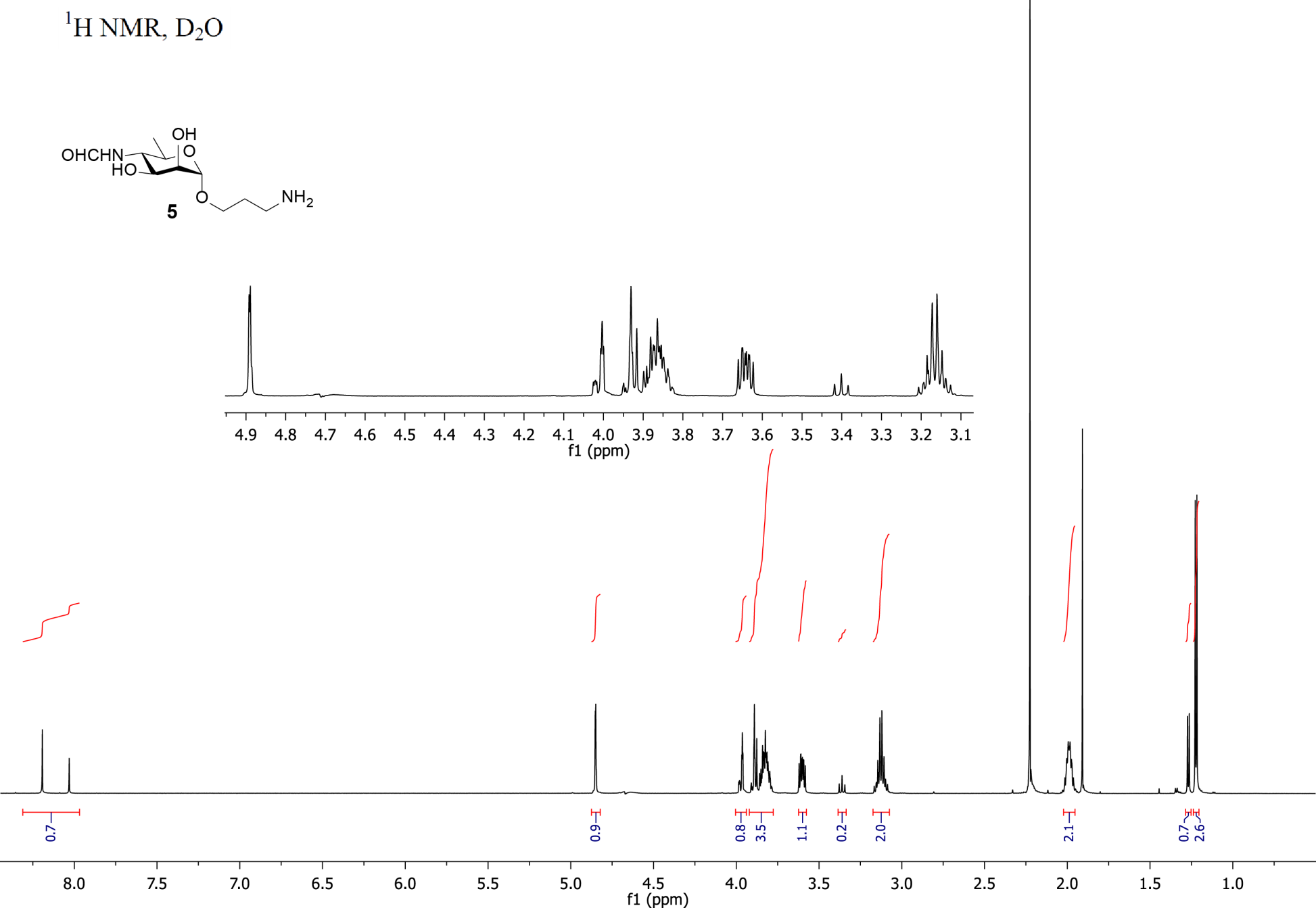


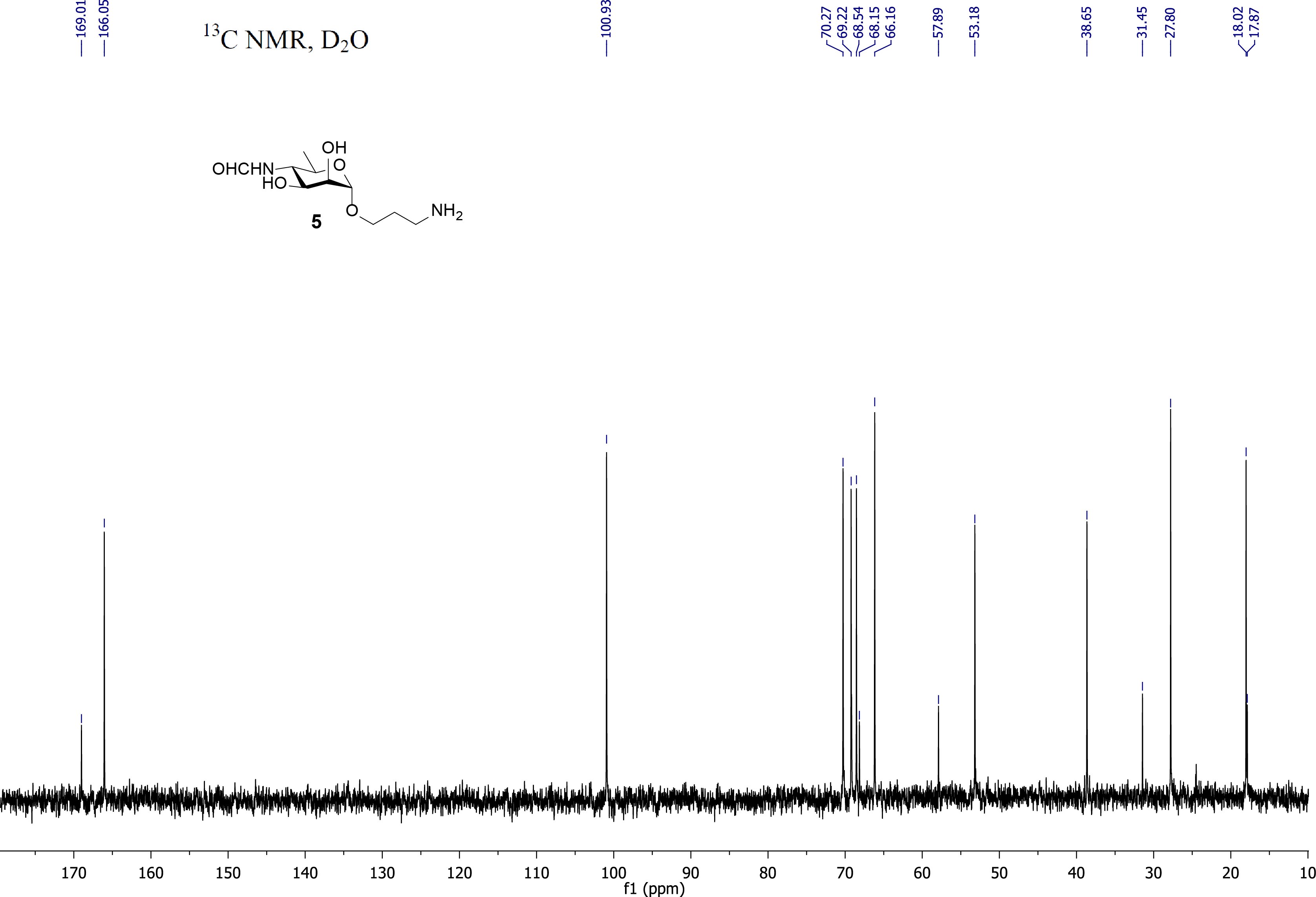


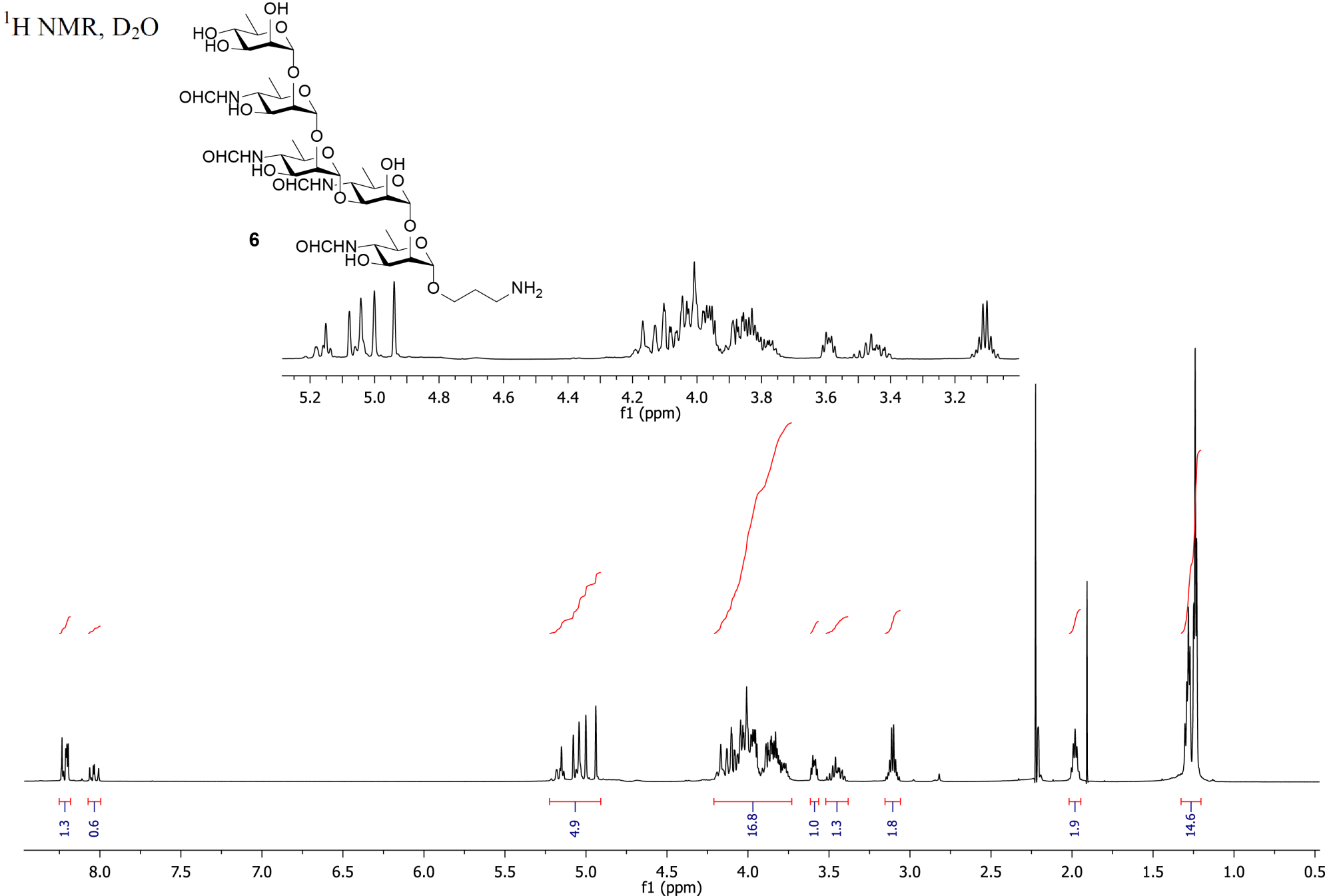


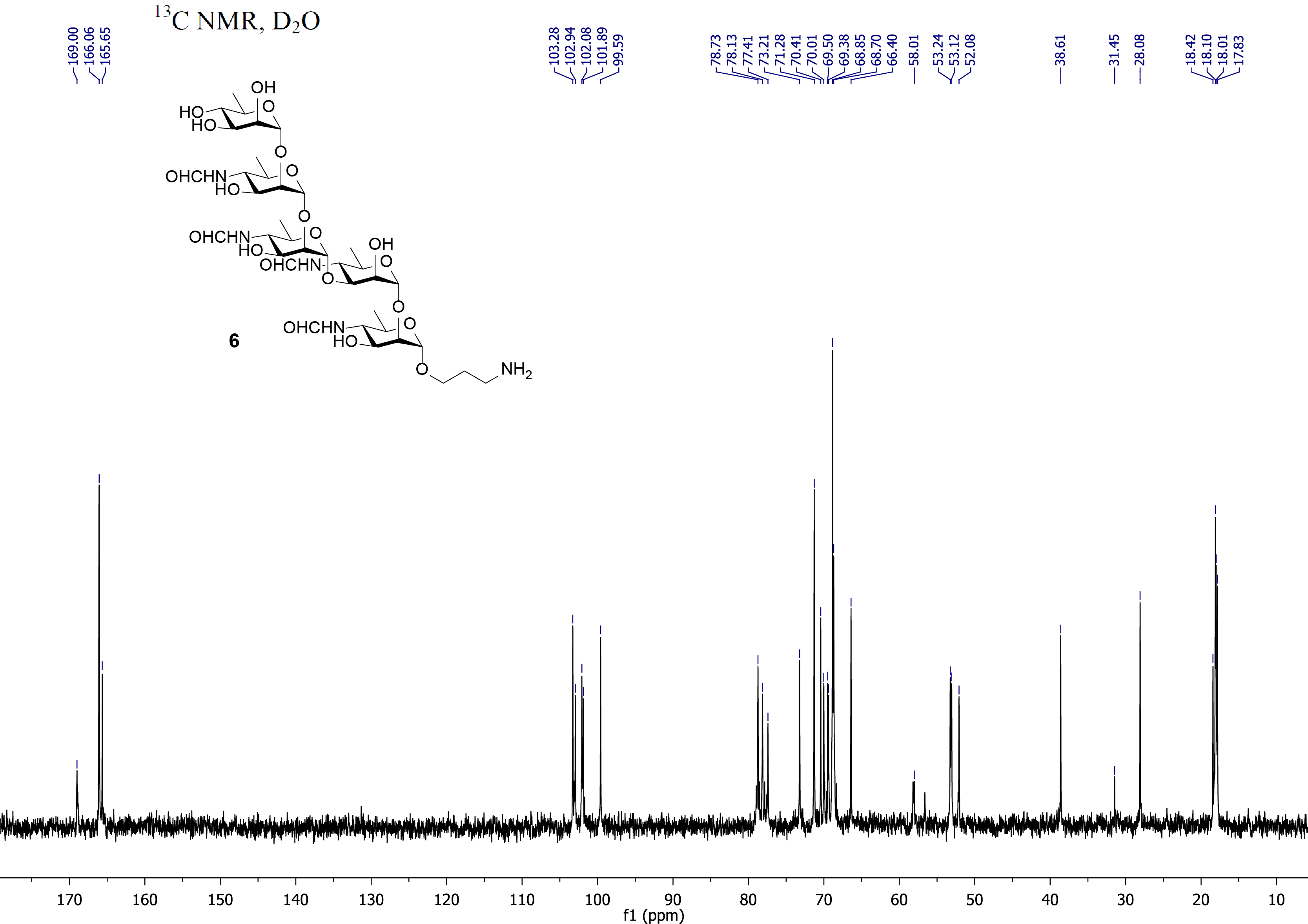

Supplement: Supplementary file 1 [file DataSheet1.docx]
